# A systematic framework for understanding the microbiome in human health and disease: from basic principles to clinical translation

**DOI:** 10.1038/s41392-024-01946-6

**Published:** 2024-09-23

**Authors:** Ziqi Ma, Tao Zuo, Norbert Frey, Ashraf Yusuf Rangrez

**Affiliations:** 1 Department of Cardiology, Angiology and Pneumology, University Hospital Heidelberg, Heidelberg, Germany; 2 DZHK (German Centre for Cardiovascular Research), partner site Heidelberg/Mannheim, Heidelberg, Germany; 3 Key Laboratory of Human Microbiome and Chronic Diseases (Sun Yat-sen University), Ministry of Education, Guangzhou, China; 4 Guangdong Institute of Gastroenterology, The Sixth Affiliated Hospital of Sun Yat-sen University, Guangzhou, China

**Keywords:** Microbiology, Cell biology

## Abstract

The human microbiome is a complex and dynamic system that plays important roles in human health and disease. However, there remain limitations and theoretical gaps in our current understanding of the intricate relationship between microbes and humans. In this narrative review, we integrate the knowledge and insights from various fields, including anatomy, physiology, immunology, histology, genetics, and evolution, to propose a systematic framework. It introduces key concepts such as the ‘innate and adaptive genomes’, which enhance genetic and evolutionary comprehension of the human genome. The ‘germ-free syndrome’ challenges the traditional ‘microbes as pathogens’ view, advocating for the necessity of microbes for health. The ‘slave tissue’ concept underscores the symbiotic intricacies between human tissues and their microbial counterparts, highlighting the dynamic health implications of microbial interactions. ‘Acquired microbial immunity’ positions the microbiome as an adjunct to human immune systems, providing a rationale for probiotic therapies and prudent antibiotic use. The ‘homeostatic reprogramming hypothesis’ integrates the microbiome into the internal environment theory, potentially explaining the change in homeostatic indicators post-industrialization. The ‘cell-microbe co-ecology model’ elucidates the symbiotic regulation affecting cellular balance, while the ‘meta-host model’ broadens the host definition to include symbiotic microbes. The ‘health-illness conversion model’ encapsulates the innate and adaptive genomes’ interplay and dysbiosis patterns. The aim here is to provide a more focused and coherent understanding of microbiome and highlight future research avenues that could lead to a more effective and efficient healthcare system.

## Introduction

The 2022 publication of the complete human genome sequence closed gaps from the Human Genome Project starting 20 years ago,^1–6^ and the recent “pangenome” draft further advanced our understanding of human genetic diversity.^7–9^ In symbiosis with the human body, the microbiome - a collective of microbes such as bacteria, fungi, archaea, viruses and their respective genomes, maintains a continuous crosstalk with the human genome. Exploring their interplay may elucidate a broader spectrum of individual phenotypic variations, considering that genomic differences between individuals account for only 0.1% of the total genome.^10^

Microorganisms were first discovered and reported by Antoni van Leeuwenhoek in the 17^th^ century using microscope.^11^ Advancements in modern techniques such as high-throughput sequencing, multi-OMICS, and artificial intelligence have greatly facilitated our understanding of the value of human microbiomes in health and disease. Notably, the Human Microbiome Project (HMP) and Integrative Human Microbiome Project (iHMP),^12–15^ European MetaHIT project (Metagenomics of the Human Intestinal Tract),^16–20^ American Gut Project (AGP),^21^ Dutch Microbiome Project (DMP)^22^ are prominent studies in this field. In the current landscape, microbial dysbiosis has gained significant recognition as a hallmark of both human health and the ageing process.^23–25^

To date, the overall understanding of the microbiome in the human body has been summarized in extensive classical and elegant reviews.^26–43^ Also, some conceptual terms have significantly enhanced our understanding of the human-microbe relationship. For example, concepts such as “holobiont”, “superorganism”, and “meta-organism” have expanded the definition of human.^44–46^ The “hologenome” frames the human genome and the genetic content of microbiomes as a single entity.^47^ The characterization of microbial physiological functions led us to consider them as another “organ”.^48^ Hypotheses like the “Hygiene Hypothesis”, the “Old Friends Hypothesis”, and the “Microflora Hypothesis” also prompted a reassessment of their immunomodulatory role.^49–51^ Despite their insightful contributions, the fragmented nature and limitations (will be discussed in the main text) of these hypotheses or theories have impeded a unified understanding of the microbiome.

To establish a systematic understanding of the role of the microbiome in human health and disease, this review first delves into the anatomical distribution and characteristics of the microbiome within the human body, elucidating its regulatory mechanisms on physiological functions. Among them, we introduce the concept of “acquired microbe immunity,” which synthesizes the microbiome’s “colonization resistance” and “immune modulation” functions. By further examining the physiological traits of germ-free animals, the complete knockout of microbial genomes, we termed “germ-free syndrome”. The abnormalities resulting from the loss of the microbiome further prompt us to explore the integrity of the human genome parts through the lens of genetics and evolution. Herein, the “adaptive genome” refers to the external and dynamic microbiome, while the “innate genome” denotes the inherent genetic blueprint that humans are born with. The introduction of the “adaptive genome” concept allows us to extend the notion of a single host to that of a “meta-host”, thereby gaining a comprehensive understanding of disease heterogeneity or the success rate of organ transplantation resulting from host-microbiome interactions. To address the complex interplay of physiological dependence and conflict with microbes, the hypothesis of “slave tissue” was introduced, viewing the microbe as an exogenous tissue under the control of human master tissues such as nerve, connective, epithelial and muscle tissues. Recognizing that homeostasis theory is fundamental to understanding health and disease, we further discuss the hypothesis of “homeostasis reprogramming” based on the theoretical foundation of the adaptive genome and slave tissue. Utilizing the “cell-microbe co-ecology model,” we describe the phenomenon of co-homeostasis between microbes and human cells. Lastly, to deepen our understanding of how microbes contribute to disease, a “health-disease conversion model” was proposed, outlining the common patterns of dysbiosis. To conclude, the above envisioned coherent and systematic conceptual framework is expected to bolster the effectiveness and efficiency of the healthcare system.

## Human microbial distribution, development, personalization, and stabilization

**Fig. 1:**
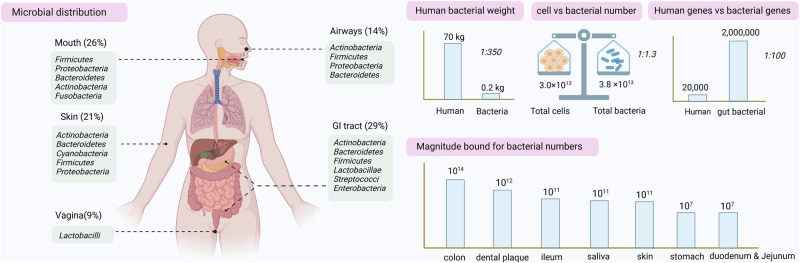
Human microbial-related characteristics. The distribution data of the microbiome were obtained from the Human Microbiome Project,^62,657^ supplemented by modified data from Ron Sender et al.^658,659^

The human body is inhabited by diverse microorganisms including bacteria, fungi, archaea and viruses (bacteriophages). Throughout long human history, microorganisms have co-evolved with us,^52–61^ exhibiting periodic variations that align with the different stages of a person’s life.^37^ These microbes are mainly found in the mucosal and superficial layers of organs and can interact with the environment. Of the known bacterial distributions, the gastrointestinal tract is the most densely populated (29%), followed by the oral cavity (26%) and skin (21%), while the respiratory tract (14%) and urogenital tract (9%) have lower densities^62^ (Fig. 1). Microbial communities also show density gradients within specific organs, such as higher densities in the upper respiratory tract than in the lower respiratory tract, and lower densities in the stomach, duodenum, and jejunum than in the ileum and colon.^62^

### Emerging insights in traditionally sterile human sites

Anatomical sites traditionally considered sterile in human anatomy are now being challenged by the emergence or potential existence of resident microbiota, albeit with some controversy. The environment on diseased blood vessels is non-sterile, containing bacteria and viruses, with the sequencing of arterial atherosclerosis providing compelling evidence.^63^ Alison Clifford et al. extensively discussed the presence of normal vascular microbiota, but the evidence is largely from non-viable samples.^64^ Using strict exclusion criteria, aseptic sampling, repeated measures, and negative controls to eliminate potential contamination, László Hidi et al. analyzed microbiomes in femoral arteries from brain-dead donors, mainly those with hemorrhagic or ischemic strokes.^65^ They identified *Proteobacteria*, *Firmicutes*, and *Actinobacteria* as the predominant phyla, with *Staphylococcus*, *Pseudomonas*, *Corynebacterium*, *Bacillus*, *Acinetobacter*, and *Propionibacterium* being prevalent genera.^65^ Additionally, they observed a notable correlation between blood type and microbiota diversity.^65^ Although limitations such as the small sample size (14 participants) and the older age range of donors (40–60 years) reduce the power of this study and the lack of characterization of microbial function,^65^ it however provides valuable insights into the possible presence of the microbiome in the normal human vasculature and potential research directions for unraveling vasculature-based diseases. Interestingly however, despite previous findings of approximately 1% of the human body’s bacterial presence in blood samples from the Human Microbiome Project, an analysis of 9770 samples from healthy individuals revealed the absence of similar microbial communities in the bloodstream.^62,66^ Notably, 82% of the sampled population exhibited no microbial sequences, emphasizing the sterile nature of the blood in healthy individuals.^66^ A diverse microbiome is also found on the human ocular surface, with *Pseudomonas*, *Bradyrhizobium*, *Propionibacterium*, *Acinetobacter*, and *Corynebacterium* being the most abundant genera.^67–70^ Similarly, the brain has long been considered a sterile organ because of its blood-brain barrier and immunity.^71^ However, surprising findings from the microscopic examination of multiple brain regions post-mortem in healthy individuals, presented at a neuroscience conference, have revealed the existence of microbiota in the brain.^72^ Nevertheless, convincing evidence awaits confirmation using animal models and independent human material.^73^ Certain organs responsible for the secretion of body fluids also contain microbiota. For example, although sampling difficulties exist, a study on healthy humans confirmed the presence of microbial community in the gallbladder which may retrograde entry of gastrointestinal microbiota.^74^ In women aged 18–90, a diverse range of bacteria, predominantly belonging to *Proteobacteria*, have been identified in mammary tissue.^75^ The question of whether a normal fetus is colonized by microbes in the prenatal environment (“in utero colonization” hypotheses), challenging the assumption of uterine and placental sterility (“sterile womb” hypotheses), remains controversial.^76–81^ A recent comprehensive discussion suggests that the detected microbes may be due to contamination, emphasizing the lack of reliable evidence for the presence of microbial colonization.^82^

### Colonization and development of common microbiomes

Although microbial sampling can't cover every anatomical niche or the complete microbiome, including bacteria, viruses, and fungi, throughout an individual’s entire lifespan, microbiome composition is influenced by various factors like host and environmental factors (which will be discussed in the section on adaptive genome), substantial progress has been made in recent decades in sequencing the microbiomes of the gut, oral cavity, skin, vagina, and lung. Overall, microbiota development initiates at birth, with the primary succession phase characterized by rapid microbial changes that decelerate into a more stable “climax community” by adolescence.^83^ This community, while relatively stable, can still experience fluctuations in adulthood.^37^ Disturbances such as antibiotics or infections may prompt secondary succession, potentially leading to a new microbial state.^84^ Finally, in old age, the microbiota undergoes final succession, typically resulting in a community with reduced diversity.^37^ Below, we will provide a brief overview of the microbiota throughout the lifespan by integrating current knowledge. While much of the information has been comprehensively reviewed,^37,85,86^ we include some of the latest research findings.

### Digestive tract microbiome

In the early stages, newborns acquire pioneer microorganisms from their mother’s vaginal tract, skin and possibly through fecal exposure during the birth process. The gut bacteria are initially dominated by *Bifidobacterium spp*. and gradually shifts to a mixed community of *Bifidobacterium*, *Clostridium* and *Bacteroides spp*. by the end of the first year.^87^ This shift is accompanied by a greater diversity of genera within the *Firmicutes*, including *Clostridia*, *Faecalibacterium*, *Ruminococcus* and *Veillonella*, while the abundance of *Bifidobacterium spp*. decreases.^15^ At approximately 3 years of age, the gut microbiota stabilizes and is dominated by *Firmicutes* and *Bacteroidetes*.^88^ However, the majority of the studies to date are largely based on easily accessible fecal samples, and endoscopic examinations are also limited by single-site sampling in certain areas.^80^ To study the distribution of microbiota across multiple regions of the human intestinal tract under undisturbed and uncontaminated conditions, in 2023, Dari Shalon et al. developed an ingestible capsule device capable of sampling four specific sites from the small intestine to the ascending colon suggesting that specific microbial phyla may be enriched in specific intestinal segments compared to fecal samples.^89,90^ A more detailed and rigorous identification was conducted by Jun-Jun She et al. who sampled seven surface organs of deceased individuals within 1.5 h postmortem.^91^ In their study, *Helicobacter* species were found to be enriched in the esophagus and can also be found in the stomach, where it likely contributes to the fatty acid metabolism alongside *Lactobacillus*.^91^ *Prevotella*, which accumulates in the duodenum, is potentially involved in the degradation of carbohydrates and amino acid synthesis.^91^ *Enterococcus* and *Bacteroides* are enriched in the ileum, where they may play a role in amino acid synthesis and the enterohepatic circulation of bile acids.^91^ Lastly, the right colon is characterized by an enrichment of *Klebsiella*, *Enterococcus* and *Lactobacillus*, which are likely engaged in fermentation processes and the production of short-chain fatty acids, while the left colon shows an enrichment of *Parabacteroides*, *Bifidobacterium* and *Dorea*, indicating their involvement in intestinal motility and bile acid metabolism.^91^ Of note, the biogeographical map also emphasizes the presence of bacterial translocation along the upper and lower gastrointestinal tract due to luminal flow conditions, as well as significant differences between mucosal and luminal samples.^91^ In general, the microbial diversity in the esophagus and stomach is markedly lower compared to the small intestine and colon.^91^

### Oral microbiome

The oral bacterial community is initially dominated by the genera *Streptococcus*, *Gemella*, *Granulicatella*, and *Veillonella* at birth, followed by an increase in *Lactobacillus* and *Fusobacterium*.^92^ *Staphylococcus* reaches its peak around 3 months of age before declining, making way for an increase in *Gemella*, *Granulicatella*, *Haemophilus*, and *Rothia* species.^93^ With the emergence of teeth, the oral microbiota transitions to include a greater abundance of *Fusobacteriota*, *Synergistetes*, *Tenericutes*, *Saccharibacteria (TM7)*, and *SR1* phyla as individuals progress into adulthood.^94–96^ Interestingly, re-analysis of raw 16S rRNA sequences of over 2000 saliva samples from 47 different studies identified 68 consistent core bacterial taxa.^97^ *Streptococcus oralis* subspecies dentisani is recognized as a potentially beneficial organism for oral health and is highly abundant across different oral niches in healthy humans.^97^ The *Neisseria* genus, dominant in the salivary microbiome, is associated with lipid metabolism pathways, suggesting a key role in regulating oral lipid-related metabolic processes.^97^ *Lautropia*, in conjunction with *Neisseria*, has been found to increase the abundance of certain metabolic pathways in Chinese samples, particularly those involved in lipid metabolism.^97^ In contrast, the *Prevotella* genus, which is less abundant in Western populations, may be linked to a reduction in specific metabolic pathways when compared to Chinese samples.^97^ The *Veillonella* genus, which is more abundant in Western populations, is linked to the ‘Flavone and flavonol biosynthesis’ pathway, whereas the *Atopobium* genus is observed to be less prevalent in the same demographic.^97^

### Skin microbiome

The skin bacterial community initially has a high presence of maternal vaginal *Lactobacillus spp*. at birth.^83^ By around weeks 4–5, the infant skin microbiota starts resembling that of adults but becomes more specific to different body areas during adolescence.^98^ Common genera include *Staphylococcus* and *Corynebacterium*, with *Pseudomonas*, *Enterobacter*, *Enterococcus*, *Proteus*, and *Klebsiella* at specific sites like the armpit or forearm.^99^ The skin bacteria can primarily be categorized into three major classes: sebaceous or oily including the face, chest, and back; moist such as the bend of the elbow, back of the knee, and groin; and dry like the volar forearm and palm. Sebaceous skin regions are notably enriched with *Propionibacterium acnes* and exhibit a variety of metabolic pathways that are pivotal to lipid metabolism and energy production, including glycolysis, ATP and GTP generation, and NADH dehydrogenase I.^100,101^ In contrast, dry skin regions are characterized by a distinct microbial composition that includes species such as *Corynebacterium* and *Staphylococcus epidermidis*, with a significant enrichment in citrate cycle modules that are likely adapted to the drier conditions of these areas.^100,102^ Moist skin regions are predominantly inhabited by fungi, particularly *Malassezia globosa* and *Malassezia restricta*, which thrive in the higher-humidity environment.^100,103^ The toenail region, which is unique compared to other skin types, houses a specific microbial community that is distinguished by its energy production components, including the conversion of oxaloacetate to fructose-6-phosphate, and the presence of ATPase and ATP synthase.^100^ Additionally, the skin’s microbiome as a whole serves as a reservoir for antibiotic resistance genes, displaying considerable variability among individuals and resistance types, with certain classes like MATE efflux pumps being highly host-specific.^100,103^

### Vaginal microbiome

Dominated by a single *Lactobacillus* species, the human vaginal microbiome is intriguingly different from that of other species, including primates.^104^ Currently, with a lack of reliable data on the neonatal vaginal microbiota, the developmental trajectory of the human vaginal microbiome remains incompletely understood. Before puberty, the vaginal microbiome exhibits high diversity, including *streptococci*, *enterococci* and *anaerobes*, possibly due to the thinning of vaginal epithelial cells and minimal glycogen deposition resulting from lower estrogen levels, which may not provide sufficient nutrition for Lactobacilli.^105^ However, in premenopausal women, the vaginal microbiome is dominated by one or a few *Lactobacillus* species, such as *L. crispatus*, *L. iners*, *L. jensenii* or *L. gasseri*, leading to reduced microbial diversity.^106^ This dominance is accompanied by an increase in oestrogen levels.^106^ During the menopause, declining estrogen levels result in decreased glycogen accumulation and reduced abundance of Lactobacilli, facilitating colonization by anaerobic bacteria associated with bacterial vaginosis and an increase in microbial diversity.^107–109^ Although approximately 25% of North American women have vaginal microbiomes that are not dominated by Lactobacilli, but instead consist of a mixture of anaerobic and aerobic bacteria, such as *Gardnerella*, *Prevotella*, *Atopobium*, *Sneathia*, *Megasphaera* and *Peptoniphilus*.^110^ *L. iners*, *L. crispatus* and *G. vaginalis* are the three most common bacterial species in the vaginal microbiota of nearly all ethnic groups of women studied to date.^111–116^ A recent notable study called “Isala”, conducted in Belgium, involved self-sampling (using citizen science methods) of 3,345 women.^117^ In this cohort of healthy individuals, *L. crispatus* was the most common taxonomic unit (43.2%), followed by *L. iners* (27.7%) and *G. vaginalis* (9.8%).^118^

### Respiratory microbiome

Encompassing the nasal cavity, sinuses, pharynx and supraglottic portion of the larynx, the upper respiratory tract has different microbial compositions in different regions.^119^ In particular, the nasal cavity and nasopharynx are dominated by *Moraxella*, *Staphylococcus*, *Corynebacterium*, *Haemophilus* and *Streptococcus* species, whereas the oropharynx hosts *Prevotella*, *Veillonella*, *Streptococcus*, *Leptotrichia*, *Rothia*, *Neisseria* and *Haemophilus* species.^120^ In neonates, the lower respiratory tract microbial community is dominated by either *Staphylococcus* or *Ureaplasma* species during the first weeks of life, correlating with mode of delivery; vaginal births enrich for *Ureaplasma*, whereas cesarean births enrich for *Staphylococcus*.^121^ In the first 2 postnatal months, lung microbiome diversifies to include oral commensals such as *Streptococcus*, *Prevotella*, *Porphyromonas* and *Veillonella*.^121^ However, the lower respiratory tract, comprising the trachea and lungs, maintain a low biomass that is essential for efficient gas exchange, supported by a rapid clearance system including immune actions such as mucociliary clearance and phagocytic activity of macrophages, as well as mechanisms like pulmonary surfactant and cough reflex.^122,123^

### Personalization and relative stability

The abundance and composition of microbial communities in different anatomical ecological niches can be influenced and disturbed by multiple host and external factors, resulting in highly personalized variations.^124^ However, they also exhibit relative stability and a certain degree of resilience.^125–127^ In a recent contribution from the iHMP, Zhou et al. reported on the dynamics of microbiomes at three body sites—oral, nasal, and skin—and in fecal samples from 86 individuals monitored longitudinally over six years.^128^ Their findings highlight the variable stability of the human microbiome across different individuals and anatomical sites, with fecal and oral microbiomes showing greater stability than those from the skin and nasal microbes.^128^ Moreover, microbiome characterized by high individual specificity are more stable over time, reflecting an enhanced regulation by the host.^128^ Microbes closely associated with human development can also serve as means to predict chronological age, with the skin microbiome offering the most accurate age predictions (mean error ± standard deviation of 3.8 ± 0.45 years), outperforming both the oral microbiome (4.5 ± 0.14 years) and the gut microbiome (11.5 ± 0.12 years).^129^ In summary, in reviewing the past deciphering efforts, we are progressing along the path of identifying common microbiota, then core microbiota, and finally individualized microbiota. At the same time, the accompanying exploration of microbiota functionality and perturbation mechanisms is approaching the possibility of regulation.

## Physiology and regulatory role of commensal microbes

### Digestion and microbiome-related products

Microbiome-related products encompass a range of substances, including microbiota-derived metabolites (MDM), microbiota-derived components (MDC), and microbiota-secreted proteins (MSP).^130^ Nestled in the complex ecosystem of the digestive tract, a thriving microbial community produces an extensive repertoire of metabolic enzymes (biogenic enzymes, glycosidases, and proteases), thereby enhancing the digestive and metabolic capabilities of the human body.^15,16^ For example, humans lack specific and efficient nitrate reductases^131^ which is essential to convert dietary nitrate into nitrite and Nitric oxide (NO) through the nitrate-nitrite-NO pathway.^132^ The oral cavity harbors nitrate-reducing bacteria, such as *Veillonella* and *Actinomyces*.^133^ The nitrate reductase Nar in oral bacteria is encoded by genes *narX*, *narG*, *narJ*, *narH*, *narY*, *narI*, and *narW*.^134^ Additionally, certain bacteria like *Rothia* possess nitrite reductase encoded by genes nirK and nirS, which further reduce nitrite to NO.^135^ NO has long been recognized as an endothelium-derived relaxing factor, functioning as a vasodilator and modulating vascular tone, blood pressure, and hemodynamics.^136,137^

**Fig. 2:**
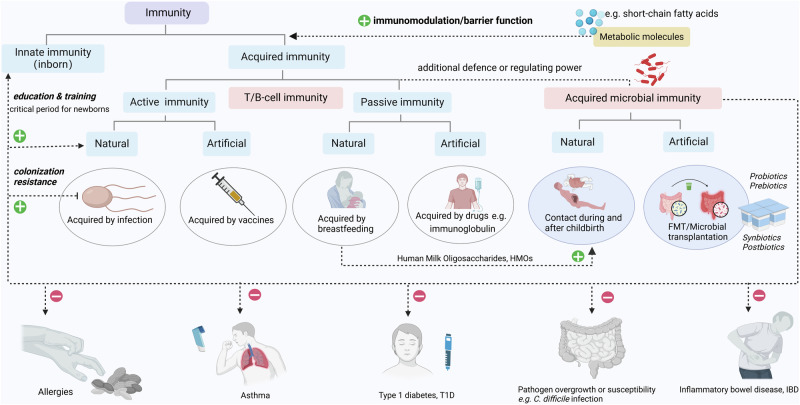
Acquired microbial immunity. The human immune consists of innate and acquired immunity, which is mainly carried out by T and B cells. The main strategies of adaptive immunity are active and passive immunization. In active immunity, natural immunity can be acquired by direct infection with the pathogen, while vaccination with the antigen is the artificial way. Passive immunization is mainly achieved by natural means, such as breastfeeding, or artificial means, such as immunoglobulin injections. Commensal microbiota described here can provide another form of acquired defence and regulating power against pathogens (commensal microbiota immunity). Correspondingly, maternal human milk oligosaccharides (HMOs), acquired through maternal reproductive transmission and exposure, can enhance the colonization of beneficial microbes under natural conditions. Under artificial conditions, fecal microbiota transplantation (FMT),^660–664^ probiotics,^665–669^ prebiotics,^670^ synbiotics^671,672^ and postbiotics^673–676^ can be used to acquire this immunity. Commensal microbiota immunity strengthens cellular barriers and regulates immune cells through metabolites such as short-chain fatty acids. They train and educate the immune system as a competitor while providing colonization resistance against foreign and established pathogenic microbes. The decline of commensal microbiota immunity increases the risk of skin and food allergies,^677^ asthma,^548^ type 1 diabetes (T1D),^678^ pathogenic overgrowth (such as *Clostridium difficile*),^667,679–687^ and susceptibility to inflammatory bowel disease (IBD)^555,688–695^ and other potential diseases^696,697^

Involved in the synthesis of vitamin K and most water-soluble B vitamins, these microorganisms actively contribute to the production of prothrombin and osteocalcin, thus influencing blood coagulation and bone metabolism.^26^ They also serve as essential cofactors and coenzymes that are central to various cellular metabolic pathways.^138^ Beyond their enzymatic contributions, gut-dwelling microbes orchestrate the assimilation and conversion of carbohydrates, proteins, and amino acids, yielding a range of essential products.^139,140^ These include short-chain fatty acids (SCFAs) as well as branched-chain amino acids (BCAAs), secondary bile acids (BAs), polyamines, lipids and an enigmatic realm known as “dark matter”.^26,138^ These remarkable entities have emerged as key participants in human tissue development, neural function, immune response (Fig. 2), metabolism, and behavioral regulation, revealing their profound impact on human well-being.^141^ It’s important to note that the digestive tract serves as the primary gateway for the body to actively absorb nutrients and substances from the external environment. As a result, the oral and gastrointestinal microbiomes play an integral role in digestion. Whereas, microbes in other sites of the body are primarily involved in physiological regulation through mechanisms such as colonization resistance, immune modulation and maternal transmission.

### Metabolic regulation

#### Short-chain fatty acid

Anaerobic bacteria ferment non-digestible substrates such as non-starch polysaccharides, resistant starch, and oligosaccharides, resulting in the production of SCFAs (mainly including acetate, propionate, and butyrate).^142^ The majority of these SCFAs are rapidly and almost completely absorbed by colon cells.^143^ Acetate enters the liver through the portal vein and is released into the peripheral tissues for cholesterol metabolism and fatty acid synthesis.^144^ Propionate is involved in gluconeogenesis regulation, inhibition of cholesterol synthesis, and interactions with intestinal fatty acid receptors to regulate satiety.^145–148^ Butyrate controls intestinal hormones, reduces appetite and food intake.^149^ Importantly, butyrate metabolism serves as a source of energy for 60–70% of the colon, maintaining a hypoxic state in the gut through β-oxidation, reducing intestinal inflammation and preserving mucosal integrity.^150,151^ Furthermore, butyrate has been shown to have beneficial effects in inhibiting colon cancer cell proliferation, differentiation, invasion, and inducing apoptosis.^152–154^

#### Secondary bile acids

The gut microbiota actively participates in the conversion of primary bile acids to secondary bile acids and plays a crucial role in the enterohepatic circulation of bile acids.^155^ It exerts regulatory control over glucose homeostasis, lipid metabolism, insulin signaling, and inflammation through the FXR and TGR5 receptors.^156^ Abnormalities in bile acid metabolism have been implicated in several diseases, including irritable bowel syndrome (IBS), colorectal cancer, neuroinflammation, and non-alcoholic fatty liver disease.^155–157^

#### Arg-Lys-His

The novel tripeptide Arg-Lys-His (RKH), synthesized by *Akkermansia muciniphila* (AKK), binds to TLR4 receptors and inhibits TLR4-mediated signaling pathways. This reduces sepsis-induced inflammatory cell activation and excessive cytokine release which protects against sepsis-related mortality and organ damage.^158^

#### Indole-3-propionic acid (IPA)

IPA was found to be decreased in a mouse model of autism spectrum disorder (ASD) leading to deficits in social interaction and cognitive memory.^159^ Mechanistically, IPA restores inhibitory synaptic transmission in the hippocampal region by activating the ERK1 signaling pathway, which is encoded by the MAPK3 gene located within the 16p11.2 chromosomal region.^159^

#### Homovanillic acid (HVA)

*Bifidobacterium longum* (*B. longum*) produces HVA, a metabolite that modulates synaptic integrity by inhibiting excessive autophagy.^160^ This mechanism reduces the degradation of microtubule-associated protein 1 light chain 3 (LC3) and the protein SQSTM1/p62, safeguarding the synaptic vesicle membrane of hippocampal neurons and contributing to depression alleviation.^160^ *Roseburia intestinalis* (*R. intestinalis*), athough not a HVA producer, facilitates the growth of *B. longum*, indirectly enhancing HVA synthesis.^160^

Also, the induction of specific Helper T cell 17 (Th17) expression by skin commensal microbiota is associated with transcriptional programs relevant to skin-neuronal interactions and repair.^161^ Following skin injury, Th17 cells upregulate IL-17A, which binds to IL-17A receptors upregulated on damaged nerves, promoting axonal growth and local nerve regeneration.^161^ Disruption of the pulmonary microbiota significantly influences susceptibility to autoimmune diseases of the central nervous system.^162^ Augmenting microbial populations capable of producing lipopolysaccharide (LPS) can enhance endogenous immune factors in brain microglial cells, thus modulating neuroimmune responses in the brain.^162^

### Epigenetic modulation

Epigenetic changes represent reversible modifications in gene expression regulation and heritable traits that occur without permanent changes to the DNA sequence, and can even be transmitted to offspring through sexual reproduction.^163–166^ It has duly been established that the human microbiota can extensively influence the expression of the human genome through mechanisms such as DNA methylation, histone modification, non-coding RNA and chromatin remodeling, leading to a broad range of physiological impacts.^167^ Early-life epigenetic crosstalk significantly impacts the development of adult tissues.^168^

#### DNA methylation

DNA methylation at CpG islands can recruit methyl-CpG-binding proteins, which alter chromatin conformation, leading to chromatin condensation that prevents the binding of transcription factors and RNA polymerase, thus inhibiting the expression of specific genes.^169^ Metabolites produced by microbial communities can serve as substrates and/or co-factors in these reactions/interactions. For example, folate can metabolically generate S-adenosylmethionine (SAM), which becomes a substrate for DNA and histone methylation.^170^ Microbiota can adjust DNA methylation in mice intestinal epithelial cells, affecting the expression of 824 upregulated and 358 downregulated genes.^168^ TET2/3 enzymes are key in this process, facilitating the conversion of 5-methylcytosine to 5-hydroxymethylcytosine, which promotes demethylation and the activation of genes essential for intestinal homeostasis.^168^

#### Histone modification

Histones are the fundamental proteins that make up chromatin, tightly binding with DNA to form nucleosome structures, which then coil and fold into complex chromatin.^171^ Modifications of histones, like acetylation and methylation, regulate the condensation of chromatin, thus regulating gene expression.^172^ For example, microbiota-derived butyrate can inhibit the activity of histone deacetylases (HDACs), leading to increased acetylation of histone H3 at the Foxp3 gene locus.^173^ This acetylation enhances the expression of the Foxp3 gene, facilitating the differentiation of colonic regulatory T cells (Treg).^173^ Gut microbiota can also regulates intestinal epithelial gene expression by suppressing Hepatocyte Nuclear Factor 4 Alpha (HNF4A) through reduced DNA binding and altered histone modifications such as Histone 3 Lysine 4 monomethylation (H3K4me1) and Histone 3 Lysine 27 acetylation (H3K27ac), which could be linked to the pathogenesis of Inflammatory Bowel Disease (IBD).^174^

#### Non-coding RNA

Non-coding RNAs (ncRNAs), including microRNAs (miRNAs), circular RNAs (circRNAs) and long non-coding RNAs (lncRNAs), regulate host gene expression through various mechanisms.^175^ MiRNAs modulate protein synthesis within host cells by binding to complementary sequences on messenger RNAs (mRNAs), leading to mRNA degradation or translational repression, while circRNAs function as “sponges” for miRNAs, sequestering them to modulate their activity.^176^ Microbiota can influences the expression of the miR-181 in white adipose tissue of mice through the production of tryptophan-derived metabolites, such as indole and indole-3-carboxylic acid (I3CA).^177^ This mechanism leads to a decrease in miR-181 expression within white adipocytes, which in turn stimulates increased energy expenditure and enhanced insulin sensitivity, counteracting the development of diet-induced obesity and insulin resistance.^177^ LncRNAs regulate genes through multiple mechanisms: they can organize protein complexes on DNA, direct proteins to gene sites, and change the epigenetic marks that control gene activity.^178^ They also interact with transcription factors, influence mRNA splicing and produce regulatory RNAs like miRNAs.^178^ Significant differences in lncRNA expression occur when germ-free mice are re-colonized with distinct microbial types, such as complex mouse microbiota, *E. coli* or *E. coli* expressing bile salt hydrolase (EC-BSH), with only a few lncRNAs showing overlap and most being type-specific.^179^

### Immunomodulation

Commensal microorganisms play a fundamental role in the education and maintenance of immune homeostasis. In the past few decades, there has been a notable increase in the incidence of allergic diseases, such as asthma, atopic dermatitis, and food allergies, as well as autoimmune diseases like type 1 diabetes (T1D) in industrialized countries.^49,180,181^ Interestingly, individuals who migrate from countries with low incidence rates of these conditions to those with higher rates, and do so before a certain age threshold, tend to adopt the disease prevalence of their host country. For instance, research indicates that children who move to countries with higher incidences of allergic asthma before the age of 5 are more likely to develop asthma at rates similar to those of the host country’s population.^182^ Similarly, the risk of type 1 diabetes has been observed to increase in migrants who move before adolescence, with studies suggesting a critical age threshold around 15 years for the development of multiple sclerosis.^183–186^ In 1989, David Strachan proposed the novel concept that infections could serve as a preventative measure against the development of allergic diseases.^187^ Building on this idea, in 2000, he formally introduced the term “hygiene hypothesis” to describe the observed correlation between a lower incidence of infectious diseases in early life and the rising prevalence of allergic conditions,^188^ which exerted a profound influence on public health.^189,190^ Subsequently, Rook et al. as well as Noverr and Huffnagle, further emphasized the “Old Friends Hypothesis” or “Microflora Hypothesis,” highlighting the importance of microorganisms in achieving immune homeostasis in the human body.^191,192^ It is recognized that the immune system development of the individual involves critical developmental periods.^193^ Early exposure to a diverse range of microbes is essential for the proper development of the immune system, with the activation of immune regulatory pathways, particularly through Toll-like receptors (TLRs), fostering a balanced immune response. This process is thought to promote the generation of regulatory T cells (Treg), which produce anti-inflammatory cytokines like IL-10 and TGF-β, thus suppressing excessive immune reactions and potentially reducing the risk of developing allergic and autoimmune diseases. The protective effects of commensals are also suggested to involve antigenic competition and the modulation of inflammatory responses, possibly through mechanisms like TLR desensitization.^193^

### Colonization resistance

Microbial communities that inhabit human mucosal surfaces or skin are capable of preventing the colonization of pathogens and overgrowth of indigenous pathogens, known as “colonization resistance” (Fig. 2).^194,195^ This phenomenon was first discovered by Bohnhoff and Miller in 1967 when they observed increased susceptibility of mice to *Salmonella* infection following treatment with streptomycin.^196^ This antibiotic-related susceptibility explains the widespread harm caused by antibiotic abuse in recent years,^197,198^ as commensal microbial colonization appears to provide the body with an additional defense mechanism. They compete with pathogens through various mechanisms for the specific nutritional and physicochemical environment of the human body, ultimately leaving newcomers unable to secure adequate nutrition and space for survival and reproduction.^199^

The way microbes protect their own territory may indirectly protect the human body. For example, *S. salivarius* TOVE-R strain is effective against virulent *streptococci* like *S. mutans*, *S. sobrinus* and *S. pyogenes*, which are associated with tooth decay, pharyngitis, and periodontitis.^200^ Its bacteriocin (a type of heterogenous peptide) inhibits *S. pyogenes* and *S. pneumoniae*^200^ and can also modulates immune responses by inhibiting inflammatory pathways activated by these pathogens.^201^ Coagulase-negative *staphylococci* (CoNS), which are typically present in the skin and nasal cavity, secrete bacteriocins that reduce the colonization of pathogenic *S. aureus*.^202^ The commensal bacterium *S. epidermidis*, through the secretion of serine protease Esp, can degrade and inhibit the biofilm of *S. aureus*, reducing its virulence.^203^ Torres Salazar et al. have elucidated that *S. epidermidis* can also produces a novel, rapidly degrading broad-spectrum antibacterial agent termed epifadin, which demonstrates efficacy in mitigating nasal colonization by *S. aureus*.^204^ Analyzing 2229 bacterial genomes from the Human Microbiome Project, sourced from diverse body sites such as skin, gastrointestinal tract, urogenital tract, mouth, and trachea, researchers identified gene clusters encoding for lanthipeptides and lasso peptides.^205^ These clusters direct the synthesis of peptides that, through unique post-translational modifications, give rise to novel compounds exhibiting antimicrobial activity.^205^

In addition to bacteriocin and enzyme secretion, common metabolites such as SCFAs can inhibit the growth of pathogenic *Escherichia coli*,^206^ *Clostridium difficile*,^207^ and *Salmonella*^208^ in the gut. Moreover, secondary bile acids have been shown to inhibit many Gram-positive bacteria, including *C. difficile*.^209^

Nevertheless, microbes can also develop resistance to being colonized by other microbes through direct physical interactions. Contact-dependent inhibition systems have been discovered in microorganisms such as *E. coli* and *Pseudomonas aeruginosa*, which can inhibit neighboring microbes by targeting their receptor proteins.^210,211^ Many Gram-negative bacteria, such as *P. aeruginosa* and *Burkholderia spp*., can use type VI secretion systems (T6SS), a multi-protein complex that punctures nearby microbes and injects toxic proteins.^212–215^ Interestingly, microbes that occupy niches also participate in the niche modification. A commonly cited example is Lactobacilli, a resident of the vaginal microbiota, which lowers the pH of the vagina, thereby reducing the colonization of pathogenic bacteria that can thrive in neutral environment.^110,216,217^ The mechanisms of microbial colonization resistance may shift depending on the environment where they are situated. In germ-free or antibiotic-treated mice, e.g. *Klebsiella oxytoca* inhibits the growth of *S. Typhimurium* by producing toxins such as tilimycin.^218^ Whereas, in mice with a complex microbiota, *K. oxytoca* competes with *S. Typhimurium* for survival resources by utilizing specific carbohydrates like dulcitol.^218^

In general, if the exposed areas of the human body are inevitably colonized by external microbes, perhaps the best strategy would be to use controlled microbes as the first line of defense in external immunity, thus minimizing the disruption of internal immunity. Salvarsan, the first antibiotic in 1910, heralded a medical revolution.^219^ The subsequent discovery of penicillin in 1928 propelled us into the golden age of antibiotics, pivotal in saving lives and advancing civilization.^220^ However, the widespread utilization of antimicrobial drugs has resulted in a rising incidence of infections caused by antimicrobial resistance (AMR) globally, leaving us in a quandary with diminishing treatment alternatives.^221^ In 2019 alone, an estimated 4.95 million deaths were linked to bacterial AMR, with 1.27 million deaths directly attributable to it.^197^ By exploiting the immunomodulatory properties of microorganisms and implementing colonization resistance strategies, it is hoped that dependence on antimicrobial drugs will be reduced and a promising path will be opened in the present predicament.

### Regulation and transmission of parental microbiota

The maternal gut microbiota produces SCFAs that can enter the embryo through the mother bloodstream.^222^ SCFAs act on the GPR41 receptor in the sympathetic nervous system of the fetus and GPR43 receptor, which is highly expressed in intestinal epithelial cells and pancreatic beta cells, promoting the development of prenatal metabolic system in neurons, enteroendocrine cells and beta cells.^222^ This reduces the risk of offspring developing metabolic syndrome.^222^ The normal development of the fetal brain and nervous system in mice is also influenced by maternal microbiota and its metabolites.^223^ Treatment with antibiotics or germ-free pregnancy in mice can lead to defective growth of embryonic hypothalamic axon and a decrease in tactile sensitivity in adulthood.^223^ Although research in humans is limited, a recent follow-up study of 860 children found an association between the maternal gut microbiota during pregnancy and neurodevelopment in the first year after birth.^224^ In addition, the presence of *Clostridia* in the maternal gut microbiota is associated with high fine motor skills in children.^224^ Microbial colonization also drives innate immune development in offspring, increasing certain innate lymphocytes and monocytes while causing widespread changes in the gene expression profile of the intestinal epithelial mucosa, better preparation for colonization by postnatal microbes and prevention of microbial invasion.^225^ In conclusion, maternal microbiota during pregnancy may participate in the regulation of fetal endocrine, neural and immune development through multiple mechanisms. However, vaginal and fecal contact, as well as skin-to-skin contact and later breastfeeding, provide more than half of the initial microbial colonization for infants.^226^ While the transmissibility of different microbial species can vary depending on the mode and place of delivery, species such as *Bifidobacterium* have demonstrated consistent vertical transmission regardless of the delivery environment.^227^ In addition to supporting the development of vital organs^228^ and providing defense against harmful bacteria in the infant’s gut,^229,230^ maternal milk provides probiotics^231^ and human milk oligosaccharides (HMOs) that facilitate the metabolism and colonization of beneficial bacteria like *Bifidobacterium*.^232^ Given the current limitations of commercial formulas in fully replicating human milk,^233,234^ it is critical to prioritize breastfeeding as the primary feeding method.^235–238^ Fathers are also a consistent source of infant strains and their cumulative contribution equals that of mothers after one year.^239^ Recent research suggests that dysbiosis in the gut microbiota of male mice prior to conception can affect testicular function and sperm quality, as well as lead to compromised placental function in female mice, thereby increasing the risk of offspring with low birth weight, severe growth restriction and early mortality.^240^ These findings underscore the potential value of microbiota in guiding reproductive health.

## Germ-free syndrome

David, also known as the “Bubble Boy”, was born in 1971 with severe combined immune deficiency syndrome (SCID) and lived his entire life in a sterile isolation unit.^241^ Unfortunately, he died at the age of 12 years due to severe infection.^241^ Although it was rarest of the cases, due to the lack of advanced technologies available today, incomprehensive health evaluations, particularly the lack of anatomical data, prevent us from fully understanding this unique germ-free human individual. More importantly, individuals with underlying diseases are also not an ideal subject for research. Since 1940, the germ-free (GF) animal model has gradually become a cornerstone of microbiological research, meticulously cultivated in sterile environments through cesarean section, where pups are extracted from sterile mothers and reared by surrogates—or by artificial rearing, which nurtures cesarean-born pups with formula milk in an aseptic setting, ensuring their lifelong freedom from microorganisms.^242–245^ The progressive commercialization of GF mice/rat in laboratories has elucidated the phenotypes of these animals, shedding light on the intricate interplay between the microbiome and the health of organisms.

### Systemic somatic growth and development

At the age of 8 weeks, GF mice exhibited a 14.5% reduction in weight and were 4% shorter in stature compared to their conventionally raised (CONV-R) peers.^246^ The disparity in weight was not a result of increased adiposity, as both groups demonstrated equivalent adipose tissue and serum leptin levels.^246^ After weaning, GF mice consumed food at a rate comparable to their body weight as CONV-R mice did, yet differences in nutrient absorption and utilization efficiency may account for growth discrepancies.^246^ Despite elevated early levels of growth hormone in GF mice, this did not enhance insulin-like growth factor–1 (IGF-1) and insulin-like growth factor binding protein 3 (IGFBP-3) levels, indicating growth hormone resistance.^246^ Most organs and tissues, including the heart, liver, spleen, thymus, thyroid, skin and intestine, are reduced in mass or size.^243^

### Cardiovascular system

The cardiac output is approximately 30% lower in GF rats compared to conventional controls, accompanied by a mild phenomenon of hemoconcentration which result in reduced blood supply to peripheral organs.^247,248^ Under normal oxygen conditions, the transcriptomic changes in the hearts of GF mice revealed 117 differentially expressed genes, with 73 genes upregulated and 44 genes downregulated.^249^ These changes implicate key biological processes such as cardiac function, cell proliferation, transcriptional regulation, and immune response.^249^ For instance, the upregulation of *Amd1* may foster cell proliferation, while the downregulation of *Cacna1d* could affect the electrophysiological properties of the heart.^249^ These alterations might have short-term beneficial impacts on cardiac health but could also pose long-term risks for disease development. In contrast, under conditions of intermittent hypoxia and hypercapnia (IHH), which simulate obstructive sleep apnea syndrome, CONV-R mice exhibited 192 changes in gene expression, predominantly related to cardiac cell death and cardiac hypertrophy.^249^ Genes such as *Bcl2l1*, *Cryab*, and *Gsn* showed regulatory changes that could influence the heart’s response to stress.^249^ Whereas, GF mice displayed 161 gene expression changes, more closely associated with regulators of cardiac hypertrophy, including the downregulation of genes like *Ace*, *Ankrd1*, and *Aplnr*, and the upregulation of genes such as *Cdkn1a*, *Fhl2*, *Rgs2*, and *Stat3*.^249^ During fasting, GF mice showed a significant decrease in heart weight, linked to a notable alteration in the pathways of cardiac metabolism.^250^ With the lack of microbiota, there is a reduction in the generation of hepatic ketone bodies, causing the hearts of GF mice to shift their dependence towards glucose as the principal energy substrate to maintain performance.^250^ The absence of the microbiota also adversely impacts vascular integrity, with these impacts being sexually dimorphic.^247^ Regardless of sex, GF mice exhibit reduced vascular contractility; however, male mice display increased vascular stiffness and inward hypertrophic remodeling, indicative of chronic blood flow reduction, while female mice exhibit outward hypertrophic remodeling, potentially associated with vascular aging.^247^

### Respiratory system

GF mice displayed a 24% reduction in both nasal paranasal sinus mucosa and epithelial layer thickness, coupled with a 45% increase in collagen content and a 50% decrease in goblet cell count.^251^ Additionally, the nasal-associated lymphoid tissue (NALT) area in GF mice was reduced by 30%, indicating a compromised local immune response.^251^ Their lungs are characterized by a reduced number of alveoli, an enlargement in alveolar dimensions, and a decrease in mucus secretion.^252,253^ In room air, GF and CONV-R mice exhibit similar lung development and function, along with comparable pulmonary vascularization in both normoxia and hyperoxia; however, GF mice demonstrate reduced hyperoxia-induced lung injury and improved lung function compared to CONV-R mice.^254^ This is because under hyperoxia, the pulmonary microbiota shifts favoring oxygen-resistant species such as *S. aureus*, with this alteration preceding and correlating with the severity of lung inflammation.^255^

### Digestive system

In terms of intestinal morphology, GF mice exhibit a reduction in both the total mass of the intestine and the overall surface area of the small intestine.^256^ The villi of the small intestine are slender and uniform, with shorter villi in the ileum and longer villi in the duodenum.^257^ The rate of cell renewal in the crypts of the small intestine is slower.^258,259^ A prominent feature of GF rats is the enlargement of their cecum, a condition that results from the accumulation of mucopolysaccharides, digestive enzymes, and water within the intestinal lumen.^260^ During periods of fasting, the cecum of GF rats expands considerably, and the majority of the proteins and carbohydrates within its contents originate from within the body, indicating that the small intestine’s ability to effectively break down and assimilate these materials is compromised.^261^ Regarding intestinal motility, the intrinsic primary afferent neurons (IPANs) in the enteric nervous system of GF mice demonstrate reduced baseline excitability, as indicated by an enhanced slow afterhyperpolarization (sAHP), leading to an extended refractory period following the initial neuronal firing.^262,263^ This disruption potentially affects the rhythmicity and coordination of gut movements, resulting in irregularities such as abnormal transit rates—either slowed or accelerated—and irregular peristalsis.^262^ Furthermore, GF mice showed a diminished response to the IKCa channel blocker TRAM-34, a drug that typically modulates gut motility.^262^ Their increase in muscular tissue in the cecum, characterized by elongated and hypertrophied muscle cells, which also leads to an extended transit time through the intestines.^264^ Physiologically, there is a decrease in osmolarity within the small intestine, while the oxygen tension and electrical potential are elevated.^257^ Functionally, GF mice showed enhanced absorption of vitamins and minerals, with alterations in the uptake of other ingested substances.^265^ There is also a change in the enzymatic content of the feces, with increased levels of trypsin, chymotrypsin, and invertase.^266^ The feces of GF mice have a higher content of mucin (mucoproteins and mucopolysaccharides), and there is a reduction in fatty acids within the intestinal content, with a predominance of excreted unsaturated fats.^257^ While the inability to synthesize certain vitamins, GF mice/rats require additional dietary supplementation of vitamins like K and B.^267–269^ However, these mice also experience an impact on fluid balance, evidenced by an increased intake of water.^265^

### Kidney function

The detrimental aspects of kidney health in GF mice are characterized by a significant increase in the expression levels of purine-metabolizing enzymes, such as xanthine dehydrogenase (XDH), which leads to higher urinary excretion of purine metabolites.^270^ Particularly, the production of 2,8-dihydroxyadenine (2,8-DHA), a nephrotoxic byproduct, is elevated, exacerbating adenine-induced kidney damage.^270^ Moreover, the fecal purine metabolite profile in GF mice is substantially altered, with higher levels of guanosine, inosine, xanthine, and urate, and lower levels of guanosine monophosphate (GMP), adenosine monophosphate (AMP), guanine, adenosine, adenine and hypoxanthine compared to mice with a normal microbiota.^270^ These alterations in purine metabolism and the presence of toxic metabolites contribute to the increased vulnerability of GF mice to kidney injury.

### Internal metabolism

The thyroid gland of GF mice, which is responsible for iodine uptake and storage, showed a reduced ability to concentrate inorganic iodine and a decrease in basal metabolic rate.^271^ During the light phase, when resting and fasting, GF mice have a lower respiratory exchange ratio, signifying a reliance on fat oxidation for energy.^272^ Low liver glycogen levels at the end of the light phase may suggest a more rapid depletion of hepatic glycogen in these mice.^272^ Conversely, during the dark phase, when they are active and consuming food, their metabolism favors glucose as the main energy source.^272^ The liver’s circadian gene expression is significantly altered, with a notable reduction in sex-based differences, which correlates with shifts in sex hormone levels and growth hormone secretion patterns.^273^ The diminished levels of ghrelin in these mice are rectified by exogenous administration, which normalizes gene expression and metabolism, underscoring the microbiota’s role in hormonal and metabolic regulation.^273^ Additionally, GF mice showed impaired liver regeneration and diminished conversion of cholesterol and bile acids, suggesting a reduced capacity for metabolizing these lipids into necessary compounds or excretory products.^274^ The microbiota’s influence on liver regeneration may primarily mediated through the regulation of bile acid and short-chain fatty acid metabolism, the activation of immune cells and cytokines including IL-6 and TNF-α, and the modulation of immune responses via metabolic byproducts such as LPS.^275^

### Immune system

GF mice have a range of immune defects such as reduced in size and cellular content of thymus, an important immune organ, decreased circulating immune cell numbers (T cells, B cells and white blood cells) and antibodies.^243,276,277^ These animals showed significant underdevelopment of the gut-associated lymphoid tissue (GALT), including reduced volume and cellularity of the Payer’s patches and mesenteric lymph nodes.^278^ Additionally, they have a decreased number of CD8+ T cells within the intestinal epithelial lymphocytes (IELs),^279^ and a proportional decrease in CD4+ T cells, with notable differences in the quantity and distribution of Th17 cells.^280^ At the molecular level, there is a decrease in the expression of antimicrobial peptides in Paneth cells,^281^ a reduction in secretory IgA produced by B cells,^282^ and lowered expression of MHC II molecules and TLR 9 in intestinal epithelial cells, along with a decrease in IL-25 levels, which may affect microbial recognition and immune response.^283,284^ GF mice also have a reduced resistance to various pathogens, demonstrating decreased immune resistance and increased mortality upon infection.^285^ Moreover, immune abnormalities may also lead to Sjögren-Like Lacrimal Keratoconjunctivitis.^286^

### Neurological and behavioral alterations

GF mice display higher blood-brain barrier (BBB) permeability from embryonic day E16.5 through E18.5, a condition that persists into adulthood.^287^ In these mice, the expression of key tight junction proteins, occludin and claudin-5, is lower, leading to a weakened BBB that allows more substances to enter the brain from the bloodstream which can affect brain development.^287^ Throughout their development, GF mice demonstrate pronounced differences in brain structure, maturation and behavioral performance.^288^ Compared with GF mice, CONV-R mice showed greater development in gray and white matter volume, fractional anisotropy and myelination, leading to enhanced spatial learning and memory, along with reduced anxiety and improved social novelty recognition.^288^ Notable disparities in the brain architecture of GF mice were observed, particularly impacting the amygdala and hippocampus.^289^ The amygdala showed a pronounced enlargement, with both aspiny interneurons and pyramidal neurons exhibiting dendritic hypertrophy.^289^ These neurons were characterized by an elevated count of dendritic spines, featuring an array of slender, stubby, and mushroom-shaped profiles.^289^ In stark contrast, the ventral hippocampus of GF mice presented with pyramidal neurons that were not only shorter but also displayed reduced branching, alongside a diminished presence of stubby and mushroom spines.^289^ Moreover, while the dentate granule cells in GF mice exhibited a decreased complexity in branching, the overall spine density was found to be consistent with that of conventionally colonized counterparts.^289^ These extensive modifications may potentially contributing to the observed stress responsivity, anxiety-like behaviors and deficits in social cognition in GF mice.^290^ Although GF mice showed reduced social activity and elevated cortisol levels after social interactions, colonization with *Fecalibacterium* improves social deficits and normalizes cortisol levels after social interaction.^291^ From the notable reductions in nerve fiber diameter and an increase in hypermyelination, peripheral nerves and dorsal root ganglia demonstrated a significant delay in the development leading to skeletal muscle atrophy and impaired development as well as maturation of neuromuscular junctions, highlighting the crucial role of the microbiota in the proper growth and functionality of the somatic peripheral nervous system.^292^ In particular, the axons of intestinal wall nerves undergo significant degeneration with advancing age.^293^ Transplanting microbiota from CONV-R into GF mice alters the neural anatomy of the enteric nervous system and improves intestinal transit, facilitated by microbiota-regulated 5-HT release.^294^ The establishment of the mucosal glial cell network is a postnatal process, where they form a population that is continuously renewed and essential for the preservation of gut homeostasis.^295^ The gut microbiota plays a pivotal role in not only guiding the development of this network but also in the ongoing regulation and sustenance of these mucosal cells.^295^

### Reproductive system

Typically, GF mice showed irregular estrous cycles, particularly due to the prolongation of the luteal or metestrus phases, which leads to a reduced frequency of the entire cycle.^296^ This irregularity may be associated with fluctuations in the levels of sex hormones. Moreover, GF mice generally have a lower reproductive capacity, which may manifest as lower mating rates, implantation rates, and litter sizes.^296^ Male mice showed delayed lumen formation in the seminiferous tubules and increased Blood-Testis Barrier (BTB) permeability at postnatal day 16, which correlated with reduced expression of intercellular adhesion molecules such as occludin, ZO-2 and E-cadherin.^297^ Additionally, male mice had lower serum levels of gonadotropins (LH and FSH) compared to CONV-R mice, and their testicular testosterone levels were also lower than those peers.^297^ In terms of sperm vitality, male mice may have lower sperm motility compared to CONV-R mice, potentially impacting fertilization ability and reproductive success.^296^ Notably, when GF mice are accidentally exposed to certain bacteria, such as *B. distasonis* and *C. perfringens*, their reproductive capacity significantly improves which is evidenced by the normalization of estrous cycles, increased mating and implantation rates, and enhanced sperm motility.^296^ Exposure to *C. butyricum*, capable of producing high levels of butyrate, restored the integrity of the BTB and normalized the levels of cell adhesion proteins in GF male mice.^297^

### Skeletal system

GF mice showed increased bone mass and altered bone matrix properties, characterized by reduced bone resorption, enhanced trabecular microarchitecture, elevated tissue strength, and increased bone mineral density, along with decreased whole-bone strength compared to CONV-R mice.^298^ The elevated bone mass evident in increased bone mineral density and cortical thickness, is primarily due to reduced activation of the NOD1 and NOD2 signaling pathways.^299^ The reduction leads to decreased expression of inflammatory cytokines like TNFα and the osteoclastogenic factor RANKL, resulting in fewer osteoclasts and consequently less bone resorption.^299^ Additionally, the collagen structure of the bones in GF mice is altered, yet they do not exhibit reduced fracture toughness.^298^ These changes are accompanied by sexual dimorphisms, particularly in bone tissue metabolism, with male GF mice showing an enhanced signature of amino acid metabolism, while female GF mice display an increased signature of lipid metabolism.^298^ Male rats born germ-free exhibit a significant acceleration in bone growth and changes in bone marrow cellular content following the reconstitution of the gut microbiota. Specifically, after the introduction of gut microbiota, these GF rats rapidly increased the bone mass of both cortical and trabecular bones, enhanced the bone tissue mineral density and improved the proliferation and hypertrophy of growth plate chondrocytes, leading to an increase in tibial length.^300^ In addition, there was an increase in the number of small-sized adipocytes and a decrease in the number of megakaryocytes in the bone marrow, indicating that the microbiota not only affects bone mass but may also regulate the bone marrow environment.^300^ The increase in short-chain fatty acids, particularly butyrate, may boost liver production of IGF-1, thus promoting bone growth through increased circulating IGF-1 levels.^300^

### Musculature

Various types of skeletal muscles in GF mice, including the tibialis anterior (TA), gastrocnemius, soleus, extensor digitorum longus (EDL) and quadriceps, exhibited significant abnormal phenotypes.^301^ Overall, these phenotypes encompassed reduced muscle mass, muscle fiber atrophy, mitochondrial dysfunction, and impaired neuromuscular junction (NMJ) function.^301^ Specifically, muscle atrophy was associated with upregulated expression of muscle growth inhibitory genes *Atrogin-1* and *Murf-1*, while the decrease in muscle quality and strength correlated with downregulated expression of IGF-1.^301^ Also, depletion of the microbiota results in elevated levels of the FXR antagonist TbMCA, which suppresses the FXR-FGF15 pathway and lowers FGF15, finally reduces ERK signaling necessary for muscle protein synthesis.^302^ The expression of muscle-specific transcription factors MyoD and Myogenin was diminished, affecting the differentiation and regenerative capacity of muscle cells.^301^ Mitochondrial dysfunction was reflected in the reduced mitochondrial DNA content and SDH activity, linked to decreased expression of mitochondrial biogenesis-related genes such as *Pgc1α* and *Tfam*.^301^ NMJ impairment was related to reduced expression of *Rapsyn* and *Lrp4*, alongside lowered serum choline levels, affecting the synthesis and neurotransmission of acetylcholine.^301^ Additionally, amino acid metabolism changes in the muscles of GF mice were observed, with increased levels of glycine and alanine, potentially connected to increased expression of the *Alt* gene.^301^ Decreased expression of glycolytic genes like *Pfk*, *Pk*, *Ldh* and *Pdh* impacted energy production.^301^ Increased glycogen accumulation in the quadriceps may indicate impaired glycogen metabolism.^301^ These integrated genetic and metabolic changes led to poor performance in muscle strength tests for GF mice.^301^ Interestingly, transplanting the gut microbiota from pigs into GF mice replicated the muscle phenotype of the donor pigs, including higher body fat mass, a greater proportion of slow-contracting fibers, reduced fiber size, lower percentage of fast IIb fibers and enhanced fat production in the gastrocnemius muscle.^303^

### Adipose tissues

GF mice have a lower percentage of body fat, despite the increased food intake^304^ and the elimination of sex-based differences in adiposity.^305^ They also show a reduction in adipocyte size marked by an increased quantity of smaller adipocytes coupled with a diminished presence of larger ones.^306^ The inguinal subcutaneous adipose tissue (ingSAT) and perigonadal visceral adipose tissue (pgVAT) regions exhibit browning features.^306^ Within the white adipose tissue, there is an observable infiltration of eosinophils and M2-type macrophages, which are implicated in the browning process of the adipose tissue.^306^ A reduction in lactate levels alongside an elevation in (D)-3-hydroxybutyrate levels within their brown adipose tissue (BAT) suggests an upregulated fatty acid oxidation pathway.^305^ It is known that they show resistance to diet-induced obesity through following mechanisms: increased levels of the fasting-induced adipose factor (Fiaf), which activates peroxisome proliferator-activated receptor gamma coactivator 1-alpha (Pgc-1α) to enhance fatty acid oxidation, and heightened AMPK activity, which is crucial for energy balance and metabolism.^307^ They may compensate for the impaired storage and utilization of glucose in skeletal muscle by increasing the lipolysis in adipose tissue and promoting the browning of adipose tissue, thereby meeting their energy demands.^308^ However, this compensatory mechanism might also limit their immediate fuel supply during exercise, leading to a decrease in exercise capacity.^308^ In addition, the levels of very-low-density lipoprotein (VLDL) are decreased while high-density lipoprotein (HDL) levels remain unaffected.^305^

### Skin

GF mice display reduced stratum corneum complexity, elevated transepidermal water loss and delayed healing post-injury, indicative of a compromised skin barrier.^309^ Decreased corneodesmosomes and downregulated genes crucial for keratinization and barrier integrity are observed.^309^ Notably, the aryl hydrocarbon receptor signaling pathway, key for skin homeostasis, shows reduced activity.^309^ Skin bacteria can enhance the production of inflammatory cytokine IL-1β, which in turn activates the IL-1 receptor and myeloid differentiation primary response 88 (MyD88) signaling pathway within keratinocytes.^310^ This activation is diminished in GF mice, which may partially explaining their reduced capacity for skin regeneration.^310^

### Longevity and death

Due to lack of potential infection by pathogens, GF mice have an extended average lifespan of 88.9 weeks, outliving CONV-R mice, which average 75.9 weeks.^311^ But under a restricted diet, equivalent to 80% of their usual intake, CONV-R mice significantly boost their lifespan to 117.5 weeks, while GF mice only increase to 109.6 weeks, showing that dietary restriction powerfully extends life, particularly in CONV-R mice.^311^ The deaths of GF mice are typically associated with gastrointestinal dysfunction, including intestinal atonia, an abnormally enlarged cecum (with the average weight of the cecum being approximately 15 times that of CONV-R mice at the time of death), intestinal volvulus, liver abnormalities, and degeneration of the kidneys.^312^ In contrast, the causes of death in conventional mice are more diverse, encompassing respiratory infections such as pneumonia, circulatory failure, intestinal volvulus, intestinal spasms, inflammations of the genital tract, peritonitis, and ear infections.^312^

Altogether, these findings demonstrate that the humans and other animals without microbiota are abnormal with severe deformities. Thus, to better characterize the functional dependence of animals on microbiota, the collective set of abnormal symptoms can be referred as “germ-free syndrome”. While we do not inhabit a sterile world,^313^ the progressive loss of microbes during infancy and adulthood, along with the cumulative effects across generations, may gradually propel humanity towards a state resembling germ-free syndrome.^314–318^ In this context, germ free, or more precisely, the absence of core microbiota, assumes clinical significance as it signifies a shift in the paradigm of microbial influence on disease—from focusing on the presence of pathogenic microbes to contemplating the consequences of a lack of essential microbiome. However, caution should be exercised when extrapolating rodent germ-free syndrome to humans, as the degree of dependence on microbiota may vary.^319^

## To adapt or not to adapt, that is a question

The relationship between the host and microbes has been a longstanding topic of interest among biologists. Examples that best illustrates their close relationship are that mitochondria and chloroplasts are cellular organelles that evolved through endosymbiosis,^320^ with each playing a key role in cellular energy metabolism and photosynthesis, respectively.^321,322^ Mitochondria, which are thought to have originated from an ancient *Alphaproteobacteria*, emerged around 1.5 to 2 billion years ago.^323^ Chloroplasts, on the other hand, originated from *cyanobacteria* and are estimated to have been incorporated into their host cells around 1 billion years ago.^324^ Recently, the nitrogen-fixing cyanobacterium UCYN-A has been proposed as an organelle called the nitroplast in *Braarudosphaera bigelowii*, attracting significant interest.^325^

In addition to endosymbionts,^326^ looking back through history, the term ‘symbiosis’ was first coined by Adolf Meyer-Abich in 1943 to describe the state in which more complex organisms live in association with simpler ones.^327^ In 1991, Lynn Margulis introduced the term ‘holobiont’ to describe a single organism and the collection of all the microorganisms within it, which highlighted symbiosis as a source of evolutionary innovation.^328^ However, “superorganism“^45,46^ and “meta-organism”^329–335^ have risen to prominence in contemporary literature and media, reflecting an extension with their original definitions. To be specific, introduced by William Morton Wheeler in 1911, ‘superorganism’ was primarily used to describe social insect colonies such as ants, bees, and termites, which exhibit a high degree of organization and integration. Within these colonies, individual members have clear divisions of labor and work collaboratively towards the survival and reproduction of the group, functioning as if the entire colony were a single organism.^336^ While Graham Bell in 1998 posits “metaorganism” as a singular multicellular organism like Volvox, serving as a good model for the study of the origins of multicellularity.^46^ After that, at the genetic level, the concept of ‘hologenome’, the combination of host genome and microbiome, was subsequently introduced by Richard Jefferson in 2007, emphasizing microbes as an essential component of organismal function.^337^ Rosenberg and Zilber-Rosenberg further developed the theory of hologenome evolution in 2007/2008, stating that the holobiont and hologenome are independent units of evolutionary selection.^338–341^ To resolve possible controversies, Bordenstein and Theis proposed ten principles to better understanding the hologenome and holobionts.^44^ Overall, they did not regard the organism as an independent organism but emphasizes holobiont and hologenome is the fundamental biological and evolutionary unit and all animals and plants exist as holobionts, these entities exhibit unique anatomical, metabolic, and immunological traits that contribute to their development and evolutionary processes.^340^ Their genetic information can transmissible across generations, collectively shaping the distinctive characteristics of the holobiont.^340^ Genetic variation within the hologenome stems not only from the host genome but also from the microbiome .^340^ The latter, with its capacity to adapt more swiftly to environmental changes, plays an essential role in the adaptability and evolution of the holobiont.^340^ For example, coral, a small and simple marine invertebrate contribute to the formation of coral reefs through their collective calcium carbonate secretions, not only possesses its own genome but also forms a holobiont genome with various microorganisms, such as *Symbiodinium*.^338^ The corals provide essential shelter and inorganic nutrients to *Symbiodinium*, while *Symbiodinium* supplies the corals with energy-rich organic matter through photosynthesis, meeting up to 95% of the corals’ energy needs.^342^ These microscopic algae have undergone a series of adaptive evolutions in the process of adapting to their symbiosis with corals including photosynthesis, ion transport, synthesis and modification of amino acids and glycoproteins, as well as responses to environmental stress.^342^ The holobiont genome enables corals to rapidly adapt to environmental changes more swiftly than it could rely solely on its own genetic mutations.^343–345^ In the melon and grape plants subjected to grafting experiments, a detailed analysis of the composition of the root endospheric microbial communities revealed a distinct pattern of deterministic assembly.^346^ To be more specific, the rootstock played a predominant role in recruiting the microbial community which means that the composition of microbial communities was influenced in a non-random manner by the genetic characteristics of the host plants.^346^ Vampire bat, one of the only three species of obligate blood-feeding mammals, whose genomes and microbiomes have co-evolved to meet the unique challenges posed by a hematophagous diet.^347^ The bats’ genomes show adaptive changes for this lifestyle, including morphological adaptations such as sharp incisors and canines, sensory adaptations with the positive selection of the *PRKD1* gene for locating blood vessels, digestive adaptations like the loss of sweet taste receptor genes and a significant reduction in the number of bitter taste receptor genes, and their immune systems have evolved to combat common blood-borne pathogens.^348^ Their microbiome have also undergone positive selection for genes that collaborate with energy production (involved in metabolic pathways such as the reverse Krebs cycle, enabling the derivation of energy from blood components), carbohydrate metabolism (enabling the breakdown and utilization of scarce carbohydrates found in blood), vitamin synthesis (including genes for biosynthesis of essential vitamins, such as carotenoids, which aid in immune function), fat storage (with key genes like glycerol kinase critical for the formation of triacylglycerol and fat storage, managing energy reserves), immune protection (enriched with protective bacteria like *Amycolatopsis mediterranei*, known for producing antiviral compounds, and genes from bacteria such as *Borrelia* and *Bartonella*, adapted for transmission by sanguivorous species), and metabolism of iron and urea (including genes for iron storage like ferritin light and heavy chains, and microbial genes like urease subunit alpha for urea degradation, addressing the challenges of high protein intake and nitrogen waste management).^348^ As a classic example of homogenome vertical inheritance, *Buchnera aphidicola* is an obligate intracellular symbiotic bacterium that forms a specialized mutualistic relationship with aphids, characterized by a streamlined genome that retains only the essential genes required for synthesizing amino acids vital to its aphid host, while lacking genes for cell surface components and cellular defense mechanisms, indicative of its adaptation to the stable environment within the host’s bacteriocytes.^349,350^ This symbiotic bacterium reproduces within the aphid’s specialized cells and is maternally transmitted to offspring, ensuring the continuation of the symbiotic relationship across generations.^351^ The interdependence between *Buchnera* and the aphid is manifested at the genomic level, with neither being capable of independent survival without the other.^352^

Currently, the evidence supporting the coevolution of hominids and microbes is gaining strength. Tracing the evolutionary threads of key gut bacteria such as *Bacteroidaceae* and *Bifidobacteriaceae*, revealing that these lineages have cospeciated with humans, chimpanzees, bonobos, and gorillas over an extensive period spanning 15 million years.^54^ This profound coevolutionary synchrony has led to a harmonized diversification across the nuclear, mitochondrial, and gut bacterial genomes, indicating they have a deep-seated and intimate relationship. Research by Suzuki et al. further supports this, showing codiversification between human populations and their gut microbiota across Europe, Asia, and Africa.^53^ It highlights the emergence of microbial strains with population specificity, potentially due to a shared evolutionary history, and species that have adapted to host dependency with traits like reduced genomes and sensitivities to oxygen and temperature.^53^ Moreover, the earliest fossil records indicate that the origins of *enterococci* can be traced back to the time of animal terrestrialization, approximately 425 to 500 million years ago.^353^ The diversification of *enterococci* has paralleled that of their hosts, particularly following the rapid emergence of new species after the Permian-Triassic extinction event.^353^ Despite constituting less than 0.1% of the human gut microbiome, *enterococci* have become prominent multidrug-resistant, hospital-adapted pathogens.^353^ This adaptability is largely attributed to the hardened cell wall they developed during the early terrestrialization process, along with their resistance to desiccation, starvation, and disinfectants, traits that have been crucial for their persistence in the modern hospital environment.^353^

Undoubtedly, the concept of “holobiont” and the “hologenome theory” offer profound insights into the understanding of the aforementioned phenomena. Although these concepts redefine the composition of individual unit, they do not fundamentally rewrite the theory of natural selection proposed by Charles Darwin and Alfred Russel Wallace.^44,354^ That is, natural selection remains the primary mechanism driving evolutionary change, the inheritance and variation of advantageous traits in the struggle for existence, leading to the gradual adaptation of species and the emergence of new ones.^44,354^ However, holobiont and hologenome extend the classical theories by incorporating the genetic contributions of the microbiome into the considerations of biological evolution and adaptive change, thereby offering a supplement for the modern evolutionary synthesis.^355^ As a simple understanding, natural selection can operates at multiple levels, including genes, individuals, populations, species, ecological communities, and holobiont entities, collectively shaping the diversity and evolution of life.^356^ In our limited comprehension, the concepts of holobiont and hologenome currently have areas that require refinement especially when apply these concepts to humans. Firstly, when considering the life cycle, these theories struggle to adequately explain the sequence of appearance between the host genome and the microbiome. For humans, the normal embryonic development occurs in the absence of live microbes within their own body—previous discussions have touched upon the fact that if there is an impact, it is more likely due to contamination. Microbes colonize during and after birth, and the assertion that all organisms are holobiont and homogenome may lead to the paradox that unborn baby are not considered biological entities. From a genetic perspective, the inheritance of microbes is not high-fidelity. Vertical transmission (through the birth canal of the mother) or horizontal transmission (acquired from the surrounding environment) are essentially forms of contact transmission, which cannot even be considered inheritance, accompanied by a certain degree of randomness. The example of *Buchnera aphidicola* is one of the exceptions that can be explained by the existence of hologenome inheritance, but it does not imply that other modes do not exist. The diversity of life constantly reminds us that when interpreting biological phenomena, we should allow for the existence of multiple patterns. Although we prefer to use a single pattern to explain all phenomena, this may limit our further thinking. When considering the driving phenomena behind the co-evolution of multicellular organisms with their microbes, it appears that the underlying potential causes have not been well pointed out, and currently, more attention is given to a general phenomenon. More research is needed to verify whether the evolution of microbes is towards a direction more beneficial to the host or towards a direction more beneficial to the microbes themselves. Determining who leads whom is crucial. We may consider that the host’s proactivity is the most likely driving force behind the happening of homogenome, a notion that is also evident from the discussed examples, just as the ecological environment of the Earth largely dominates the diversity of life, and the host can also be the natural selective force for microbes. In terms of application, the hologenome theory may not be a host-centered theory. Therefore, there is a lack of refinement in the framework of their interrelationship with the host’s interests at the core.

**Fig. 3:**
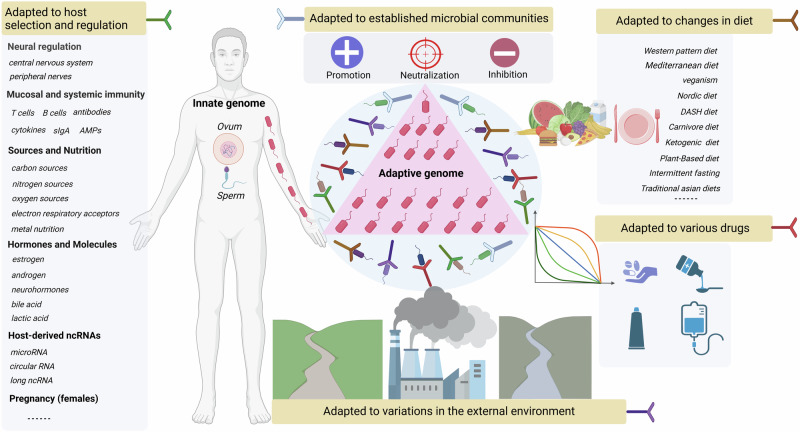
The meaning of adaptive genome. Human sperm and egg form the innate human genome. Microbes, through various selections, become the adaptive genome. Adaptive genomes may adapt to host selection and regulation, the dynamics of established microbial communities (which may promote, inhibit or remain neutral),^698–700^ exposure to different diets and drugs, and fluctuations in the external environment. DASH: Dietary Approaches to Stop Hypertension

To better delineate the evolutionary boundaries between the human genome and the microbiome, we can distinguish their characteristics using the terms “innate genome” and “adaptive genome.” The innate genome, humans are born with, forms the foundation of our biological identity, comprises a complete set of human nucleic acid sequences that can be inherited across generations following “Mendelian inheritance” and develops into organ systems, performing physiological functions in an organized manner. Conversely, the microbiome is acquired, regulated, and subject to dynamic changes, resulting in extensive biological crosstalk with the innate genome, ultimately affecting health and disease development (Fig. 3). They serve as an adaptive genomic repertoire for humans to adapt to general external environment.^357–359^ However, the notion of the microbiome as a “second genome” to humans maybe inappropriate.^360^ The terms “first” and “second” imply a sequential relationship within the same entity, whereas humans and their associated microbes are distinct species with their own evolutionary trajectories. The innate and adaptive genomes, together with the concepts of histological, immunological, host and homeostatic regulation proposed in this paper form a systematic framework to help us better understand the human-microbiome relationship and its interactions at all levels. Next we further discuss how the microbiome acts as an adaptive genome.

### Adaptation to the selection, control and regulation of the host

#### Immunological and neurological regulation

The immune system plays a crucial role in regulating the microbial community by utilizing both innate and adaptive immunity, and mucosal and systemic immunity. The central nervous system and the peripheral nervous system are indispensable components in the coordination of immunity. The brain engages in intimate communication with the immune system through its extensive neural networks and chemical messengers. Specifically, neurons in the central amygdala and the paraventricular nucleus of the hypothalamus express corticotropin-releasing hormone (CRH).^361^ The axons of these CRH neurons extend to the spinal cord and sympathetic nervous system, ultimately connecting with the splenic nerve to interact with immune cells in the spleen.^361^ Within the spleen, norepinephrine stimulates T cells to produce acetylcholine, a neurotransmitter that further acts on B cells, particularly through the α9 subtype of nicotinic acetylcholine receptors (nAChRs), promoting their activation and differentiation into plasma B cells.^361^ These plasma B cells are responsible for antibody production in circulation, a crucial component of the adaptive immune response.^362^ Indeed, the nervous system also can detects inflammatory cytokines like IL-1β, TNF-α, and IL-6 via sensory nerve terminals, relaying signals through the vagus nerve to the nucleus tractus solitarius in the brainstem initiating a neuro-immune feedback loop that regulates body inflammatory responses.^363^

The recognition and regulation of commensal microbes are carried out by CD4+ effector T cells and innate lymphocytes.^364^ T cell receptors (TCR) recognize widely conserved, highly expressed bacterial surface antigens.^364^ Th17 is induced by commensal microbes to express cytokines IL-17 and IL-22, which maintain a non-inflammatory state, while pathogenic bacteria induce Th17 cells to express IFN-γ and TNF, leading to an inflammatory state.^365^ Mucosal immunity produces secretory immunoglobulin A (sIgA), which limites commensal microbes to specific microbial niches in the body, thereby preventing pathogen isolation and spread.^366–368^ IgA deficiency has been associated with an overgrowth of *Candida albicans* in the intestinal tract and increase systemic immune dysregulation.^369–371^ However, when microbes invade the epithelium, they bind to FcαRI (CD89) on pro-inflammatory cells and cause a local inflammatory response.^372^ SIgA also has the ability to modulate microbial gene transcription, promoting the growth of beneficial bacteria while inhibiting the growth of harmful microbes.^373^ A recent interesting study has found that in the aged pituitary, growth hormone-secreting cells can independently produce IgA without the need for B cells, a process that is positively regulated by the diversity of the gut microbiota.^374^ It is speculated that this might serve as a compensation for the aging immune system, although the low level of IgA signals in pituitary cells has limited further understanding of this issue.^374^

Antimicrobial peptides (AMPs) or host defense peptide (HDPs), possesses antimicrobial activity in response to infection^375,376^ The secretion of IL-18 by enteric neurons is necessary for the expression of AMPs in goblet cells.^377^ Specifically deleting IL-18 in enteric neurons leads to a decline in the mice’s resistance to *Salmonella typhimurium* infection, manifested by greater weight loss and increased bacterial abundance in the cecum, liver, and spleen.^377^ In fact, AMPs exert their effects with distinct specificity with not merely functionally overlapping.^375^ For instance, Paneth cells secrete Peptide YY, an antifungal peptide that is released into and retained within the intestinal mucus layer.^378^ This peptide selectively inhibits the invasive and pathogenic hyphae of *Candida albicans* while having minimal impact on the yeast form that coexists with the human body.^378^ The evolution of certain AMP gene families could be an adaptive phenomenon.^379–381^ Fruit fly mutant analysis has detailed the distinct roles of antimicrobial peptides DptA and DptB, with DptA combating the pathogen *Providencia rettgeri* and DptB targeting the *Acetobacter*.^382^ The presence of these genes in Dipteran insects is closely tied to the microbes in their environment, with gene loss or pseudogenization occurring in the absence of these specific microbes.^382^ Interestingly, human AMPs (e.g. LL-37, a member of the cathelicidin family) were found to synergize with the Sh-Lantibiotics, secreted by the skin commensal *S. epidermidis* and *S. hominis*, to efficiently and selectively kill the *S. aureus*.^383^ So decreased secretion of AMPs such as defensins, lectins, lysozyme, ribonucleases, and cecum toxins can leads to increased susceptibility to certain pathogens.^384,385^ Therefore, in this section, it may be more accurate to understand AMPs as microbial regulatory peptides (MRPs) as they do not remove all microorganisms, but rather play more of a regulatory role.

#### Host sources

Sources of oxygen, carbon, nitrogen, electron transport chain receptors, and trace metals from the host form a nutritional ecological niche that regulates the selection and abundance of microorganisms. The colonization of facultative anaerobic bacteria gradually transforms the aerobic environment of the early intestine into an anaerobic environment, which favors the survival of obligate anaerobic bacteria.^41^ Obligate anaerobic bacteria further increase the oxygen consumption of IECs by producing metabolic products such as SCFAs, inducing the expression of hypoxia-inducible factor 1 (HIF-1)-related genes, affecting the metabolism of IECs, enhancing the tight junctions of IECs and promoting the production of mucus and antimicrobial peptides, thus shaping the microbiota group.^41,386,387^ In pigs, Yang et al. indicated that a 2.3 kb deletion in the ABO blood group gene, which occurred millions of years ago, led to a decrease in the concentration of N-acetylglucosamine (GalNAc) carbon sources in the intestine, directly affecting the nutritional metabolism of the family *Erysipelotrichaceae* and reducing its abundance.^388^ In contrast, in humans, it is *Faecalibacterium prausnitzii* and *Collinsella aerofaciens* that are capable of utilizing GalNAc.^389^ *Bacteroides acidifaciens* and *Akkermansia muciniphila* can utilize host-derived mucin as the nitrogen source.^390^ The latter is considered a potentially beneficial probiotic that can effectively regulate the host immunity and metabolism.^391^ It is worth noting that electron transport chain receptors from the host also flexibly regulate the microbiota. The epithelial cell NADPH oxidase 1 (Nox1) produces hydrogen peroxide (H2O2), preventing anaerobic bacteria from surviving in the inner layer of the mucus and crypts.^392^ Trace metals play critical roles as structural and cofactor elements in approximately one-third of proteins; however, excessive accumulation can lead to metal toxicity.^393^ Essentially, the body can suppress microbial replication, transcription, metabolism and survival by limiting or sequestering metals (such as iron, zinc, manganese, and copper) in the mucosal barrier and circulation.^393,394^ Immune cells can also employ a metal intoxication strategy by using zinc and copper to fight microbes in lysosomes.^393^ This “nutritional immunity” has recently been elegantly reviewed and may be exploited for the development of novel antimicrobial therapies.^393^

#### Extracellular vesicles

Host-derived extracellular vesicles (EVs) are a type of vesicle secreted by cells into the extracellular space.^395^ The nucleic acids carried by EVs, including miRNAs, circRNAs, and IncRNAs, have been shown to regulate microbiota.^396^ miRNAs in EVs can enter bacteria and regulate microbial gene transcription. For instance, miR142a-3p secreted by IECs can bind to targets in the commensal bacterium *Lactobacillus reuteri*, promoting its growth.^397^ Secretion of factors like IL-11 in response to the microbiota can influence the expression of host cellular circRNAs, subsequently modulating the levels of corresponding miRNAs.^398^ Interestingly, this host response affects the capability of cancer cells to metastasize.^399^ Moreover, the IncRNA expression profiles of germ-free mice differed significantly from those of conventionally raised mice and can be used in distinguishing between colonized *E. coli* strains or fecal-derived microorganisms.^179^

#### Hormones and metabolites

Hormones and metabolites circulating in the host body also affect the regulation of microbiota. Lyte and Ernst pioneered the field of microbial endocrinology, noting the impact of stress-activated neuroendocrine hormones on bacterial growth.^400^ Their work laid the foundation for subsequent discoveries that microbes possess hormone receptors, suggesting a role in intercellular messaging.^401^ Specifically, the gut microbiota of castrated male mice was more similar to that of female mice, suggesting that androgens may play a role in regulating the microbiota.^402^ Estrogen can reduce the abundance of *Proteobacteria* by activating estrogen receptor beta (ERβ) and increasing α-diversity of the gut microbiota.^403^ Ovariectomy (OVX) leads to increased levels of LPS in the serum, elevates the ratio of *Firmicutes* to *Bacteroidetes*.^404^ The decline in sex steroid levels, commonly experienced during menopause, enhances intestinal permeability and initiates inflammatory responses in the small intestine and bone marrow. This process stimulates the production of osteoclastogenic cytokines such as TNFα, RANKL and IL-17.^405^ Consequently, these cytokines promote osteoclast formation and activity, leading to bone loss, which may underlie the development of osteoporosis in postmenopausal women.^405^ Catecholamines can directly regulate bacterial gene expression, alter biofilm formation by *Staphylococcus aureus*, and downregulate the resistance of *Salmonella* to AMPs.^406–408^ Host metabolites, such as serum lactate, can be utilized by *Veillonella atypica* to produce propionate, which provides more energy to meet the body’s metabolic demands during exercise.^409^

#### Pregnancy

During normal pregnancy, women experience significant changes in their hormonal, immune, and metabolic profiles, which are reminiscent of the characteristics of metabolic syndrome.^410^ Research has indicated that the gut microbiota of pregnant women undergoes substantial alterations throughout gestation, particularly in the third trimester, where there is a notable increase in microbial diversity and a rise in the relative abundance of certain bacterial groups such as *Proteobacteria* and *Actinobacteria*.^411^ These shifts in microbial composition are closely associated with the host’s state.

From a macroscopic perspective, during human evolution, various modes have been developed to regulate the commensal relationship with the microbiome. Variations in regulatory patterns caused by host factors such as gender dimorphism,^412–414^ age (developmental stage),^415^ genetics (ethnic origins, genetic mutations),^416,417^ behavior (sleep, stress, exercise),^418–420^ and disease status (infection, activity, organ failure)^421,422^ may affect the variability of adaptive genomic profiles among individuals.

### Adaptation to variations in diet

Food serves as a fundamental requirement for sustaining human growth, reproduction and health.^423^ The sources, variety, and quality of the food we consume have a profound impact on the composition, diversity and richness of our microbiome.^424–426^ Differences in the gut microbiota across regions and countries may be attributed to dietary practices.^427^ Microorganisms can rapidly and reproducibly respond to ingested nutrients, exhibiting a simultaneous and consistent convergence in the changes observed between humans and other mammals.^334,428,429^ Studies have suggested that dietary protein (e.g. animal protein, whey protein isolate, and pea protein isolate) can enhance microbial diversity, whereas a high-fat diet (HFD) can significantly reduce the abundance of gut Lactobacilli and increase the proportion of *Clostridium*, *Bacteroides* and *Desulfovibrio* (producing propionate and acetate).^424^ *The genus Desulfovibrio* can also produce significant amount of leucine when exposed to HFD, which activates the mTORC1 signaling pathway in myeloid progenitors, fostering the differentiation and proliferation of polymorphonuclear myeloid-derived suppressor cells (PMN-MDSCs).^430^ This mechanism is integral to the “gut-bone marrow-tumor” axis, facilitating the progression of cancers including breast cancer and melanoma.^431^ Digestible carbohydrates, such as starch and sugars (glucose, fructose, sucrose and lactose), can increase the abundance of *Bifidobacterium* and decrease the abundance of *Prevotella*, whereas, artificial sweeteners show the opposite trend.^424,432^ Polyphenols, found in various fruits, vegetables, seeds, and beverages (e.g., beer, wine, juice, coffee, tea, and chocolate), and in small amounts in grains and legumes, can significantly reduce the number of pathogenic bacteria, such as *Staphylococcus aureus* and *Salmonella enterica*, while enhancing the abundance of beneficial microorganisms.^424^ Of all the nutrients, the effect of dietary fiber on microbes has been the most widely and intensively studied due to their wide-ranging benefits for human immunity and metabolism.^433^ Dietary fibers may influence the gut microbiota by: (1) providing direct nutrient substrates, such as resistant starch, which is utilized by *Ruminococcus bromii* in the colon;^434^ (2) activating microbial enzyme systems, such as *Bifidobacterium*, which uses its enzymatic capabilities to effectively metabolize galactooligosaccharides, thereby increasing its presence and activity in the intestinal tract;^435^ (3) regulating environmental pH, where the production of butyrate from fiber fermentation lowers intestinal pH and inhibits the growth of non-adaptive bacterial species;^436^ and (4) facilitating cross-feeding, where primary decomposers such as *R. bromii* breaks down resistant starch into short-chain fatty acids, which are subsequently utilized by secondary decomposers such as *Faecalibacterium prausnitzii*.^437^ A recent comprehensive assessment supports specific dietary fibers selectively enhance the abundance of certain gut bacteria—carrageenan increases *Phascolarctobacterium*, *Prevotella*, and *Treponema*; xylan boosts *Butyricimonas*; arabinogalactan augments *Bacteroides*; and β-glucan promotes *Lactobacillus*.^438^ Conversely, the abundance of *Clostridium perfringens* and *Bacteroides fragilis* is reduced by a range of fibers, including arabinoxylan, apple pectin, xylan, arabinogalactan, xanthan gum, guar gum, carrageenan, glucomannan, and β-glucan.^438^ In particular, Cynthia et al. reviewed over 1500 human fiber intervention studies, integrating 16 S rRNA amplicon data from 2368 gut microbiome samples from 488 participants.^439^ The above robust dataset offers strong clinical evidence, enabling a comprehensive assessment of human microbiome’s response to various types of dietary fiber.^439^ The interplay between diet and the gut microbiome contributes to the fluctuation of serum metabolites, which in turn correlates with the alteration of specific clinical indices. For example, systolic blood pressure is specifically decreased by the consumption of vegetable oil, which enhances the abundance of the *Blautia* and concurrently lowers the serum levels of 1-palmitoyl-2-palmitoleoyl-GPC (16:0/16:1).^440^ In addition, fruit consumption exerts a similar blood-pressure-lowering effect by elevating the serum levels of threonate, a metabolite enhanced by *Blautia*.^440^ Overall, different dietary patterns, such as Western diet,^441^ Mediterranean diet,^442–445^ veganism,^446^ Nordic diet,^447^ Dietary Approaches to Stop Hypertension (DASH) diet,^448^ Carnivore diet,^449^ Ketogenic diet,^450^ plant-based diet,^451^ intermittent fasting,^452^ and traditional Asian diets,^453^ provide various combinations of nutrients, thereby changing the composition and richness as well as function of surviving microbes. Harnessing dietary interventions to modulate the microbiota represents a promising avenue for optimizing human metabolism.^426,454^

### Adaptation to environmental conditions

In contrast to genetics, environmental factors are better able to explain the variations observed between microbial communities.^455,456^ Due to geographical isolation and the impact of human migration, *H. pylori* populations in different regions exhibit varying levels of genetic diversity.^457–459^ For instance, the hpEurope population demonstrates a higher degree of genetic variability as a consequence of multiple historical waves of human migration.^457,458^ Analysis of 13,000 publicly available metagenomic samples using artificial intelligence has revealed a close relationship between microbial genes and their habitat.^460^ Systematic summary has been reported indicating striking differences of the gut, oral cavity, respiratory tract, skin and urinary tract microbiome across different populations worldwide.^461^ Within 3224 Chinese individuals, researchers found that geographical factors are the most significant external influences on the composition of the gut microbiome, with the similarity of the microbiomes among individuals being inversely proportional to their geographic distance.^440^ Interestingly, gut microbes share approximately 48.6% similarity in the cohabitation scenario.^456^ Furthermore, airway microbiome can serves as a mediator in the influence of environmental pollution factors on respiratory system health.^462^ With the transition of humans from a primitive hunting and farming lifestyle to an urban lifestyle, there has been a decline in microbiome diversity. Specifically, the gut microbiomes of primitive civilizations were characterized by high abundance of *Prevotella*, *Aspergillus*, *Spirochetes* and *Clostridium*, whereas those of urban dwellers typically contain *Bacteroides*, *Bifidobacteria* and *Firmicutes*.^461^ Western urban populations appear to have lost microorganisms such as dense intestinal spirochetes, possibly due to multiple factors, including changes in dietary habits and modern drug treatments, as this microorganism is still retained in other primates (excluding the effects of climate change).^461^ Of all the microbiomes, the skin, which is exposed to the natural environment, appears to be the most affected. For example, in rural areas, they were more exposed to soil and environmental microbial sources, while in urban areas, participants worked indoors and had less access to these sources.^463^ These environmental differences may explain the differences in the abundance of *Trabulsiella* and *Propionibacterium* in the participants’ skin.^463^ A systematic review reported that greenspace exposure was associated with increased microbial diversity as well as alterations in the overall composition of the microbiota in the gut and skin.^464^ Specifically, there were increases in the relative abundance of beneficial bacteria (e.g., *Ruminococcaceae*) and decreases in the relative abundance of harmful bacteria (e.g., *Streptococcus* and *Escherichia/Shigella*).^464^ However, regionalized differences also pose challenges in establishing uniform microbial-related predictors and models.^465^ The space environment, which includes microgravity and radiation, could profoundly influence on the physiology, genetics and community composition of microorganisms. During short-term spaceflight, the skin microbiome exhibits a temporary increase in viral populations, including *Uroviricota*, *Cressdnaviricota* and *Phoxiviricota*.^466^ In the oral microbiome, there is an observed increase in bacterial groups associated with periodontal disease and dental caries, such as *Fusobacteriota*, with notable species like *Fusobacterium hwasookii*, *Fusobacterium nucleatum* and *Leptotrichia hofstadii*.^466^ Spaceflight also enriches microbial genes related to phage activity, toxin-antitoxin systems and stress responses across multiple body sites, indicating adaptive changes of microbes to the stressors of the space environment.^466^ Although most of these changes are transient, such as *Corynebacterium species*, showed a temporary decrease in transcriptional activity,^466^ some bacterial groups in the skin microbiome, like *Acinetobacter spp*. demonstrated a persistent reduction.^466^

### Adaptation to various medications

Medication can alter the intestinal microenvironment, influencing microbial growth or undergoing direct microbial metabolism, ultimately modifying the composition and function of the microbiota.^467,468^ Antimicrobial resistance is a common survival mechanism in both pathogens and commensal bacteria.^469^ Initially, six-month antibiotic treatments for tuberculosis disrupt the gut microbiome, allowing drug-resistant pathogens to dominate.^470^ However, commensals soon overtake them through competitive adaptation.^470^ Additionally, non-antibiotics like antidepressants can increase mutation rates and speed up the horizontal transfer of resistance genes by activating bacterial defense mechanisms.^471^ With the expansion of research, our understanding of the effects of drugs on microorganisms has extended to more medications.^472^ Proton pump inhibitors (PPIs), a type of medication used to treat digestive disorders, can reduce the acidity of gastric fluid and increase the number of oral microorganisms that migrate to the intestine.^473,474^ Metformin, a traditional medication for lowering glucose, can increase the abundance of mucin-degrading bacteria *Akkermansia muciniphila* and butyrate-producing bacteria.^475^ In addition, data from 2173 individuals in the European Heart and Metabolic Disease cohort showed that 28 drugs significantly affected the microbial characteristics, demonstrating a combination, cumulative and dose-dependent effect.^476^ The synergistic effect of multiple drugs can redirect the host microbiota to a more favorable state, and an increase in the number of antibiotic courses is associated with an increase in the abundance of harmful microorganisms.^476^ Microorganisms can chemically modify oral medications to produce different functional and pharmacological properties. For example, 5-aminosalicylic acid (used to treat ulcerative colitis) relies on colonic bacteria to cleave azo bonds and release active drugs in the colon;^477^ and digoxin (used to treat heart disease) can be inactivated by *Eggerthella lenta*.^478^ Similarly, *Gardnerella vaginalis*, the dominant bacterium in the vagina, can predict poor outcomes for tenofovir (a pre-exposure prophylactic drug for HIV infection), whereas Lactobacilli can increase its efficacy threefold.^479^ The β-glucuronidase of commensal bacteria can convert irinotecan (a prodrug for colon cancer treatment) into a toxic form, killing intestinal epithelial cells and causing severe diarrhea.^480^ In a study by Zimmermann et al. of the 271 oral medications tested, two-thirds could be metabolized by various strains of intestinal bacteria, and each strain could metabolize 11-95 drugs.^481^ Recently, a team established a drug metabolism model based on the genomes of 7,302 microorganisms to explore personalized prediction and analysis.^482^ In addition to activating, inactivating, and exhibiting toxic effects on various medications, microorganisms have also received widespread attention for improving clinical responses to immune therapy for cancer treatment.^483–485^ Further studies of the interplay between the adaptive genome and the innate genome’s drug response, absorption, distribution, metabolism, and excretion show potential in predicting disease treatment efficacy and improving individual drug efficacy levels, leading to Precision-Comprehensive Drug Microbiology.

### Adaptation to established microbial communities

The human microbiota community is a complex ecological network. Established microbial communities influence newcomers positively, neutrally, or negatively.^486^ In vitro co-cultures of fecal microbiota show that 51.67% of these relationships are neutral.^487^ Commensalism, which make up 21.67% of interactions, occur when one organism benefits from another without harm, or both benefit from each other.^488^ This often occurs through cross-feeding between microbes, such as when *Acetobacter pomorum* obtains lactic acid from *Lactobacillus plantarum* and in turn produces amino acids essential for the growth of *Lactobacillus plantarum*.^489^ In addition, bacteria can cooperate to form biofilms-aggregations that enhance protection and competitive advantage by conferring antibiotic resistance to their members.^490^ Interestingly, a recent discovery involves an oral *Saccharibacteria isolate* (TM7x) that protects its host bacterium, *Schaalia odontolytica* (XH001), from predation by the *lytic bacteriophage* LC001.^491^ Antagonistic relationships account for 18.33%, where one organism inhibits another without harming itself.^492^ Some bacteria can secrete antibiotics or other inhibitory substances to suppress competitors and maintain resource dominance.^493^ Competitive interactions, which account for 5%, involve two species competing for the same resources and adversely affecting each other.^494^ For example, in vitro cultures of *Intestinimonas butyriciproducens* and *Shigella flexneri* showed inhibited growth when in close proximity.^487^ Exploitative relationships, which account for 3.33%, are similar to predatory relationships in which one organism uses another as a resource.^495^ A classic example is bacteriophages, which are often described as obligate predators of bacterial hosts, achieving reproduction through mechanisms such as generalized transduction, specialized transduction, and lateral transduction.^496^ However, Shkoporov et al. suggested that at the population level, phage-bacteria interactions may facilitate long-term coexistence.^496^ In the face of constant phage threats, bacteria are forced to continuously produce genotypic and phenotypic variants, reducing the sensitive subpopulation through lysis and allowing the spread of resistant mutants, thus increasing intraspecies diversity.^496^ It is crucial to emphasize that higher-order interactions within microbial communities are key drivers explaining the characteristics of microbial consortia.^497^ Quantifying the genetic adaptability of *E. coli* in different communities (from two to four species) suggests that these higher-order interactions significantly affect microbial gene expression and function.^497^ Even in simple microbial communities, the dynamics of interactions are complex and essential for understanding microbial functional mechanisms. In fact, the specific microbes and the nature of their co-occurrence relationships can vary significantly between organs and are influenced by a range of factors including pH levels, immune responses and the presence of specific nutrients or other environmental conditions within each organ’s microenvironment. For example, the oral cavity and the large intestine are characterized by a higher incidence of co-exclusive relationships among microbes, while other organs show a more pronounced presence of co-occurrence relationships.^91^ Unraveling adaptive mechanisms is crucial for predicting the regulation of community dynamics and enhancing the success and stability of microbial transplants. However, this endeavor presents significant challenges. In this context, the study of keystone taxa - first introduced by Paine as species that play a critical role in the stability of their ecosystems may be a good strategy.^498,499^

## Host or Meta host

**Fig. 4:**
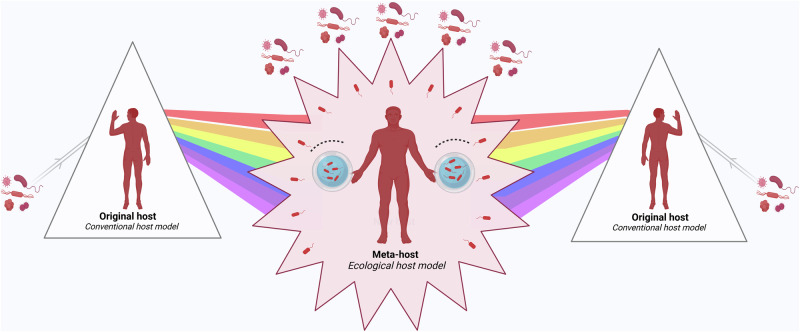
Original host (conventional host model) and Meta-host (ecological host model). The meta-host is used to describe conventional host that exhibit marked differences in colonization, susceptibility and pathogenicity to microorganisms following microbial accession. This phenomenon is the result of the dynamic integration of the adaptive genome with the innate genome and corresponds to the human ecological perspective

The introduction of the adaptive genome in above context can led to reconsideration of our understanding of the host, transitioning from a ‘host’ (innate genome) to a ‘meta-host’. As a meta-host, it offers a unique ecological niche for other organisms, thereby affecting the host’s susceptibility to infections, the pathogenicity of the infecting agents, and the severity of diseases (Fig. 4).^500^ Specifically, Miller et al. found that, compared to traditional mice, which are not typically suitable hosts for nematodes and tapeworms, were able to develop seemingly healthy and reproductive adults in germ-free guinea pigs when infected with *Nippostrongylus muffs*, *Nematospiroides dubius* and *Hymenolepis nana*.^501^ The study also indicated that the destruction of commensal microorganisms led to increased susceptibility to *Salmonella* infection in mice treated with antibiotics (streptomycin) after infection with a lower titer.^196^ In contrast, *Entamoeba histolytica* is known to be pathogenic in the intestinal tract of traditional guinea pigs, but not in germ-free mice. When *E. coli* was added to the inoculum, intestinal damage occurred, which was not caused by *E. coli* alone.^502,503^ Patients with nontuberculous mycobacterial lung disease (NTM-LD) exhibit a significant perturbation in gut microbiota, particularly marked by a decrease in *Prevotella copri*, which is strongly correlated with both the occurrence and severity of NTM-LD.^504^ This is accompanied by a reduction in TLR2 activation activity and a discernible immunosuppressive effect within the lungs.^504^ Angela Wahl and colleagues developed a germ-free humanized mouse model that demonstrates the human microbiota significantly enhances the infection and pathogenicity of Epstein-Barr virus (EBV) and human immunodeficiency virus (HIV).^505^ The presence of a normal microbiota in conventional humanized mice (CV-BLT) promotes more frequent EBV-induced tumorigenesis and higher levels of HIV replication in the gut and systemic circulation.^505^ In young South African women, it has been discovered that the diversity of the cervicovaginal bacterial community is also closely associated with the risk of HIV infection.^506^ Specifically, communities with high diversity dominated by anaerobic bacteria other than Lactobacillus have been found to increase the risk of HIV infection by more than four times.^506^ Certain bacterial groups, such as *Lactobacillus crispatus*, are correlated with a reduced risk of infection, while others like *Prevotella* and *Sneathia* are associated with an increased risk by increasing the number of activated CD4 + T cells in the reproductive tract, which are the target cells for HIV infection.^506^ Another highly regarded example is the significant changes observed in the composition of the gut, lower respiratory, vaginal, and oral microbiota in individuals infected with coronavirus SARS-CoV-2.^507–511^ These changes manifest as a striking enrichment of pathogenic microorganisms and a concomitant reduction in beneficial counterparts, and are closely associated with levels of biomarkers of inflammation and tissue damage suggesting a potential interplay between the microbial community and disease severity.^512^ The immune response and physiological milieu of infected individuals may induce these compositional changes, thereby compromising the host’s antiviral capacity.^513,514^ The diminished biosynthesis of immunoregulatory metabolites, such as butyrate and L-isoleucine further impairs the body’s ability to counteract viral invasion^515–517^ and the increased infectivity of microbial agents exacerbates the clinical prognosis.^518^ While comprehensive investigations are warranted to unravel the complex effects of the microbial community on viral susceptibility and transmissibility, these findings necessitate a reevaluation of our defensive and therapeutic strategies in anticipation of future infectious diseases. Such efforts hold the promise of mitigating the disastrous consequences of rampant viral outbreaks on the world’s population.^519^ Meta-Host model also show potential in the interpretation of organ transplantation heterogeneous outcomes. While the classic understanding points to genetic mismatches as the main cause of graft rejection, the unique microbiota of each individual might also play a crucial role. It can be evidenced by the extended survival of skin transplants in mice associated with the presence of *Alistipes*, suggesting that specific microbes could impact the host’s physiological responses.^520^ Sequencing of 1370 fecal samples from 415 liver transplant recipients and 672 kidney transplant recipients also indicated that ecological shifts in the human microbiota are associated with increased mortality rates following transplantation.^521^ This underscores the potential therapeutic potential of targeted microbiota manipulation, such as with probiotics or microbiota transfers, to improve transplant success rates.

## Do we manage a slave tissue?

The captivating role microorganisms play in human physiology has sparked the prevalence of the ‘organ’ theory. In 1992, Bocci proposed that the gut microbiota is an overlooked organ that is critical for immune stimulation in humans.^522^ Subsequently, in 2006, O’Hara et al. further elaborated on this theory, calling for a deeper understanding of this hidden organ to unlock secrets related to human health as well as various infectious, inflammatory and tumor diseases.^523^ Since then, the gut microbiota has been popularized as an “organ” in academic and popular media. In 2013, Burcelin et al. pointed out that bacteria are also found in common tissues such as the liver and adipose tissue.^524^ The interaction between host tissues and microorganisms may provide new opportunities for disease diagnosis, immune regulation and nutritional applications, although their review did not consider it as an independent tissue rather named “tissue microbiota hypothesis”.^524^ To further emphasize that host regulation is fundamental to the normal functioning of microbial organs, Byndloss and Bäumler proposed the “Germ-organ theory” in 2018 to describe how host dysregulation (such as epithelial dysfunction) can lead to the occurrence of non-infectious diseases (such as an increase in *Proteus mirabilis*).^525^ While the ‘organ theory’ deepens our appreciation for the significance of microorganisms, it has simultaneously sparked scientific debate. For example, Fucarino et al. contended against such perspectives, primarily because they adhered to the traditional definition of organs as structures composed of tissues with similar or varying embryonic origins within the human body, a criterion that microorganisms evidently do not meet.^526^ They proposed that the term “mucosal microbiota layer” of hollow organs more precisely encapsulates the role of microorganisms, as exemplified by the gut and respiratory tract ecosystems.^526^ However, this designation does not encompass the skin microbiota also falls short of offering a comprehensive systemic view on genetic and hereditary aspects.

**Fig. 5:**
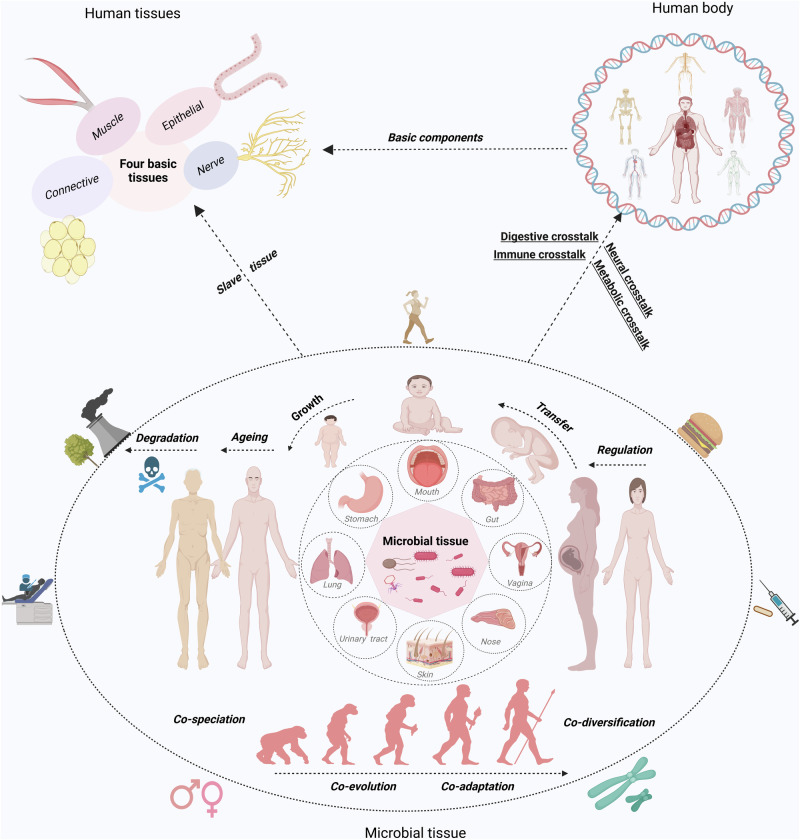
Slave tissue hypothesis. Microbial tissue is the additional fundamental tissue of the human body, a slave tissue alongside nervous, epithelial, connective and muscular tissues.^701,702^ The maternal microbiota exerts a regulatory influence on fetal growth and development and can partially transfer seed microbiota to the newborn through microbial exposure. Microbes that colonize in body site (including but not limited to the gastrointestinal tract, respiratory tract, reproductive tract, skin and urinary tract) play a vital role in digestion, immunity, neural regulation and metabolic crosstalk throughout human growth and ageing, and ultimately participate in the degradation of the body upon death.^37,415,703,704^ The human microbiota has undergone co-speciation, co-evolution, co-adaptation, and co-diversification with humans over a long period of time.^53^ Throughout the life cycle, factors such as mode of delivery, genetics, gender, diet, medication, environment and behavior (e.g. exercise) can potentially contribute to differential microbial tissue formation^705–707^

In fact, the traditional definition can be appropriately adjusted based on the motivation behind the traditional terminology. Medical classification of cells, tissues (a group of cells), organs (a group of tissues), systems (multiple organs) and organisms (multiple systems) is helpful for scientists to focus on different levels of research. Owing to limited detection techniques, we initially did not have a clear understanding of the interaction of microorganisms in human physiology and pathology, and it is not surprising that we did not consider them as part of human tissues and organs. Overall, the ‘organ theory’ has stimulated greater research interest and clearly given microbial communities the attention it deserved. To better manage microbes in our bodies, several issues related to our understanding of body composition need to be addressed. Firstly, in our understanding, human microbiota are more like a component of the tissues that make up organs, rather than being stand-alone organs themselves. For example, in the gut, certain microbes play a critical role in the complete performance of digestive functions which can be consider as part of gut organ. The incomplete gastrointestinal function exhibited as we discussed in germ-free syndrome also corroborates this point. That is to say, a gut devoid of microorganisms cannot be considered a complete intestine and is unable to perform the full range of functions associated with a healthy gut. Similarly, the skin is an organ whose complete defensive function is facilitated by a covering layer of microbial tissue. Second, on specific classifications, the composition and functions of microbial communities vary greatly at different anatomical sites. Designating each distinct microbial community as a separate organ would unduly complicate the existing human organ classification system. Classifying microbial communities according to their specific anatomical locations is more consistent with traditional methods of tissue classification, such as the further categorization of epithelial tissues into overlying epithelium and glandular epithelium. Microbial tissues can be similarly categorized by corresponding anatomical locations, such as skin microbiota, gut microbiota, etc. Hence, regarding microbes as distinct organs isn’t pragmatic. The final conundrum is that the traditional tissues are generally solid rather than fluid or dynamically mobile. Our previous discussions on how the microbiome adapts to and is regulated by host conditions provide a rationale for the emergence of the concept of “slave tissues”. As a hypothesis, the multicellular organisms may exhibit a form of “slavery” towards microorganisms, maintaining a constant struggle that has driven long-term evolution. This concept, borrowed from sociology, can partially elucidate the variations in microbes across different developmental stages of an individual’s life cycle. It also aids in explaining the acquired immunity and the models of host health and disease conversions. Most importantly, it expands the concept of histology to include an external, dynamic tissue, thereby resolving a nomenclature dilemma about microbiome in histology. Conversely, if microbes were to form an organ - implying significant proliferation - they could have fatal consequences for the human body. Overall, these tissues have developed from adaptive genomes and possess physiological functions closely tied to the four master tissues (epithelial, connective, muscular, and nervous) (Fig. 5). Loss of control (microbial dysbiosis) of microbial tissue may lead to malignant cycles of cardiovascular,^527–531^ respiratory,^532–534^ digestive,^535^ nervous,^536–540^ endocrine and metabolism,^541–543^ oral,^544,545^ skin,^102,103,546^ autoimmune,^547–549^ urogenital,^550^ mental disease,^551^ and even cancers,^552–554^ although the causality of related diseases so far is not always clear (Some of these diseases will be discussed in next sections). The formidable organizational structure of the human body’s composition is profoundly systematic. This is precisely why, even when confronted with a great number of dispersed microorganisms resembling the human body, they still maintain a dominant position. Hence, it is prudent to regard microorganisms as a subservient or controlled component of the system. The “slave” perspective also makes hygiene practices (brushing teeth, washing face, bathing, disinfecting) more understandable and acceptable. Their development, the ‘biofilm’ and ‘quorum sensing’ abilities can damage our health.^555,556^ Therefore, we still need to control their location, limiting unrestricted growth and widespread communication, avoiding serious damage to other tissues (Fig. 5).

Attempts to classify microbial tissues with greater precision have ventured into new territory. The notion is that these so-called “slave tissues” can be systematically categorized through an ecological framework of classification. For example, in 2011, the MetaHIT team proposed the concept of ‘enterotypes’, dividing the gut microbiota into three types.^557^ They are robust classifications which are not influenced by nationality or region and characterized by a unique composition and metabolic signature of the microbial community: Enterotype 1, characterized by *Bacteroides* and efficient in fermenting carbohydrates and proteins; Enterotype 2, marked by *Prevotella* and its mucin-degrading capabilities, often in synergy with *Desulfovibrio*; and Enterotype 3, defined by *Ruminococcus* and *Akkermansia*, specializing in mucin binding and sugar transport.^557^ In subsequent research, the authors emphasize that enterotypes are not strictly separated categories but rather exhibit a tendency for clustering within a continuous spectrum of gut microbial community composition. They also provided a standardized procedure and guidelines for enterotype analysis to enhance the accuracy and comparability of research findings across studies.^558^ Different research teams, however, employing a variety of experimental approaches, algorithms, and analytical techniques, classified the gut microbiota into diverse categories.^559,560^ Recently, Senying Lai et al. conducted an extensive analysis of 3363 fungal sequencing samples from 16 cohorts across Europe, North America, and Asia, further delineating the four fungal enterotypes of the human gut (mycobiome).^561^ The Sacc_type enterotype, dominated by *Saccharomyces cerevisiae*, is more prevalent among younger individuals and correlates with a better intestinal barrier.^561^ In contrast, the Can_type enterotype, characterized by the abundance of *Candida albicans*, is enriched in the elderly and associated with an increased risk of various diseases and a compromised intestinal barrier.^561^ The Asp_type enterotype, led by *Aspergillus* species, shows a correlation with certain bacterial enterotypes, while the Asc_type enterotype is driven by either unclassified *Ascomycota* or *Saccharomycetales*.^561^ With advancements in capsule sampling and microbial visualization techniques, we may anticipate a shift towards more efficient classification that isn’t solely reliant on fecal samples.^89,90,562^ In an analysis of the oral microbiome of 1500 Spanish adolescents revealed two predominant oral microbial community patterns, termed “stomatotypes”.^563^ The first pattern is dominated by the genera *Neisseria* and *Haemophilus*, designated as the Neisseria-Haemophilus stomatotype (Stomatotype 1), while the second is characterized by the dominance of *Prevotella* and *Veillonella*, known as the Prevotella-Veillonella stomatotype (Stomatotype 2).^563^ They hypothesized that these stomatotypes may represent two potential optimal equilibria of the oral microbiome on a global scale, prevalent across various geographical regions, lifestyles, and age groups.^563^ Another example is vaginal Community State Types (CSTs), a classification system used to describe the composition of vaginal microbial communities in women of reproductive age.^110^ Specifically, CST I is characterized by the dominance of a single species of *Lactobacillus*, in particular *L. crispatus*.^564^ CST II is similarly characterized by *Lactobacillus* dominance, primarily *L. gasseri*.^116^ CST III is dominated by *L. iners*, although its association with vaginal health remains unclear.^116^ CST IV lacks *Lactobacillus* dominance and includes other facultative and obligate anaerobic bacteria such as *Gardnerella*, *Prevotella*, *Atopobium*, *Sneathia*, *Megasphaera* and *Peptoniphilus*.^116^ This microbial composition is associated with bacterial vaginosis (BV) and may increase the risk of several adverse health outcomes.^116^ Finally, CST V is dominated by *L. jensenii*.^110^ In summary, the detailed classifications of microbial types will empower researchers to discern various microbial community states, enhancing our comprehension of their relationship with human health. This advancement in turn will unlock significant clinical implications, including disease vulnerability, diagnostic accuracy, and the efficacy of medical interventions.

## From homeostasis to homeostatic reprogramming

**Fig. 6:**
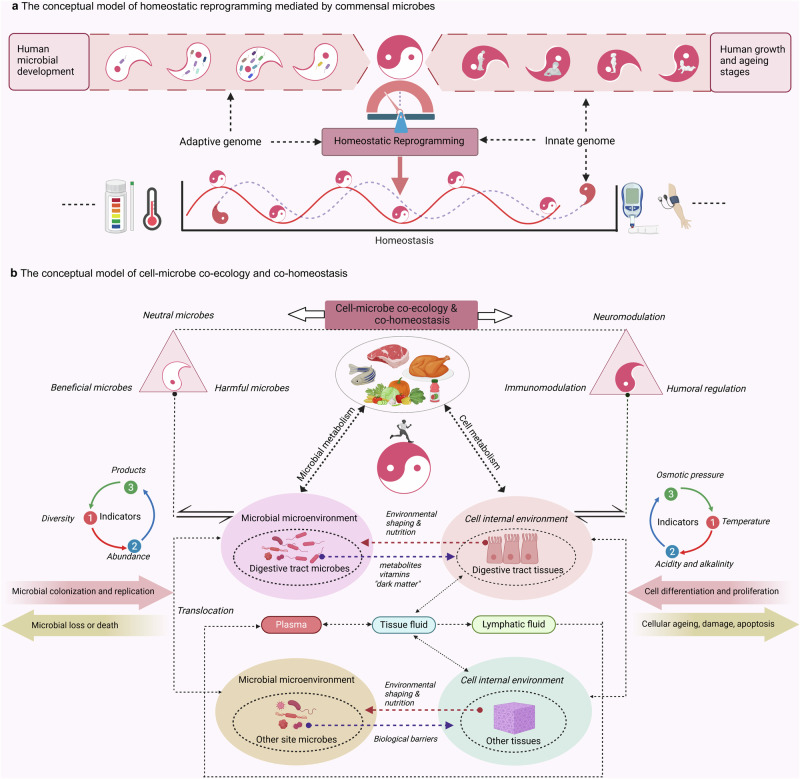
The conceptual model of homeostatic reprogramming mediated by commensal microbes. **a** The concept of ‘Homeostatic reprogramming’ is used to describe a phenomenon in which the adaptive genome (commensal microbiota) coordinates with the innate genome (human cell/tissues) to deviate the scope and regulation outcome from the original trajectory including body temperature, uric acid levels, glucose level, blood pressure, etc. The interplay between human life stages - from youth to old age - and microbial development - from increasing to decreasing diversity - overall results in different regulatory forces. **b** The conceptual model of cell-microbe co-ecology and co-homeostasis. Plasma, tissue fluid and lymphatic fluid form the internal environment of the human body’s cell life. This internal environment is regulated by the neural, immune, and metabolic systems to maintain a dynamic homeostasis of physical and chemical properties such as temperature, pH, and osmotic pressure. The internal factors of cell differentiation, proliferation, ageing, damage, and apoptosis can affect this homeostasis. Human tissues are involved in shaping a physico-chemical and nutritional environment where external microorganisms can colonize, replicate, experience loss and die. On the one hand, human cells, and microorganisms in the digestive tract work together to metabolize nutrients from food. Microbes not only affect nutrient absorption, but also produce metabolites, vitamins, and potential “dark matter”, which can enter the internal environment and affect its homeostasis. The imbalance of the internal environment also leads directly to the disruption of the microenvironment they form. The diversity, relative abundance, and products of beneficial, harmful, and neutral microorganisms (composition) are important indicators for assessing the environmental balance. On the other hand, microorganisms also participate in shaping the microenvironment by providing a barrier to respond to external environmental changes. This overall change regulates the susceptibility of the internal environment to external perturbations, acting another regulatory force for homeostasis

In our previous discussions, we concluded the discourse on the hypothesis of the existence of foreign, salve-like tissues within the human body. Next, it is essential to re-examine the foundational theory of physiological medicine—homeostasis, as without homeostasis, there is no health. The internal environment, comprising extracellular fluids such as interstitial fluid, plasma, and lymphatic fluid, represents the environment in which the cell lives.^565^ In the 19^th^ century, French physiologist Claude Bernard introduced the concept of “interior milieu,” emphasizing its stable and autonomous nature as a prerequisite for life. This property enables an organism to compensate for variations in the external environment.^565^ In 1929, the American physician Walter B. Cannon proposed the concept of “homeostasis”, highlighting the dynamic stability and regulation of the internal environment.^566^ Currently, it is widely acknowledged that the internal environment maintains the dynamic stability of chemical composition (water, inorganic salts, and organic compounds) and physico-chemical properties (osmotic pressure, pH, temperature) in a coordinated manner across tissues, organs, and systems through neural, metabolic, and immune regulation.^567,568^ Maintenance of the internal environment’s stability is critical for cellular metabolism and physiological functions of the body, and its disruption can result in diseases.^569^ The theoretical basis of the internal environment and homeostasis has made medical interventions possible. The hypotheses of adaptive genomes, slave tissue and germ-free syndrome, presents us with the opportunity to further expand the framework of homeostasis. Here, we introduce the concept of ‘Homeostatic reprogramming’ to describe a phenomenon in which the adaptive genome coordinates with the innate genome to deviate the scope and outcome of neuroendocrine and immune regulation from the original trajectory (Fig. 6a). In other words, the statement of maintaining homeostasis may not be accurate. A classic example of reprogramming is the possibility that ‘healthy’ body temperature is the result of a combined microbiota-host regulation. Intermittent changes in the external environment can induce changes in the resting metabolic rate, serum thyroid hormones and core body temperature in mice, while dynamic gut microbiota and their metabolites can provide the host with metabolic plasticity to regulate temperature fluctuations.^570^ Supporting this notion, antibiotic clearance of bacteria has been shown to damage body’s thermogenic capacity.^570^ Recently, it has been shown that changes in the gut microbiota can predict the temperature course of hospitalized patients with sepsis, and that GF or antibiotic-treated mice have lower basal body temperatures than those harboring natural microbiota.^571^ When exposed to cold, animals can produce heat and maintain body temperature by activating brown adipose tissue (BAT) and browning of white adipose tissue (WAT).^572^ The absence of certain microbiota inhibits the increase in uncoupling protein 1 (UCP1) expression in BAT and reduces the degree of fat browning.^572^ These findings suggest that the presence of an adaptive genome may lead to reprogramming of body temperature, and that animals lacking microbiota may experience impaired thermoregulation. Of greater concern is that this may partly explain the curious phenomenon of a 1.6% average decrease in human body temperature since the 1860s, the use of antibiotics, improvements in hygiene and an increase in processed foods, accompanied by a decrease in microbiota diversity and abundance.^573,574^ Uric acid (UA), as another example, is the end product of purine metabolism in the human body, and excessive accumulation can lead to metabolic imbalances. Recent findings have revealed widespread purine degradation and anaerobic uric acid metabolism within the gut microbiota.^575,576^ This microbial process appears to compensate for the host’s deficiency in UA-degrading enzymes, possibly stemming from a “thrifty gene” that gradually became inactive during human evolution to enhance adaptation to periods of hunger and cold by stimulating gluconeogenesis, increasing fat storage, and reducing fat oxidation.^577^ In rodent models lacking UA-degrading enzymes, depletion of gut bacteria results in severe hyperuricemia, while colonization with UA-consuming intestinal bacteria reduces UA levels.^575^ In retrospective patient studies, the use of antibiotics targeting anaerobic bacteria were associated with an increased risk of subsequent gout.^575^ Overall, the loss of UA-consuming microbes could partly account for the rising prevalence of hyperuricemia in modern times.^578^ Commensal microbes are also involved in the regulation of blood glucose homeostasis. Intestinal intrinsic enteric-associated neurons (iEANs), operating independently of central neural regulation, autonomously oversee functions such as intestinal motility and secretion.^579^ A subset of iEANs, characterized by CART+ neurons regulated by the gut microbiota, traverses from the intestines, establishing neural circuits with the liver and pancreas through the sympathetic nervous network.^579^ Activation of these neurons induces a decrease in insulin levels, a rise in blood glucose, and reduced food intake in mice.^579^ Microbial changes induced by non-nutritive sweeteners can causally lead to individualized alterations in blood glucose responses.^580^ GF mice exhibited lowered blood pressure levels, while population analyses of oral and gut microbial profiles indicated substantial correlations with blood pressure.^581^ The microbial regulation of blood pressure mechanisms has recently undergone comprehensive review.^582^ It is worth noting that changes in the microbiota that occur at each stage of development, from infancy to adolescence, adulthood, and old age, may show different perturbations. The presence of homeostatic reprogramming phenomena may require the correction of some relevant indicators used to assess an “individual microbial coefficient” due to potential individual variations. For a long time, our understanding of the holistic impact of microbial dysbiosis on human homeostatic equilibrium was limited. In light of this, we propose here a conceptual model (cell-microbe homoecology and co-homeostasis) elucidating why “microbial dysbiosis” influences “cellular homeostasis” (Fig. 6b). Building upon this model, commensal microbiota emerges as a additional regulatory force in maintaining homeostasis, in addition to the well-established roles of the nervous, immune, and metabolic systems. This foundational understanding lays groundwork for the establishment of a model to investigate the transformation between health and disease in the following discussion.

## Physiological dependence and internal competition, Health and disease

Moderate exposure of the human body to commensal microbiota is essential for maintaining the development and physiological functions of nervous, immune, and metabolic systems. The absence of commensal microbiota poses a potential risk to human health. However, to maintain healthy homeostasis, the body must continually counteract the potential damage caused by the dominant microbiota. While our preceding discourse included examples like *Staphylococcus aureus* and *Candida albicans* as conditional pathogens, it is imperative to recognize that, in a rigorous sense, all human microbes exhibit conditional pathogenicity. Essentially, real “mutualism” may not exist.

### Controllable microbial community

Excessive proliferation of the microbiota at specific anatomical sites, such as small intestinal bacterial overgrowth (SIBO), can cause symptoms such as nausea, bloating, vomiting and abdominal pain, which may be caused by abnormalities in the structure or motility of the intestinal tract.^583^ In specific cases, the intervention of antibiotics also indirectly promotes the growth of specific microorganisms. For example, meropenem treatment reduced *Clostridiaceae* and promoted colonization and expansion of *Bacillus polymorphus* (BT) in the intestinal mucus layer.^584^

### Intact microbial barrier

An intact barrier function is also a strategy to prevent microbiota translocation and its impact on distant tissues or organs.^585^ The Microbiota and their components can enter the circulation and cause chronic inflammation, endotoxemia and multiple organ failure to varying degrees.^586–588^ In mice with TET2 gene deficiency, microbial signals are key drivers of pre-leukemic myeloproliferation (PMP), inducing intestinal barrier dysfunction and systemic inflammation, particularly by increasing the production of interleukin-6 (IL-6).^589^ This development of PMP can be effectively reversed or prevented through antibiotic treatment and germ-free conditions.^589^ Additionally, mutations in the CRB1 gene compromise the barrier functions of retinal and colonic epithelial cells, leading to a disruption of critical intercellular junctions.^590^ Such impairment allows specific gut bacteria to translocate across the intestinal epithelial barrier, enter the bloodstream and eventually reach the retina, where they trigger a localized inflammatory response.^590^ This activation of immune cells causes retinal cell damage, culminating in retinal degeneration, and emphasizes the importance of an intact intestinal barrier in averting both systemic and localized pathological consequences.^590^ Other disease conditions, particularly diabetes and obesity, have been also associated with the isolation of bacteria from patients’ adipose tissue.^591,592^ In addition, the major structure of the outer membrane of gram-negative rods, LPS, has been shown to be involved in the mechanisms of cardiometabolic disease, obesity and insulin resistance, cognitive dysfunction, depression, ageing and many other diseases.^593–596^

### Resist damage from microbial genetic mutations

It is important to note that commensal microbiota exhibit “adaptive” properties to the humans across lifecycle stages. Different strains of microbiota colonizing the same host show different pathogenic characteristics. For example, the *Enterococcus gallinarum* strain in the intestinal lumen can be controlled by the host immune system, whereas the strain residing in the intestinal mucosal wall niche can translocate to the mesenteric lymph nodes and liver, causing inflammation that may be associated with specific gene mutations, changes in gene expression programs, and even the remodeling of cell wall structures.^597^ This similar divergent evolutionary pattern was also observed in *Lactobacillus reuteri*, suggesting that recognition of microbiota pathogenicity should be specific at the strain level.^597^

### Reducing exposure to harmful metabolites

Although the human body can benefit from a variety of microbial products (as discussed above), harmful microbial products can also induce or exacerbate disease. Specifically, dietary substances such as choline, phosphatidylcholine (lecithin) and carnitine can be metabolized by the gut microbiota to trimethylamine (TMA). Physiological concentrations of TMA can damage tight junctions and increase permeability of the blood-brain barrier (BBB).^598^ TMA is converted to oxidized trimethylamine (TMAO) by flavin-containing monooxygenase 3 (Fmo3) in the liver, which can promote macrophage accumulation in the vascular wall, inhibit cholesterol recycling, increase platelet aggregation activity, activate the endoplasmic reticulum, and activate endoplasmic reticulum stress and cell apoptosis pathways in vascular smooth muscle cells; TMAO is closely associated with the onset and progression of several cardiovascular diseases, including atherosclerosis, heart failure and abdominal aortic aneurysm.^599–601^ However, research has unveiled the intriguing potential of TMAO to augment the therapeutic efficacy of immunotherapy, particularly for the treatment of triple-negative breast cancer and pancreatic cancer.^602,603^ These findings underscore the importance of context-specific considerations when assessing the beneficial or deleterious impact of microbial products.^602,603^ Although GF mice display anxiety-like behaviors (see discusstion in the section on the germ-free syndrome), certain gut bacteria strains, such as *Bacteroides ovatus*, metabolize dietary tyrosine into p-coumaric acid via the enzyme tyrosine ammonia lyase (encoded by BACOVA_01194).^604^ The p-coumaric acid is then converted to 4-vinylphenol (4VP) by phenolic acid decarboxylase, and subsequently reduced to 4-ethylphenol (4EP) by vinyl phenol reductase.^604^ The host’s sulfotransferase SULT1A1 sulfates 4EP to produce 4-ethylphenyl sulfate (4EPS).^604^ Upon entering the brain, 4EPS induces changes in the activity and functional connectivity of specific brain regions, impairs the maturation of oligodendrocytes and reduces their interaction with neurons, can also leading to the manifestation of anxiety-like behaviors in the mice.^604^ In female patients with polycystic ovary syndrome, an increase in the gut microbiota *Bacteroides vulgatus* is associated with the characteristic manifestations of the syndrome, including excessive androgen levels, ovulatory dysfunction, and polycystic ovary morphology.^605^ On one hand, *B. vulgatus*, through its metabolic activities—particularly via the action of bile salt hydrolase enzymes—reduces the levels of glycodeoxycholic acid (GDCA) and tauroursodeoxycholic acid (TUDCA) in the gut. These bile acids are potent agonists of the farnesoid X receptor (FXR), and their decrease leads to weakened FXR signaling, which may in turn suppress the production of IL-22, a cytokine crucial for gut barrier and immune function, associated with the development of PCOS.^605^ On the other hand, agmatine produced by *B. vulgatus* from dietary arginine through the action of arginine decarboxylase acts as an endogenous agonist of FXR.^606^ The activation of FXR in intestinal L cells inhibits the expression of the proglucagon gene, which encodes the precursor of glucagon-like peptide-1 (GLP-1), decreased secretion of GLP-1 in response to glucose contributing to insulin resistance in PCOS.^607^ Similarly, Imidazole propionate (ImP) is a microbial metabolite produced from histidine that may regulate host inflammation to promote insulin resistance and is significantly elevated in the serum of diabetic patients.^608,609^ Three branched-chain amino acids (BCAAs), leucine, isoleucine and valine, synthesized by *Prevotella copri* and *Bacteroides vulgatus*, also increase the risk of diabetes.^610^ porA and fldH genes of the gut microbiota mediate the conversion of dietary phenylalanine to phenylacetic acid and phenylpropionic acid, respectively.^611^ The former can be synthesized into phenylacetylglutamine (PAGln), which enhances platelet activation and thrombogenic potential.^611^ In addition to metabolic molecules, microbial peptides can disrupt the body. For example, *E. coli* secretes ClpB, a melanocyte-stimulating hormone (α-MSH) analogue that can cause anxiety, anorexia and eating disorders.^612^ Similarly, the genus *Bacteroides* produces a myosin heavy chain 6 (MYH6) mimetic peptide, β-galactosidase, which can induce T-cell attack on the heart, causing fatal inflammatory cardiomyopathy.^613^ Recent research has identified several novel enzymes in the gut microbiota that have similar functions to those found in the host (Microbial-host-isozyme).^614^ Among these, the bacterial isoenzyme of the key diabetes target, dipeptidyl peptidase-4 (DPP4), can reduce the activity of endogenous GLP-1 in a mouse model of impaired intestinal barrier function which negatively affects glucose homeostasis.^614^ Notably, microorganisms can secrete exopolysaccharides (EPS) that cloak the LPS on their surface, which are typically recognized by the human immune system.^615^ By doing so, they diminish the activation of the hypothalamic acute stress response mediated by TLR4-TRPV1+ sensory neurons in the lungs, effectively sidestepping the body’s defense mechanisms against infection.^615^

**Fig. 7:**
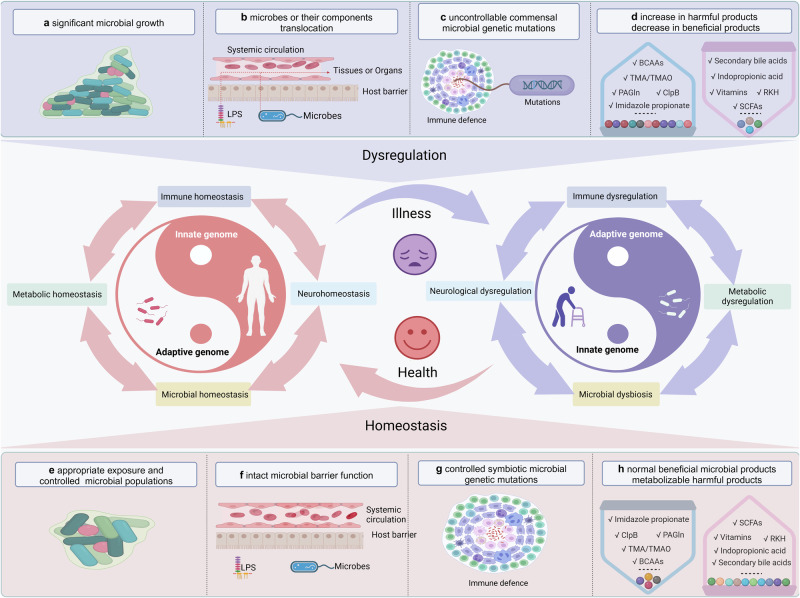
The model of the interplay between the human innate and adaptive genome in health and disease transformation. The human innate and adaptive genomes form a holistic functional phenotype but are also in constant competition. The regulation of the nervous, immune, metabolic, and commensal microbiota constitutes the four major features and regulatory forces of human health and disease states. These forces interact with each other, and microbial dysbiosis can lead to the disruption of other forces and vice versa. The innate genome requires (**a**) the necessary exposure to commensal microbiota and a controllable microbial community, (**b**) an intact microbial barrier, (**c**) the ability to resist damage from microbial genetic mutations, and (**d**) the ability to utilize beneficial products of the adaptive genome and metabolize harmful ones to maintain a healthy steady state (with the innate genome in the dominant position). Conversely, (**e**) inadequate exposure to appropriate microbiota (which can lead to germ-free syndrome in extreme cases) or microbial overgrowth, (**f**) microbes or their components (e.g. LPS) entering the circulation through an incomplete barrier and causing harm to other tissues and organs, (**g**) microbial genetic mutations causing additional damage, and (**h**) a decrease in beneficial microbial products and an increase in harmful ones can lead humans towards disease progression (with the adaptive genome dominant). LPS Lipopolysaccharides, TMA Trimethylamine, TMAO Trimethylamine N-oxide, PAGln Phenylacetylglutamine, ClpB a melanocyte-stimulating hormone (α-MSH) analogue, BCAAs Branched-chain amino acids, SCFAs Short-chain fatty acids, RKH Arginyl-lysyl-histidine

In summary, in the long-term co-evolution with microbes, both humans and microbes have developed various adaptive mechanisms that influence each other. Understanding human dependence and internal competition with commensal microbes is key to understanding the transition between health and disease, homeostasis and dysregulation (Fig. 7). It is known that co-inhabitants can share 12% gut and 32% oral microbiota strains, so as a special point, certain non-communicable diseases such as cardiovascular diseases, diabetes, and inflammatory bowel diseases maybe predicted transmitted within human communities via human microbiota as intermediaries, becoming atypical infectious diseases.^616,617^ This gives rise to another concept: the social microbiome, which encapsulates the intricate interplay of microbial communities across a host’s social fabric, shaping the landscape of health and disease.^618^

## Conclusion

The fusion of human sperm and egg creates our innate genome, while the microbiome, with its random and non-heritable nature, evolves as an adaptive genome. This adaptive genome is dynamic and personalized, constantly adapting to our physiology, pathology, environment, diet and microbial interactions. The innate genome and adaptive microbiomes are intertwined, resulting in the reprogramming of the organism’s homeostasis. Loss of interaction with the adaptive genome is likely to result in germ-free syndromes (hypotheses based on germ-free animals). From a histological perspective, the human microbiota can be viewed as ‘slave tissues’ managed by the epithelial, connective, muscular and nervous tissues that have evolved from the inherent genome. The incorporation of slave tissue allows for an extension of the body’s immune capabilities, providing an additional form of defence and immune modulation. When interacting with the external environment, understanding the host and its microbiome as a unified entity, or ‘meta-host’, may partially explain the heterogeneity in disease susceptibility, pathogenicity, severity and varying success rates of organ transplantation. When examining the internal relationship between the human body and its microbiota, it’s understood that human tissues reap the potential benefits provided by the microbiome, while at the same time using various mechanisms to regulate and minimize the potential harms or costs associated with it. The homeostasis and dysbiosis of microorganisms with the human neural, metabolic and immune systems are the causal driving force behind health and disease outcomes.

**Fig. 8:**
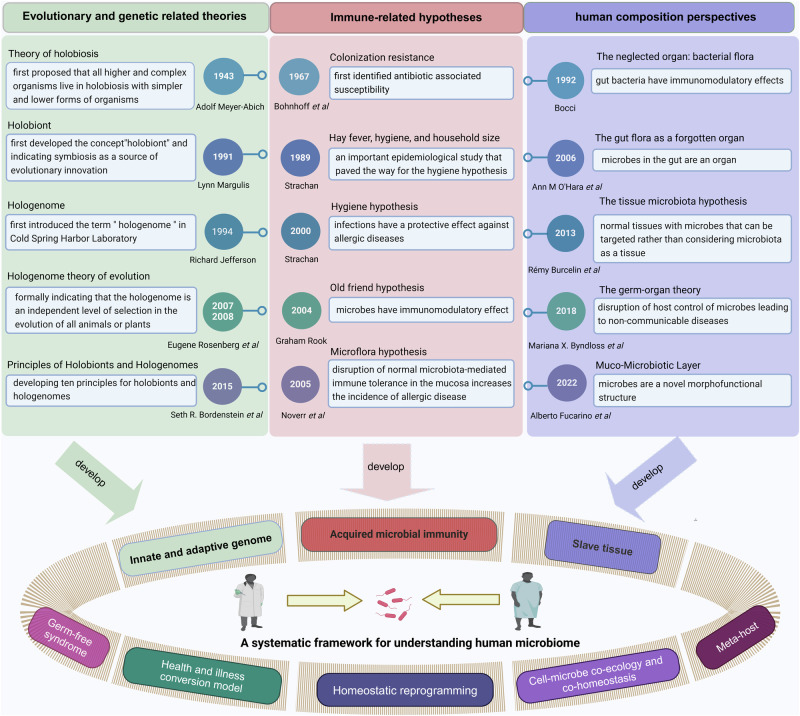
A systematic framework for understanding human microbes and the history of the development of some of these concepts. The systematic framework consists of eight fundamental concepts/models: “innate genome and adaptive genome”, “slave tissue”, “acquired microbial immunity”, “cell-microbe co-ecology and co-homeostasis model”, “meta-host model”, “health and illness transformation model” and “germ-free syndrome”

Within the theoretical framework discussed above and diagrammatically presented in Fig. 8, the “germ-free syndrome” highlights the need to shift from the traditional view of “microbes as pathogens” to the understanding that “a lack of microbes can also be detrimental to health”. The “Innate and adaptive genome” improves our understanding at the genetic and evolutionary level of the complete human genome, detailing the essential characteristics of the adaptive genome. The concept of “slave tissue” integrates ecological and human tissue perspectives, illustrating the intricate relationship between multicellular organisms and their associated microbiome. It elucidates how human master tissues can both reap benefits and endure adversities due to the presence of microbes. This concept redirects focus towards the alterations in ‘slave tissue’ as the disease progresses, emphasizing the dynamic interplay between host and microbe in health and disease. The “Acquired microbial immunity” unifies the roles of colonization resistance and immune regulation, considering the microbiome as a source of supplementary power to human defences. This concept provides a theoretical foundation for combating antibiotic misuse and for utilizing microecological therapies in the treatment of allergic and inflammatory diseases. The “Homeostasis reprogramming hypothesis” complements the foundations of modern medicine, represented by the “internal environment theory”, by bridging the conceptual gap left by the neglect of the microbiome’s role. This may in part explain the trend observed since the industrialization of a decline in some of the body’s homeostatic indicators, such as basal body temperature and changes in blood glucose levels, in association with the body’s microbial diversity. The “Cell-microbe co-ecology model” demonstrate the close association between “microbial regulation” and “cell homeostasis,” offering a necessary understanding of why microbial dysregulation can impact homeostatic balance in humans. The “meta-host model” extends the definition of host. It suggests that symbiotic microorganisms act as co-hosts within the human ecological environment. The “Health-illness conversion model” elucidates the dual relationship between the innate and adaptive genomes as a whole and their internal competition. It summarizes four patterns of microbial dysregulation within the human body.

## Future perspectives

The recent release of the global burden report highlights the daunting challenges we face in various public health crises, including obesity and malnutrition,^619^ cardiovascular diseases,^620^ gastrointestinal disorders,^621^ diabetes,^622^ antimicrobial resistance^197^ and cancers.^623^ The outbreak of the novel coronavirus has further compelled us to reflect on how we can enhance our prevention and treatment strategies for potential future infectious disease outbreaks.^624^ Microbiome, as the human adative genome, present a promising avenue for potential breakthroughs in this regard. Although progress has been made, the secrets of the relationship between humans and microbes have not been fully unlocked. More research is needed in the future to glimpse what lies beneath the tip of the iceberg. Some of the directions include but are not limited to the following:

Protecting the diversity of human and environmental microbiomes,^625^ avoiding the gradual loss of adaptive genomic elements, and establishing and maintaining microbiome banks are possible strategies that depend on interdisciplinary and international collaborations. These proactive measures have the potential to provide substantial benefits to diverse organisms, including augmented crop yields and bolstered resilience of plants in the face of climate change^626,627^ and shows potential in protecting endangered animal species.^628^

Further deciphering the interaction mechanisms between the microbiota and other organs and tissues is essential. Currently, microbiota research on the following:

The effects of other tissue developmental stages are insufficient;

Viruses,^496,629–636^ fungi,^637,638^ archaea^639–642^ have received less attention than bacteria;

Non-gut microbiomes are understudied;

Communication mechanisms between microbiomes from different body sites, such as interaction between the gut and the lung/skin/oral, are not well understood;

Tumor tissues disrupt body homeostasis, allowing microbes to colonize the tumor environment through damaged tissues and bloodstream.^643–645^ These microbes promote tumor development by inducing mutations, affecting gene regulation, promoting inflammation, evading the immune system, and enhancing metastasis.^646^ Their interaction offers an additional opportunity for targeted cancer interventions;^647^

Specific populations, such as rare disease patients, surgical patients, and transplant recipients, have not been well considered.

The causal relationship between microbes and diseases is still not well revealed.

The clinical applications of microbiomes mainly include diseases diagnosis (biomarkers), classification (severity), treatment (gene editing), and prognosis assessment.

Adaptive genomic elements and their effects on diet and drug responses must not be overlooked, as they have significant impacts on human physiology and therapy. Further characterization is required.

The utilization of “acquired microbial immunity” could offer additional therapeutic options for allergies, autoimmune diseases, and enteric infections, but potential risks should be carefully assessed with consideration of host conditions.

Standardized clinical guidelines are prerequisites for clinical translation. “Microbial clinical specialists” and “microbial clinical department” are potential forms for future implementation.

The field of microbiome engineering is advancing with precision and complexity, employing a range of genetic strategies to manipulate the microbial ecosystem for therapeutic benefit.^648^ Engineered bacteria, such as *E. coli* Nissle 1917 strain, SYNB1020, effectively converts ammonia in the gut to L-arginine, ameliorating hyperammonemia and boosting survival rates in mice.^649^ By meticulously tuning key elements of gene expression, researchers have successfully achieved efficient biosynthesis of important compounds such as β-carotene and violacein in *Saccharomyces boulardii*.^650^ In an innovative approach to addressing *C. difficile* infections, researchers have developed a recombinant bacteriophage that expresses CRISPR RNAs to guide the native Cas3 protein in targeting and degrading the pathogen’s chromosomal DNA, leading to the bacterial destruction.^651^ Programmable exogenous phage-delivered CRISPR/Cas9 delivery demonstrates the feasibility of strain-specific gene knockout and chromosomal deletion in complex microbial communities.^652^ For example, utilizing engineered M13 bacteriophages as vectors to specifically deliver the CRISPR-Cas9 system to *E. coli* within the mouse gut, enabling precise genetic editing at targeted loci despite the need to address challenges such as low bacteriophage viability and bacterial evasion of editing.^652^ Additionally, synthetic genetic elements developed through computational design enable the re-engineering of biosynthetic gene clusters for expression in various hosts.^653^ The innovative concept of microbial swarmbots encapsulates the synergy of multiple engineered microbes within microcapsules, working collectively to perform high-throughput functions.^654^

In the realm of microbiome research, equity is a critical issue.^655^ Studies from developing or impoverished nations are significantly disadvantaged and underrepresented compared to those from developed countries. In this regard, international professional associations, relevant governmental and societal research funding bodies, and academic journals should consider policy inclinations towards regions or research that are underrepresented. In areas or countries where it is challenging to organize large-scale population studies, the success of citizen science methods adopted by Belgium^118^ and the recent proposal of the African Equitable Scheme by Ovokeraye H. Oduaran and colleagues are worthy of emulation.^656^

We are presently on the trajectory of comprehending natural phenomena, deciphering intricate mechanisms, and harnessing the potential of microbes to optimize human health. In the end, if it is truly possible for humans to colonize other planets, focusing merely on our innate genome while ignoring our adaptive genome could lead to wider health issues. Consequently, in all conceivable scenarios, contemplating an interplanetary microbiome initiative becomes an inevitable necessity.

## References

[1] Nurk, S. et al. The complete sequence of a human genome. *Science* **376**, 44–53 (2022). 35357919 10.1126/science.abj6987 PMC9186530

[2] Vollger, M. R. et al. Segmental duplications and their variation in a complete human genome. *Science* **376**, eabj6965 (2022). 35357917 10.1126/science.abj6965 PMC8979283

[3] Hoyt, S. J. et al. From telomere to telomere: the transcriptional and epigenetic state of human repeat elements. *Science* **376**, eabk3112 (2022). 35357925 10.1126/science.abk3112 PMC9301658

[4] Gershman, A. et al. Epigenetic patterns in a complete human genome. *Science* **376**, eabj5089 (2022). 35357915 10.1126/science.abj5089 PMC9170183

[5] Altemose, N. et al. Complete genomic and epigenetic maps of human centromeres. *Science* **376**, eabl4178 (2022). 35357911 10.1126/science.abl4178 PMC9233505

[6] Aganezov, S. et al. A complete reference genome improves analysis of human genetic variation. *Science* **376**, eabl3533 (2022). 35357935 10.1126/science.abl3533 PMC9336181

[7] Liao, W.-W. et al. A draft human pangenome reference. *Nature* **617**, 312–324 (2023). 37165242 10.1038/s41586-023-05896-x PMC10172123

[8] Gao, Y. et al. A pangenome reference of 36 Chinese populations. *Nature* **619**, 112–121 (2023). 10.1038/s41586-023-06173-7 PMC10322713 37316654

[9] Wang, T. et al. The Human Pangenome Project: a global resource to map genomic diversity. *Nature* **604**, 437–446 (2022). 35444317 10.1038/s41586-022-04601-8 PMC9402379

[10] Huang, T., Shu, Y. & Cai, Y.-D. Genetic differences among ethnic groups. *BMC Genomics* **16**, 1093 (2015). 26690364 10.1186/s12864-015-2328-0 PMC4687076

[11] Gest, H. The discovery of microorganisms by Robert Hooke and Antoni van Leeuwenhoek, Fellows of The Royal Society. *Notes Rec. R. Soc. Lond.* **58**, 187–201 (2004). 15209075 10.1098/rsnr.2004.0055

[12] The Integrative HMP (iHMP) Research Network Consortium. The Integrative Human Microbiome Project. *Nature* **569**, 641–648 (2019). 31142853 10.1038/s41586-019-1238-8 PMC6784865

[13] Lloyd-Price, J. et al. Strains, functions and dynamics in the expanded Human Microbiome Project. *Nature* **550**, 61–66 (2017). 28953883 10.1038/nature23889 PMC5831082

[14] Integrative HMP (iHMP) Research Network Consortium. The Integrative Human Microbiome Project: dynamic analysis of microbiome-host omics profiles during periods of human health and disease. *Cell Host Microbe* **16**, 276–289 (2014). 10.1016/j.chom.2014.08.014 PMC5109542 25211071

[15] The Human Microbiome Project Consortium. Structure, function and diversity of the healthy human microbiome. *Nature* **486**, 207–214 (2012). 22699609 10.1038/nature11234 PMC3564958

[16] MetaHIT Consortium. et al. A human gut microbial gene catalogue established by metagenomic sequencing. *Nature* **464**, 59–65 (2010). 20203603 10.1038/nature08821 PMC3779803

[17] Le Chatelier, E. et al. Richness of human gut microbiome correlates with metabolic markers. *Nature* **500**, 541–546 (2013). 23985870 10.1038/nature12506

[18] MetaHIT Consortium. et al. An integrated catalog of reference genes in the human gut microbiome. *Nat. Biotechnol.* **32**, 834–841 (2014). 24997786 10.1038/nbt.2942

[19] MetaHIT Consortium (additional members). et al. Enterotypes of the human gut microbiome. Nature 473, 174–180 (2011). 10.1038/nature09944 PMC3728647 21508958

[20] MetaHIT consortium. et al. Disentangling type 2 diabetes and metformin treatment signatures in the human gut microbiota. *Nature* **528**, 262–266 (2015). 26633628 10.1038/nature15766 PMC4681099

[21] McDonald, D. et al. American gut: an open platform for citizen science microbiome research. *mSystems* **3**, e00031–18 (2018). 29795809 10.1128/mSystems.00031-18 PMC5954204

[22] Lopera-Maya, E. A. et al. Effect of host genetics on the gut microbiome in 7,738 participants of the Dutch Microbiome Project. *Nat. Genet.* **54**, 143–151 (2022). 35115690 10.1038/s41588-021-00992-y

[23] López-Otín, C. & Kroemer, G. Hallmarks of Health. *Cell* **184**, 33–63 (2021). 33340459 10.1016/j.cell.2020.11.034

[24] Schmauck-Medina, T. et al. New hallmarks of ageing: a 2022 Copenhagen ageing meeting summary. *Aging* **14**, 6829–6839 (2022). 36040386 10.18632/aging.204248 PMC9467401

[25] López-Otín, C., Blasco, M. A., Partridge, L., Serrano, M. & Kroemer, G. Hallmarks of aging: an expanding universe. *Cell* **186**, 243–278 (2023). 36599349 10.1016/j.cell.2022.11.001

[26] Nicholson, J. K. et al. Host-gut microbiota metabolic interactions. *Science* **336**, 1262–1267 (2012). 22674330 10.1126/science.1223813

[27] Cho, I. & Blaser, M. J. The human microbiome: at the interface of health and disease. *Nat. Rev. Genet.* **13**, 260–270 (2012). 22411464 10.1038/nrg3182 PMC3418802

[28] Gilbert, J. A. et al. Current understanding of the human microbiome. *Nat. Med.* **24**, 392–400 (2018). 29634682 10.1038/nm.4517 PMC7043356

[29] Rackaityte, E. & Lynch, S. V. The human microbiome in the 21st century. *Nat. Commun.* **11**, 5256 (2020). 33067429 10.1038/s41467-020-18983-8 PMC7567807

[30] Cani, P. D. Human gut microbiome: hopes, threats and promises. *Gut* **67**, 1716–1725 (2018). 29934437 10.1136/gutjnl-2018-316723 PMC6109275

[31] de Vos, W. M., Tilg, H., Van Hul, M. & Cani, P. D. Gut microbiome and health: mechanistic insights. *Gut* **71**, 1020–1032 (2022). 35105664 10.1136/gutjnl-2021-326789 PMC8995832

[32] Clemente, J. C., Ursell, L. K., Parfrey, L. W. & Knight, R. The impact of the gut microbiota on human health: an integrative view. *Cell* **148**, 1258–1270 (2012). 22424233 10.1016/j.cell.2012.01.035 PMC5050011

[33] Brodin, P. Immune-microbe interactions early in life: a determinant of health and disease long term. *Science* **376**, 945–950 (2022). 35617387 10.1126/science.abk2189

[34] McFall-Ngai, M. et al. Animals in a bacterial world, a new imperative for the life sciences. *Proc. Natl Acad. Sci.* **110**, 3229–3236 (2013). 23391737 10.1073/pnas.1218525110 PMC3587249

[35] Valdes, A. M., Walter, J., Segal, E. & Spector, T. D. Role of the gut microbiota in nutrition and health. *BMJ* **361**, k2179 (2018). 10.1136/bmj.k2179 PMC6000740 29899036

[36] Lynch, S. V. & Pedersen, O. The human intestinal microbiome in health and disease. *N. Engl. J. Med.* **375**, 2369–2379 (2016). 27974040 10.1056/NEJMra1600266

[37] Martino, C. et al. Microbiota succession throughout life from the cradle to the grave. *Nat. Rev. Microbiol.* **20**, 707–720 (2022). 35906422 10.1038/s41579-022-00768-z PMC12875531

[38] Gilbert, J. A. et al. Microbiome-wide association studies link dynamic microbial consortia to disease. *Nature* **535**, 94–103 (2016). 27383984 10.1038/nature18850

[39] Ayres, J. S. Cooperative microbial tolerance behaviors in host-microbiota mutualism. *Cell* **165**, 1323–1331 (2016). 27259146 10.1016/j.cell.2016.05.049 PMC4903080

[40] Clemente, J. C., Manasson, J. & Scher, J. U. The role of the gut microbiome in systemic inflammatory disease. *BMJ* **360**, j5145 (2018). 29311119 10.1136/bmj.j5145 PMC6889978

[41] Lee, J.-Y., Tsolis, R. M. & Bäumler, A. J. The microbiome and gut homeostasis. *Science* **377**, eabp9960 (2022). 35771903 10.1126/science.abp9960

[42] Schmidt, T. S. B., Raes, J. & Bork, P. The human gut microbiome: from association to modulation. *Cell* **172**, 1198–1215 (2018). 29522742 10.1016/j.cell.2018.02.044

[43] Bäckhed, F., Ley, R. E., Sonnenburg, J. L., Peterson, D. A. & Gordon, J. I. Host-bacterial mutualism in the human intestine. *Science* **307**, 1915–1920 (2005). 15790844 10.1126/science.1104816

[44] Bordenstein, S. R. & Theis, K. R. Host biology in light of the microbiome: ten principles of holobionts and hologenomes. *PLoS Biol.* **13**, e1002226 (2015). 26284777 10.1371/journal.pbio.1002226 PMC4540581

[45] The Superorganism. in *Biophilia* 23–38 (Harvard University Press, 1984). 10.4159/9780674045231-003.

[46] Bell, G. Model metaorganism. *Science* **282**, 248–248 (1998).

[47] Theis, K. R. et al. Getting the hologenome concept right: an eco-evolutionary framework for hosts and their microbiomes. *mSystems* **1**, e00028–16 (2016). 27822520 10.1128/mSystems.00028-16 PMC5069740

[48] Brown, J. M. & Hazen, S. L. The gut microbial endocrine organ: bacterially derived signals driving cardiometabolic diseases. *Annu. Rev. Med.* **66**, 343–359 (2015). 25587655 10.1146/annurev-med-060513-093205 PMC4456003

[49] Bach, J.-F. The hygiene hypothesis in autoimmunity: the role of pathogens and commensals. *Nat. Rev. Immunol.* **18**, 105–120 (2018). 29034905 10.1038/nri.2017.111

[50] Rook, G. A. W. A Darwinian View of the Hygiene or “Old Friends” Hypothesis: When urban living reduced contacts of humans with microbes and worms, it increased our risk for chronic inflammatory disorders. *Microbe Mag.* **7**, 173–180 (2012).

[51] Noverr, M. C. & Huffnagle, G. B. The ‘microflora hypothesis’ of allergic diseases. *Clin. Exp. Allergy* **35**, 1511–1520 (2005). 16393316 10.1111/j.1365-2222.2005.02379.x

[52] Quagliariello, A. et al. Ancient oral microbiomes support gradual Neolithic dietary shifts towards agriculture. *Nat. Commun.* **13**, 6927 (2022). 36414613 10.1038/s41467-022-34416-0 PMC9681849

[53] Suzuki, T. A. et al. Codiversification of gut microbiota with humans. *Science* **377**, 1328–1332 (2022). 36108023 10.1126/science.abm7759 PMC10777373

[54] Moeller, A. H. et al. Cospeciation of gut microbiota with hominids. *Science* **353**, 380–382 (2016). 27463672 10.1126/science.aaf3951 PMC4995445

[55] Rook, G., Bäckhed, F., Levin, B. R., McFall-Ngai, M. J. & McLean, A. R. Evolution, human-microbe interactions, and life history plasticity. *Lancet* **390**, 521–530 (2017). 28792414 10.1016/S0140-6736(17)30566-4

[56] Sharp, C. & Foster, K. R. Host control and the evolution of cooperation in host microbiomes. *Nat. Commun.* **13**, 3567 (2022). 35732630 10.1038/s41467-022-30971-8 PMC9218092

[57] Hector, T. E., Hoang, K. L., Li, J. & King, K. C. Symbiosis and host responses to heating. *Trends Ecol. Evol.* **37**, 611–624 (2022). 35491290 10.1016/j.tree.2022.03.011

[58] Frazão, N. et al. Two modes of evolution shape bacterial strain diversity in the mammalian gut for thousands of generations. *Nat. Commun.* **13**, 5604 (2022). 36153389 10.1038/s41467-022-33412-8 PMC9509342

[59] Ley, R. E. et al. Evolution of mammals and their gut microbes. *Science* **320**, 1647–1651 (2008). 18497261 10.1126/science.1155725 PMC2649005

[60] Davenport, E. R. et al. The human microbiome in evolution. *BMC Biol.* **15**, 127 (2017). 29282061 10.1186/s12915-017-0454-7 PMC5744394

[61] Barreto, H. C. & Gordo, I. Intrahost evolution of the gut microbiota. *Nat. Rev. Microbiol*. **21**, 590–603 (2023). 10.1038/s41579-023-00890-6 37069454

[62] The NIH HMP Working Group. et al. The NIH Human Microbiome Project. *Genome Res.* **19**, 2317–2323 (2009). 19819907 10.1101/gr.096651.109 PMC2792171

[63] Koren, O. et al. Human oral, gut, and plaque microbiota in patients with atherosclerosis. *Proc. Natl Acad. Sci.* **108**, 4592–4598 (2011). 20937873 10.1073/pnas.1011383107 PMC3063583

[64] Clifford, A. & Hoffman, G. S. Evidence for a vascular microbiome and its role in vessel health and disease. *Curr. Opin. Rheumatol.* **27**, 397–405 (2015). 26002032 10.1097/BOR.0000000000000184

[65] Hidi, L. et al. Human blood vessel microbiota in healthy adults based on common femoral arteries of brain-dead multi-organ donors. *Front. Cell. Infect. Microbiol.* **12**, 1056319 (2022). 36530429 10.3389/fcimb.2022.1056319 PMC9747773

[66] Tan, C. C. S. et al. No evidence for a common blood microbiome based on a population study of 9770 healthy humans. *Nat. Microbiol.* **8**, 973–985 (2023). 36997797 10.1038/s41564-023-01350-w PMC10159858

[67] Lu, L. J. & Liu, J. Human microbiota and ophthalmic disease. *Yale J. Biol. Med.* **89**, 325–330 (2016). 27698616 PMC5045141

[68] Ozkan, J. et al. Biogeography of the human ocular microbiota. *Ocul. Surf.* **17**, 111–118 (2019). 30445178 10.1016/j.jtos.2018.11.005

[69] Lyon, J. Even the eye has a microbiome. *JAMA* **318**, 689 (2017). 28829856 10.1001/jama.2017.10599

[70] St. Leger, A. J. et al. An ocular commensal protects against corneal infection by driving an interleukin-17 response from mucosal γδ t cells. *Immunity* **47**, 148–158.e5 (2017). 28709803 10.1016/j.immuni.2017.06.014 PMC5553552

[71] Castellani, G., Croese, T., Peralta Ramos, J. M. & Schwartz, M. Transforming the understanding of brain immunity. *Science* **380**, eabo7649 (2023). 37023203 10.1126/science.abo7649

[72] Servick, K. Do gut bacteria make a second home in our brains? *Science*10.1126/science.aaw0147 (2018).

[73] Link, C. D. Is there a brain microbiome? *Neurosci. Insights* **16**, 263310552110187 (2021). 10.1177/26331055211018709 PMC8165828 34104888

[74] Molinero, N. et al. The human gallbladder microbiome is related to the physiological state and the biliary metabolic profile. *Microbiome* **7**, 100 (2019). 31272480 10.1186/s40168-019-0712-8 PMC6610825

[75] Urbaniak, C. et al. Microbiota of human breast tissue. *Appl. Environ. Microbiol.* **80**, 3007–3014 (2014). 24610844 10.1128/AEM.00242-14 PMC4018903

[76] De Goffau, M. C. et al. Human placenta has no microbiome but can contain potential pathogens. *Nature* **572**, 329–334 (2019). 31367035 10.1038/s41586-019-1451-5 PMC6697540

[77] Younge, N. et al. Fetal exposure to the maternal microbiota in humans and mice. *JCI Insight* **4**, e127806 (2019). 31479427 10.1172/jci.insight.127806 PMC6795398

[78] Hornef, M. & Penders, J. Does a prenatal bacterial microbiota exist? *Mucosal Immunol.* **10**, 598–601 (2017). 28120852 10.1038/mi.2016.141

[79] Mishra, A. et al. Microbial exposure during early human development primes fetal immune cells. *Cell* **184**, 3394–3409.e20 (2021). 34077752 10.1016/j.cell.2021.04.039 PMC8240556

[80] Perez-Muñoz, M. E., Arrieta, M.-C., Ramer-Tait, A. E. & Walter, J. A critical assessment of the “sterile womb” and “in utero colonization” hypotheses: implications for research on the pioneer infant microbiome. *Microbiome* **5**, 48 (2017). 28454555 10.1186/s40168-017-0268-4 PMC5410102

[81] Kennedy, K. M. et al. Fetal meconium does not have a detectable microbiota before birth. *Nat. Microbiol.* **6**, 865–873 (2021). 33972766 10.1038/s41564-021-00904-0

[82] Kennedy, K. M. et al. Questioning the fetal microbiome illustrates pitfalls of low-biomass microbial studies. *Nature* **613**, 639–649 (2023). 36697862 10.1038/s41586-022-05546-8 PMC11333990

[83] Dominguez-Bello, M. G. et al. Delivery mode shapes the acquisition and structure of the initial microbiota across multiple body habitats in newborns. *Proc. Natl Acad. Sci.* **107**, 11971–11975 (2010). 20566857 10.1073/pnas.1002601107 PMC2900693

[84] Faith, J. J. et al. The long-term stability of the human gut microbiota. *Science* **341**, 1237439 (2013). 23828941 10.1126/science.1237439 PMC3791589

[85] Liang, G. & Bushman, F. D. The human virome: assembly, composition and host interactions. *Nat. Rev. Microbiol.* **19**, 514–527 (2021). 33785903 10.1038/s41579-021-00536-5 PMC8008777

[86] McCallum, G. & Tropini, C. The gut microbiota and its biogeography. *Nat. Rev. Microbiol.* **22**, 105–118 (2024). 37740073 10.1038/s41579-023-00969-0

[87] Yassour, M. et al. Natural history of the infant gut microbiome and impact of antibiotic treatment on bacterial strain diversity and stability. *Sci. Transl. Med*. **8** (2016). 10.1126/scitranslmed.aad0917 PMC5032909 27306663

[88] Odamaki, T. et al. Age-related changes in gut microbiota composition from newborn to centenarian: a cross-sectional study. *BMC Microbiol.* **16**, 90 (2016). 27220822 10.1186/s12866-016-0708-5 PMC4879732

[89] Folz, J. et al. Human metabolome variation along the upper intestinal tract. *Nat. Metab.* **5**, 777–788 (2023). 37165176 10.1038/s42255-023-00777-z PMC10229427

[90] Shalon, D. et al. Profiling the human intestinal environment under physiological conditions. *Nature* **617**, 581–591 (2023). 37165188 10.1038/s41586-023-05989-7 PMC10191855

[91] She, J.-J. et al. Defining the biogeographical map and potential bacterial translocation of microbiome in human ‘surface organs’. *Nat. Commun.* **15**, 427 (2024). 38199995 10.1038/s41467-024-44720-6 PMC10781665

[92] Mason, M. R., Chambers, S., Dabdoub, S. M., Thikkurissy, S. & Kumar, P. S. Characterizing oral microbial communities across dentition states and colonization niches. *Microbiome* **6**, 67 (2018). 29631628 10.1186/s40168-018-0443-2 PMC5891995

[93] Sampaio-Maia, B. & Monteiro-Silva, F. Acquisition and maturation of oral microbiome throughout childhood: an update. *Dent. Res. J.* **11**, 291–301 (2014). PMC4119360 25097637

[94] Dzidic, M. et al. Oral microbiome development during childhood: an ecological succession influenced by postnatal factors and associated with tooth decay. *ISME J.* **12**, 2292–2306 (2018). 29899505 10.1038/s41396-018-0204-z PMC6092374

[95] Merglova, V. & Polenik, P. Early colonization of the oral cavity in 6- and 12-month-old infants by cariogenic and periodontal pathogens: a case-control study. *Folia Microbiol. (Praha)* **61**, 423–429 (2016). 26914065 10.1007/s12223-016-0453-z

[96] Crielaard, W. et al. Exploring the oral microbiota of children at various developmental stages of their dentition in the relation to their oral health. *BMC Med. Genomics* **4**, 22 (2011). 21371338 10.1186/1755-8794-4-22 PMC3058002

[97] Ruan, X., Luo, J., Zhang, P. & Howell, K. The salivary microbiome shows a high prevalence of core bacterial members yet variability across human populations. *Npj Biofilms Microbiomes* **8**, 85 (2022). 36266278 10.1038/s41522-022-00343-7 PMC9584946

[98] Gaitanis, G. et al. Variation of cultured skin microbiota in mothers and their infants during the first year postpartum. *Pediatr. Dermatol.* **36**, 460–465 (2019). 31025407 10.1111/pde.13829

[99] Chu, D. M. et al. Maturation of the infant microbiome community structure and function across multiple body sites and in relation to mode of delivery. *Nat. Med.* **23**, 314–326 (2017). 28112736 10.1038/nm.4272 PMC5345907

[100] NISC Comparative Sequencing Program. et al. Biogeography and individuality shape function in the human skin metagenome. *Nature* **514**, 59–64 (2014). 25279917 10.1038/nature13786 PMC4185404

[101] Saheb Kashaf, S. et al. Integrating cultivation and metagenomics for a multi-kingdom view of skin microbiome diversity and functions. *Nat. Microbiol.* **7**, 169–179 (2021). 34952941 10.1038/s41564-021-01011-w PMC8732310

[102] Harris-Tryon, T. A. & Grice, E. A. Microbiota and maintenance of skin barrier function. *Science* **376**, 940–945 (2022). 35617415 10.1126/science.abo0693

[103] Byrd, A. L., Belkaid, Y. & Segre, J. A. The human skin microbiome. *Nat. Rev. Microbiol.* **16**, 143–155 (2018). 29332945 10.1038/nrmicro.2017.157

[104] Yildirim, S. et al. Primate vaginal microbiomes exhibit species specificity without universal *Lactobacillus* dominance. *ISME J.* **8**, 2431–2444 (2014). 25036926 10.1038/ismej.2014.90 PMC4260710

[105] Łaniewski, P. & Herbst-Kralovetz, M. M. Connecting microbiome and menopause for healthy ageing. *Nat. Microbiol.* **7**, 354–358 (2022). 35246661 10.1038/s41564-022-01071-6 PMC9977513

[106] Reid, G. Therapeutic Opportunities in the Vaginal Microbiome. *Microbiol. Spectr.* **5**, 5.3.06 (2017). 10.1128/microbiolspec.bad-0001-2016 PMC11687489 28597813

[107] Gliniewicz, K. et al. Comparison of the Vaginal Microbiomes of Premenopausal and Postmenopausal Women. *Front. Microbiol.* **10**, 193 (2019). 30837959 10.3389/fmicb.2019.00193 PMC6382698

[108] Brotman, R. M. et al. Association between the vaginal microbiota, menopause status, and signs of vulvovaginal atrophy. *Menopause* **21**, 450–458 (2014). 24080849 10.1097/GME.0b013e3182a4690b PMC3994184

[109] Hillier, S. L. & Lau, R. J. Vaginal microflora in postmenopausal women who have not received estrogen replacement therapy. *Clin. Infect. Dis.* **25**, S123–S126 (1997). 9310650 10.1086/516221

[110] Ravel, J. et al. Vaginal microbiome of reproductive-age women. *Proc. Natl Acad. Sci. USA* **108**, 4680–4687 (2011). 20534435 10.1073/pnas.1002611107 PMC3063603

[111] Zhou, X. et al. The vaginal bacterial communities of Japanese women resemble those of women in other racial groups. *FEMS Immunol. Med. Microbiol.* **58**, 169–181 (2010). 19912342 10.1111/j.1574-695X.2009.00618.x PMC2868947

[112] Shi, Y., Chen, L., Tong, J. & Xu, C. Preliminary characterization of vaginal microbiota in healthy Chinese women using cultivation‐independent methods. *J. Obstet. Gynaecol. Res.* **35**, 525–532 (2009). 19527394 10.1111/j.1447-0756.2008.00971.x

[113] France, M. T. et al. VALENCIA: a nearest centroid classification method for vaginal microbial communities based on composition. *Microbiome* **8**, 166 (2020). 33228810 10.1186/s40168-020-00934-6 PMC7684964

[114] Marconi, C. et al. Characterization of the vaginal microbiome in women of reproductive age from 5 regions in Brazil. *Sex. Transm. Dis.* **47**, 562–569 (2020). 32520883 10.1097/OLQ.0000000000001204

[115] Borgdorff, H. et al. The association between ethnicity and vaginal microbiota composition in Amsterdam, the Netherlands. *PLoS ONE* **12**, e0181135 (2017). 28700747 10.1371/journal.pone.0181135 PMC5507447

[116] France, M., Alizadeh, M., Brown, S., Ma, B. & Ravel, J. Towards a deeper understanding of the vaginal microbiota. *Nat. Microbiol.* **7**, 367–378 (2022). 35246662 10.1038/s41564-022-01083-2 PMC8910585

[117] Muliyil, S. Linking the vaginal microbiome to women’s health. *Nat. Med*. 10.1038/d41591-023-00096-6 (2023). 10.1038/d41591-023-00096-6 37973863

[118] Lebeer, S. et al. A citizen-science-enabled catalogue of the vaginal microbiome and associated factors. *Nat. Microbiol.* **8**, 2183–2195 (2023). 37884815 10.1038/s41564-023-01500-0 PMC10627828

[119] Kumpitsch, C., Koskinen, K., Schöpf, V. & Moissl-Eichinger, C. The microbiome of the upper respiratory tract in health and disease. *BMC Biol.* **17**, 87 (2019). 31699101 10.1186/s12915-019-0703-z PMC6836414

[120] Natalini, J. G., Singh, S. & Segal, L. N. The dynamic lung microbiome in health and disease. *Nat. Rev. Microbiol.* **21**, 222–235 (2023). 36385637 10.1038/s41579-022-00821-x PMC9668228

[121] Pattaroni, C. et al. Early-life formation of the microbial and immunological environment of the human airways. *Cell Host Microbe* **24**, 857–865.e4 (2018). 30503510 10.1016/j.chom.2018.10.019

[122] Li, R., Li, J. & Zhou, X. Lung microbiome: new insights into the pathogenesis of respiratory diseases. *Signal Transduct. Target. Ther.* **9**, 19 (2024). 38228603 10.1038/s41392-023-01722-y PMC10791971

[123] Wypych, T. P., Wickramasinghe, L. C. & Marsland, B. J. The influence of the microbiome on respiratory health. *Nat. Immunol.* **20**, 1279–1290 (2019). 31501577 10.1038/s41590-019-0451-9

[124] Chen, L. et al. The long-term genetic stability and individual specificity of the human gut microbiome. *Cell* **184**, 2302–2315.e12 (2021). 33838112 10.1016/j.cell.2021.03.024

[125] Lozupone, C. A., Stombaugh, J. I., Gordon, J. I., Jansson, J. K. & Knight, R. Diversity, stability and resilience of the human gut microbiota. *Nature* **489**, 220–230 (2012). 22972295 10.1038/nature11550 PMC3577372

[126] Fassarella, M. et al. Gut microbiome stability and resilience: elucidating the response to perturbations in order to modulate gut health. *Gut* **70**, 595–605 (2021). 33051190 10.1136/gutjnl-2020-321747

[127] Sommer, F., Anderson, J. M., Bharti, R., Raes, J. & Rosenstiel, P. The resilience of the intestinal microbiota influences health and disease. *Nat. Rev. Microbiol.* **15**, 630–638 (2017). 28626231 10.1038/nrmicro.2017.58

[128] Zhou, X. et al. Longitudinal profiling of the microbiome at four body sites reveals core stability and individualized dynamics during health and disease. *Cell Host Microbe* **32**, 506–526.e9 (2024). 38479397 10.1016/j.chom.2024.02.012 PMC11022754

[129] Huang, S. et al. Human skin, oral, and gut microbiomes predict chronological age. *mSystems* **5**, e00630–19 (2020). 32047061 10.1128/mSystems.00630-19 PMC7018528

[130] Wang, L. et al. MIAOME: Human microbiome affect the host epigenome. *Comput. Struct. Biotechnol. J.* **20**, 2455–2463 (2022). 35664224 10.1016/j.csbj.2022.05.024 PMC9136154

[131] Kapil, V. et al. The noncanonical pathway for in vivo nitric oxide generation: the nitrate-nitrite-nitric oxide pathway. *Pharmacol. Rev.* **72**, 692–766 (2020). 32576603 10.1124/pr.120.019240

[132] Lundberg, J. O., Weitzberg, E. & Gladwin, M. T. The nitrate–nitrite–nitric oxide pathway in physiology and therapeutics. *Nat. Rev. Drug Discov.* **7**, 156–167 (2008). 18167491 10.1038/nrd2466

[133] Chai, X., Liu, L. & Chen, F. Oral nitrate-reducing bacteria as potential probiotics for blood pressure homeostasis. *Front. Cardiovasc. Med.* **11**, 1337281 (2024). 38638884 10.3389/fcvm.2024.1337281 PMC11024454

[134] Doel, J. J., Benjamin, N., Hector, M. P., Rogers, M. & Allaker, R. P. Evaluation of bacterial nitrate reduction in the human oral cavity. *Eur. J. Oral. Sci.* **113**, 14–19 (2005). 15693824 10.1111/j.1600-0722.2004.00184.x

[135] Goh, C. E. et al. Nitrite generating and depleting capacity of the oral microbiome and cardiometabolic risk: results from ORIGINS. *J. Am. Heart Assoc.* **11**, e023038 (2022). 35574962 10.1161/JAHA.121.023038 PMC9238569

[136] Blekkenhorst, L. C. et al. Nitrate, the oral microbiome, and cardiovascular health: a systematic literature review of human and animal studies. *Am. J. Clin. Nutr.* **107**, 504–522 (2018). 29635489 10.1093/ajcn/nqx046

[137] Kapil, V. et al. Physiological role for nitrate-reducing oral bacteria in blood pressure control. *Free Radic. Biol. Med.* **55**, 93–100 (2013). 23183324 10.1016/j.freeradbiomed.2012.11.013 PMC3605573

[138] Krautkramer, K. A., Fan, J. & Bäckhed, F. Gut microbial metabolites as multi-kingdom intermediates. *Nat. Rev. Microbiol.* **19**, 77–94 (2021). 32968241 10.1038/s41579-020-0438-4

[139] Sommer, F. & Bäckhed, F. The gut microbiota—masters of host development and physiology. *Nat. Rev. Microbiol.* **11**, 227–238 (2013). 23435359 10.1038/nrmicro2974

[140] Oliphant, K. & Allen-Vercoe, E. Macronutrient metabolism by the human gut microbiome: major fermentation by-products and their impact on host health. *Microbiome* **7**, 91 (2019). 31196177 10.1186/s40168-019-0704-8 PMC6567490

[141] Hill, J. H. & Round, J. L. SnapShot: Microbiota effects on host physiology. *Cell* **184**, 2796–2796.e1 (2021). 33989551 10.1016/j.cell.2021.04.026

[142] Koh, A., De Vadder, F., Kovatcheva-Datchary, P. & Bäckhed, F. From dietary fiber to host physiology: short-chain fatty acids as key bacterial metabolites. *Cell* **165**, 1332–1345 (2016). 27259147 10.1016/j.cell.2016.05.041

[143] Cummings, J. H., Pomare, E. W., Branch, W. J., Naylor, C. P. & Macfarlane, G. T. Short chain fatty acids in human large intestine, portal, hepatic and venous blood. *Gut* **28**, 1221–1227 (1987). 3678950 10.1136/gut.28.10.1221 PMC1433442

[144] Beynen, A. C., Buechler, K. F., Van Der Molen, A. J. & Geelen, M. J. H. The effects of lactate and acetate on fatty acid and cholesterol biosynthesis by isolated rat hepatocytes. *Int. J. Biochem.* **14**, 165–169 (1982). 6121723 10.1016/0020-711x(82)90135-5

[145] Yoshida, H., Ishii, M. & Akagawa, M. Propionate suppresses hepatic gluconeogenesis via GPR43/AMPK signaling pathway. *Arch. Biochem. Biophys.* **672**, 108057 (2019). 31356781 10.1016/j.abb.2019.07.022

[146] Wang, G. Y. et al. Propionate promotes gluconeogenesis by regulating mechanistic target of rapamycin (mTOR) pathway in calf hepatocytes. *Anim. Nutr.* **15**, 88–98 (2023). 37841648 10.1016/j.aninu.2023.07.001 PMC10568569

[147] Demigné, C. et al. Effect of propionate on fatty acid and cholesterol synthesis and on acetate metabolism in isolated rat hepatocytes. *Br. J. Nutr.* **74**, 209–219 (1995). 7547838 10.1079/bjn19950124

[148] Chambers, E. S. et al. Effects of targeted delivery of propionate to the human colon on appetite regulation, body weight maintenance and adiposity in overweight adults. *Gut* **64**, 1744–1754 (2015). 25500202 10.1136/gutjnl-2014-307913 PMC4680171

[149] Li, Z. et al. Butyrate reduces appetite and activates brown adipose tissue via the gut-brain neural circuit. *Gut* **67**, 1269–1279 (2018). 29101261 10.1136/gutjnl-2017-314050

[150] Trompette, A. et al. Gut-derived short-chain fatty acids modulate skin barrier integrity by promoting keratinocyte metabolism and differentiation. *Mucosal Immunol.* **15**, 908–926 (2022). 35672452 10.1038/s41385-022-00524-9 PMC9385498

[151] Jangi, S. et al. Microbial butyrate capacity is reduced in inflamed mucosa in patients with ulcerative colitis. *Sci. Rep.* **14**, 3479 (2024). 38347087 10.1038/s41598-024-54257-9 PMC10861456

[152] Kang, X. et al. *Roseburia intestinalis* generated butyrate boosts anti-PD-1 efficacy in colorectal cancer by activating cytotoxic CD8 ^+^ T cells. *Gut* **72**, 2112–2122 (2023). 37491158 10.1136/gutjnl-2023-330291 PMC10579466

[153] Zhang, Y., Tao, Y., Gu, Y. & Ma, Q. Butyrate facilitates immune clearance of colorectal cancer cells by suppressing STAT1-mediated PD-L1 expression. *Clinics* **78**, 100303 (2023). 37931529 10.1016/j.clinsp.2023.100303 PMC10654141

[154] Okumura, S. et al. Gut bacteria identified in colorectal cancer patients promote tumourigenesis via butyrate secretion. *Nat. Commun.* **12**, 5674 (2021). 34584098 10.1038/s41467-021-25965-x PMC8479117

[155] Wahlström, A., Sayin, S. I., Marschall, H.-U. & Bäckhed, F. Intestinal crosstalk between bile acids and microbiota and its impact on host metabolism. *Cell Metab.* **24**, 41–50 (2016). 27320064 10.1016/j.cmet.2016.05.005

[156] Collins, S. L., Stine, J. G., Bisanz, J. E., Okafor, C. D. & Patterson, A. D. Bile acids and the gut microbiota: metabolic interactions and impacts on disease. *Nat. Rev. Microbiol.* **21**, 236–247 (2023). 36253479 10.1038/s41579-022-00805-x PMC12536349

[157] Fogelson, K. A., Dorrestein, P. C., Zarrinpar, A. & Knight, R. The gut microbial bile acid modulation and its relevance to digestive health and diseases. *Gastroenterology* **164**, 1069–1085 (2023). 10.1053/j.gastro.2023.02.022 PMC10205675 36841488

[158] Xie, S. et al. Novel tripeptide RKH derived from *Akkermansia muciniphila* protects against lethal sepsis. *Gut* **73**, 78–91 (2023). 10.1136/gutjnl-2023-329996 37553229

[159] Jiang, J. et al. The gut metabolite indole-3-propionic acid activates ERK1 to restore social function and hippocampal inhibitory synaptic transmission in a 16p11.2 microdeletion mouse model. *Microbiome* **12**, 66 (2024). 38549163 10.1186/s40168-024-01755-7 PMC10976717

[160] Zhao, M. et al. Gut bacteria-driven homovanillic acid alleviates depression by modulating synaptic integrity. *Cell Metab.* **36**, 1000–1012.e6 (2024). 38582087 10.1016/j.cmet.2024.03.010

[161] Enamorado, M. et al. Immunity to the microbiota promotes sensory neuron regeneration. *Cell* **186**, 607–620.e17 (2023). 36640762 10.1016/j.cell.2022.12.037 PMC11512587

[162] Hosang, L. et al. The lung microbiome regulates brain autoimmunity. *Nature* **603**, 138–144 (2022). 35197636 10.1038/s41586-022-04427-4

[163] Oh, E. S. & Petronis, A. Origins of human disease: the chrono-epigenetic perspective. *Nat. Rev. Genet.* **22**, 533–546 (2021). 33903745 10.1038/s41576-021-00348-6

[164] Carter, B. & Zhao, K. The epigenetic basis of cellular heterogeneity. *Nat. Rev. Genet.* **22**, 235–250 (2021). 33244170 10.1038/s41576-020-00300-0 PMC10880028

[165] Fitz-James, M. H. & Cavalli, G. Molecular mechanisms of transgenerational epigenetic inheritance. *Nat. Rev. Genet.* **23**, 325–341 (2022). 34983971 10.1038/s41576-021-00438-5 PMC7619059

[166] Cavalli, G. & Heard, E. Advances in epigenetics link genetics to the environment and disease. *Nature* **571**, 489–499 (2019). 31341302 10.1038/s41586-019-1411-0

[167] Pepke, M. L., Hansen, S. B. & Limborg, M. T. Unraveling host regulation of gut microbiota through the epigenome–microbiome axis. *Trends Microbiol*. (2024) 10.1016/j.tim.2024.05.006 (2024). 10.1016/j.tim.2024.05.006 38839511

[168] Ansari, I. et al. The microbiota programs DNA methylation to control intestinal homeostasis and inflammation. *Nat. Microbiol.* **5**, 610–619 (2020). 32015497 10.1038/s41564-019-0659-3

[169] Mattei, A. L., Bailly, N. & Meissner, A. DNA methylation: a historical perspective. *Trends Genet* **38**, 676–707 (2022). 35504755 10.1016/j.tig.2022.03.010

[170] Crider, K. S., Yang, T. P., Berry, R. J. & Bailey, L. B. Folate and DNA methylation: a review of molecular mechanisms and the evidence for Folate’s role. *Adv. Nutr.* **3**, 21–38 (2012). 22332098 10.3945/an.111.000992 PMC3262611

[171] Bannister, A. J. & Kouzarides, T. Regulation of chromatin by histone modifications. *Cell Res.* **21**, 381–395 (2011). 21321607 10.1038/cr.2011.22 PMC3193420

[172] Millán-Zambrano, G., Burton, A., Bannister, A. J. & Schneider, R. Histone post-translational modifications—cause and consequence of genome function. *Nat. Rev. Genet.* **23**, 563–580 (2022). 35338361 10.1038/s41576-022-00468-7

[173] Furusawa, Y. et al. Commensal microbe-derived butyrate induces the differentiation of colonic regulatory T cells. *Nature* **504**, 446–450 (2013). 24226770 10.1038/nature12721

[174] Davison, J. M. et al. Microbiota regulate intestinal epithelial gene expression by suppressing the transcription factor Hepatocyte nuclear factor 4 alpha. *Genome Res.* **27**, 1195–1206 (2017). 28385711 10.1101/gr.220111.116 PMC5495071

[175] Mattick, J. S. et al. Long non-coding RNAs: definitions, functions, challenges and recommendations. *Nat. Rev. Mol. Cell Biol.* **24**, 430–447 (2023). 36596869 10.1038/s41580-022-00566-8 PMC10213152

[176] Nemeth, K., Bayraktar, R., Ferracin, M. & Calin, G. A. Non-coding RNAs in disease: from mechanisms to therapeutics. *Nat. Rev. Genet.* **25**, 211–232 (2024). 37968332 10.1038/s41576-023-00662-1

[177] Virtue, A. T. et al. The gut microbiota regulates white adipose tissue inflammation and obesity via a family of microRNAs. *Sci. Transl. Med.* **11**, eaav1892 (2019). 31189717 10.1126/scitranslmed.aav1892 PMC7050429

[178] Statello, L., Guo, C.-J., Chen, L.-L. & Huarte, M. Gene regulation by long non-coding RNAs and its biological functions. *Nat. Rev. Mol. Cell Biol.* **22**, 96–118 (2021). 33353982 10.1038/s41580-020-00315-9 PMC7754182

[179] Liang, L., Ai, L., Qian, J., Fang, J.-Y. & Xu, J. Long noncoding RNA expression profiles in gut tissues constitute molecular signatures that reflect the types of microbes. *Sci. Rep.* **5**, 11763 (2015). 26123364 10.1038/srep11763 PMC4485256

[180] Bach, J.-F. The effect of infections on susceptibility to autoimmune and allergic diseases. *N. Engl. J. Med.* **347**, 911–920 (2002). 12239261 10.1056/NEJMra020100

[181] Ege, M. J. et al. Exposure to environmental microorganisms and childhood asthma. *N. Engl. J. Med.* **364**, 701–709 (2011). 21345099 10.1056/NEJMoa1007302

[182] Kuehni, C. E., Strippoli, M. F., Low, N. & Silverman, M. Asthma in young South Asian women living in the United Kingdom: the importance of early life. *Clin. Exp. Allergy* **37**, 47–53 (2007). 17210041 10.1111/j.1365-2222.2006.02627.x

[183] Feltbower, R. G. et al. Trends in the incidence of childhood diabetes in South Asians and other children in Bradford, UK. *Diabet. Med.* **19**, 162–166 (2002). 11874434 10.1046/j.1464-5491.2002.00691.x

[184] Bodansky, H. J., Staines, A., Stephenson, C., Haigh, D. & Cartwright, R. Evidence for an environmental effect in the aetiology of insulin dependent diabetes in a transmigratory population. *BMJ* **304**, 1020–1022 (1992). 1586783 10.1136/bmj.304.6833.1020 PMC1881717

[185] Dean, G. & Elian, M. Age at immigration to England of Asian and Caribbean immigrants and the risk of developing multiple sclerosis. *J. Neurol. Neurosurg. Psychiatry* **63**, 565–568 (1997). 9408093 10.1136/jnnp.63.5.565 PMC2169801

[186] Gale, C. R. & Martyn, C. N. Migrant studies in multiple sclerosis. *Prog. Neurobiol.* **47**, 425–448 (1995). 8966212

[187] Strachan, D. P. Hay fever, hygiene, and household size. *BMJ* **299**, 1259–1260 (1989). 2513902 10.1136/bmj.299.6710.1259 PMC1838109

[188] Strachan, D. Family size, infection and atopy: the first decade of the ‘hygiene hypothesis’. *Thorax* **55**, 2S–10S (2000). 10.1136/thorax.55.suppl_1.s2 PMC1765943 10943631

[189] Finlay, B. B. et al. The hygiene hypothesis, the COVID pandemic, and consequences for the human microbiome. *Proc. Natl Acad. Sci.* **118**, e2010217118 (2021). 33472859 10.1073/pnas.2010217118 PMC8017729

[190] Parker, W. The ‘hygiene hypothesis’ for allergic disease is a misnomer. *BMJ* **349**, g5267–g5267 (2014). 10.1136/bmj.g5267 25161287

[191] Rook, G. A. W. et al. Mycobacteria and other environmental organisms as immunomodulators for immunoregulatory disorders. *Springe. Semin. Immunopathol.* **25**, 237–255 (2004). 10.1007/s00281-003-0148-9 15007629

[192] Rook, G. A. W. Microbes, immunoregulation, and the gut. *Gut* **54**, 317–320 (2005). 15710972 10.1136/gut.2004.053785 PMC1774411

[193] Donald, K. & Finlay, B. B. Early-life interactions between the microbiota and immune system: impact on immune system development and atopic disease. *Nat. Rev. Immunol*. **23**, 735–748 (2023). 10.1038/s41577-023-00874-w 37138015

[194] Van Der Waaij, D., Berghuis-de Vries, J. M. & Lekkerkerk-van Der Wees, J. E. C. Colonization resistance of the digestive tract in conventional and antibiotic-treated mice. *J. Hyg. (Lond.)* **69**, 405–411 (1971). 4999450 10.1017/s0022172400021653 PMC2130899

[195] Caballero-Flores, G., Pickard, J. M. & Núñez, G. Microbiota-mediated colonization resistance: mechanisms and regulation. *Nat. Rev. Microbiol*. **21**, 347–360. 10.1038/s41579-022-00833-7 (2022). 10.1038/s41579-022-00833-7 PMC10249723 36539611

[196] Bohnhoff, M. & Miller, C. P. Enhanced susceptibility to salmonella infection in streptomycin-treated mice*. *J. Infect. Dis.* **111**, 117–127 (1962). 13968487 10.1093/infdis/111.2.117

[197] Murray, C. J. L. et al. Global burden of bacterial antimicrobial resistance in 2019: a systematic analysis. *Lancet* **399**, 629–655 (2022). 35065702 10.1016/S0140-6736(21)02724-0 PMC8841637

[198] Blaser, M. J. Antibiotic use and its consequences for the normal microbiome. *Science* **352**, 544–545 (2016). 27126037 10.1126/science.aad9358 PMC4939477

[199] Lee, S. M. et al. Bacterial colonization factors control specificity and stability of the gut microbiota. *Nature* **501**, 426–429 (2013). 23955152 10.1038/nature12447 PMC3893107

[200] Tanzer, J. M., Kurasz, A. B. & Clive, J. Competitive displacement of mutans streptococci and inhibition of tooth decay by Streptococcus salivarius TOVE-R. *Infect. Immun.* **48**, 44–50 (1985). 3980093 10.1128/iai.48.1.44-50.1985 PMC261912

[201] Van Hoogmoed, C. G. et al. Reduction of periodontal pathogens adhesion by antagonistic strains. *Oral. Microbiol. Immunol.* **23**, 43–48 (2008). 18173797 10.1111/j.1399-302X.2007.00388.x

[202] Zipperer, A. et al. Human commensals producing a novel antibiotic impair pathogen colonization. *Nature* **535**, 511–516 (2016). 27466123 10.1038/nature18634

[203] Sugimoto, S. et al. Staphylococcus epidermidis esp degrades specific proteins associated with staphylococcus aureus biofilm formation and host-pathogen interaction. *J. Bacteriol.* **195**, 1645–1655 (2013). 23316041 10.1128/JB.01672-12 PMC3624567

[204] Torres Salazar, B. O. et al. Commensal production of a broad-spectrum and short-lived antimicrobial peptide polyene eliminates nasal Staphylococcus aureus. *Nat. Microbiol.* **9**, 200–213 (2023). 38110697 10.1038/s41564-023-01544-2 PMC11310079

[205] King, A. M. et al. Systematic mining of the human microbiome identifies antimicrobial peptides with diverse activity spectra. *Nat. Microbiol.* **8**, 2420–2434 (2023). 37973865 10.1038/s41564-023-01524-6

[206] Shin, R., Suzuki, M. & Morishita, Y. Influence of intestinal anaerobes and organic acids on the growth of enterohaemorrhagic Escherichia coli O157:H7. *J. Med. Microbiol.* **51**, 201–206 (2002). 11871614 10.1099/0022-1317-51-3-201

[207] Rolfe, R. D. Role of volatile fatty acids in colonization resistance to Clostridium difficile. *Infect. Immun.* **45**, 185–191 (1984). 6735467 10.1128/iai.45.1.185-191.1984 PMC263298

[208] Bohnhoff, M., Miller, C. P. & Martin, W. R. Resistance of the mouse’s intestinal tract to experimental Salmonella infection. *J. Exp. Med.* **120**, 805–816 (1964). 14247721 10.1084/jem.120.5.805 PMC2137858

[209] Buffie, C. G. et al. Precision microbiome reconstitution restores bile acid mediated resistance to Clostridium difficile. *Nature* **517**, 205–208 (2015). 25337874 10.1038/nature13828 PMC4354891

[210] Aoki, S. K. et al. Contact-dependent inhibition of growth in *Escherichia coli*. *Science* **309**, 1245–1248 (2005). 16109881 10.1126/science.1115109

[211] Aoki, S. K. et al. A widespread family of polymorphic contact-dependent toxin delivery systems in bacteria. *Nature* **468**, 439–442 (2010). 21085179 10.1038/nature09490 PMC3058911

[212] Flaugnatti, N. et al. Human commensal gut Proteobacteria withstand type VI secretion attacks through immunity protein-independent mechanisms. *Nat. Commun.* **12**, 5751 (2021). 34599171 10.1038/s41467-021-26041-0 PMC8486750

[213] Ross, B. D. et al. Human gut bacteria contain acquired interbacterial defence systems. *Nature* **575**, 224–228 (2019). 31666699 10.1038/s41586-019-1708-z PMC6938237

[214] Chatzidaki-Livanis, M., Geva-Zatorsky, N. & Comstock, L. E. *Bacteroides fragilis* type VI secretion systems use novel effector and immunity proteins to antagonize human gut Bacteroidales species. *Proc. Natl Acad. Sci.* **113**, 3627–3632 (2016). 26951680 10.1073/pnas.1522510113 PMC4822612

[215] Russell, A. B. et al. A type VI secretion-related pathway in bacteroidetes mediates interbacterial antagonism. *Cell Host Microbe* **16**, 227–236 (2014). 25070807 10.1016/j.chom.2014.07.007 PMC4136423

[216] Voravuthikunchai, S. P., Bilasoi, S. & Supamala, O. Antagonistic activity against pathogenic bacteria by human vaginal lactobacilli. *Anaerobe* **12**, 221–226 (2006). 16931064 10.1016/j.anaerobe.2006.06.003

[217] Turovskiy, Y., Sutyak Noll, K. & Chikindas, M. L. The aetiology of bacterial vaginosis: aetiology of bacterial vaginosis. *J. Appl. Microbiol.* **110**, 1105–1128 (2011). 21332897 10.1111/j.1365-2672.2011.04977.x PMC3072448

[218] Osbelt, L. et al. Klebsiella oxytoca inhibits Salmonella infection through multiple microbiota-context-dependent mechanisms. *Nat. Microbiol*. **9**, 1792–1811 (2024). 10.1038/s41564-024-01710-0 PMC11222139 38862602

[219] Stekel, D. First report of antimicrobial resistance pre-dates penicillin. *Nature* **562**, 192–192 (2018). 30305753 10.1038/d41586-018-06983-0

[220] Hutchings, M. I., Truman, A. W. & Wilkinson, B. Antibiotics: past, present and future. *Curr. Opin. Microbiol.* **51**, 72–80 (2019). 31733401 10.1016/j.mib.2019.10.008

[221] Varadan, S. R. et al. A just transition for antimicrobial resistance: planning for an equitable and sustainable future with antimicrobial resistance. *Lancet* **403**, 2766–2767 (2023). 10.1016/S0140-6736(23)01687-2 37696277

[222] Kimura, I. et al. Maternal gut microbiota in pregnancy influences offspring metabolic phenotype in mice. *Science* **367**, eaaw8429 (2020). 32108090 10.1126/science.aaw8429

[223] Vuong, H. E. et al. The maternal microbiome modulates fetal neurodevelopment in mice. *Nature* **586**, 281–286 (2020). 32968276 10.1038/s41586-020-2745-3 PMC7554197

[224] Sun, Z. et al. Revealing the importance of prenatal gut microbiome in offspring neurodevelopment in humans. *eBioMedicine* **90**, 104491 (2023). 36868051 10.1016/j.ebiom.2023.104491 PMC9996363

[225] Gomez de Agüero, M. et al. The maternal microbiota drives early postnatal innate immune development. *Science* **351**, 1296–1302 (2016). 26989247 10.1126/science.aad2571

[226] Bogaert, D. et al. Mother-to-infant microbiota transmission and infant microbiota development across multiple body sites. *Cell Host Microbe* **31**, 447–460.e6 (2023). 36893737 10.1016/j.chom.2023.01.018

[227] Selma-Royo, M. et al. Birthmode and environment-dependent microbiota transmission dynamics are complemented by breastfeeding during the first year. *Cell Host Microbe* **32**, 996–1010.e4 (2024). 38870906 10.1016/j.chom.2024.05.005 PMC11183301

[228] Paredes, A. et al. γ-Linolenic acid in maternal milk drives cardiac metabolic maturation. *Nature* **618**, 365–373 (2023). 37225978 10.1038/s41586-023-06068-7

[229] Caballero-Flores, G. et al. Maternal immunization confers protection to the offspring against an attaching and effacing pathogen through delivery of IgG in breast milk. *Cell Host Microbe* **25**, 313–323.e4 (2019). 30686564 10.1016/j.chom.2018.12.015 PMC6375740

[230] Atyeo, C. & Alter, G. The multifaceted roles of breast milk antibodies. *Cell* **184**, 1486–1499 (2021). 33740451 10.1016/j.cell.2021.02.031

[231] Zhong, Z. et al. Bifidobacterium animalis subsp. lactis Probio-M8 undergoes host adaptive evolution by glcU mutation and translocates to the infant’s gut via oral-/entero-mammary routes through lactation. *Microbiome* **10**, 197 (2022). 36419187 10.1186/s40168-022-01398-6 PMC9682673

[232] Barnett, D. J. M. et al. Human milk oligosaccharides, antimicrobial drugs, and the gut microbiota of term neonates: observations from the KOALA birth cohort study. *Gut Microbes* **15**, 2164152 (2023). 36617628 10.1080/19490976.2022.2164152 PMC9833409

[233] Cheung, K. Y. et al. Health and nutrition claims for infant formula: International Cross Sectional Survey. *BMJ* **380**, e071075 (2023). 10.1136/bmj-2022-071075 PMC9930154 36792145

[234] Baumann-Dudenhoeffer, A. M., D’Souza, A. W., Tarr, P. I., Warner, B. B. & Dantas, G. Infant diet and maternal gestational weight gain predict early metabolic maturation of gut microbiomes. *Nat. Med.* **24**, 1822–1829 (2018). 30374198 10.1038/s41591-018-0216-2 PMC6294307

[235] Mills, D. A., German, J. B., Lebrilla, C. B. & Underwood, M. A. Translating neonatal microbiome science into commercial innovation: metabolism of human milk oligosaccharides as a basis for probiotic efficacy in breast-fed infants. *Gut Microbes* **15**, 2192458 (2023). 37013357 10.1080/19490976.2023.2192458 PMC10075334

[236] Pérez-Escamilla, R. et al. Breastfeeding: crucially important, but increasingly challenged in a market-driven world. *Lancet* **401**, 472–485 (2023). 36764313 10.1016/S0140-6736(22)01932-8

[237] Baker, P. et al. The political economy of infant and young child feeding: confronting corporate power, overcoming structural barriers, and accelerating progress. *Lancet* **401**, 503–524 (2023). 36764315 10.1016/S0140-6736(22)01933-X

[238] Stinson, L. F. & Geddes, D. T. Microbial metabolites: the next frontier in human milk. *Trends Microbiol.* **30**, 408–410 (2022). 35282976 10.1016/j.tim.2022.02.007

[239] Dubois, L. et al. Paternal and induced gut microbiota seeding complement mother-to-infant transmission. *Cell Host Microbe* **32**, 1011–1024.e4 (2024). 38870892 10.1016/j.chom.2024.05.004

[240] Argaw-Denboba, A. et al. Paternal microbiome perturbations impact offspring fitness. *Nature* **629**, 652–659 (2024). 10.1038/s41586-024-07336-w PMC11096121 38693261

[241] Lawrence, R. J. David the ‘bubble boy’ and the boundaries of the human. *J. Am. Med. Assoc.* **253**, 74 (1985). 3964901

[242] Williams, S. C. P. Gnotobiotics. *Proc. Natl Acad. Sci.* **111**, 1661–1661 (2014). 24497491 10.1073/pnas.1324049111 PMC3918800

[243] Wostmann, B. S. The GERMFREE animal in nutritional studies. *Annu. Rev. Nutr.* **1**, 257–279 (1981). 6764717 10.1146/annurev.nu.01.070181.001353

[244] Basic, M. & Bleich, A. Gnotobiotics: past, present and future. *Lab. Anim.* **53**, 232–243 (2019). 31096878 10.1177/0023677219836715

[245] Fiebiger, U., Bereswill, S. & Heimesaat, M. M. Dissecting the interplay between intestinal microbiota and host immunity in health and disease: Lessons learned from germfree and gnotobiotic animal models. *Eur. J. Microbiol. Immunol.* **6**, 253–271 (2016). 10.1556/1886.2016.00036 PMC5146645 27980855

[246] Schwarzer, M. et al. *Lactobacillus plantarum* strain maintains growth of infant mice during chronic undernutrition. *Science* **351**, 854–857 (2016). 26912894 10.1126/science.aad8588

[247] Edwards, J. M. et al. Microbiota are critical for vascular physiology: Germ-free status weakens contractility and induces sex-specific vascular remodeling in mice. *Vasc. Pharmacol.* **125–126**, 106633 (2020). 10.1016/j.vph.2019.106633 PMC7036036 31843471

[248] Gordon, H. A., Wostmann, B. S. & Bruckner-Kardoss, E. Effects of microbial flora on cardiac output and other elements of blood circulation. *Exp. Biol. Med.* **114**, 301–304 (1963). 10.3181/00379727-114-28658 14101171

[249] Zhou, D. et al. Microbiota modulates cardiac transcriptional responses to intermittent hypoxia and hypercapnia. *Front. Physiol.* **12**, 680275 (2021). 34248668 10.3389/fphys.2021.680275 PMC8267877

[250] Crawford, P. A. et al. Regulation of myocardial ketone body metabolism by the gut microbiota during nutrient deprivation. *Proc. Natl Acad. Sci.* **106**, 11276–11281 (2009). 19549860 10.1073/pnas.0902366106 PMC2700149

[251] Jain, R., Waldvogel‐Thurlow, S., Darveau, R. & Douglas, R. Differences in the paranasal sinuses between germ‐free and pathogen‐free mice. *Int. Forum Allergy Rhinol.* **6**, 631–637 (2016). 27028583 10.1002/alr.21712

[252] Yun, Y. et al. Environmentally determined differences in the murine lung microbiota and their relation to alveolar architecture. *PLoS ONE* **9**, e113466 (2014). 25470730 10.1371/journal.pone.0113466 PMC4254600

[253] Woodward, B. A Study of the influence of the ambient microflora on the structure of lung alveolar macrophages and an ultrastructural comparison of lung and peritoneal macrophages in germ‐free and conventionally reared mice. *J. Morphol.* **169**, 283–291 (1981). 7053129 10.1002/jmor.1051690304

[254] Dolma, K. et al. Effects of hyperoxia on alveolar and pulmonary vascular development in germ-free mice. *Am. J. Physiol. -Lung Cell. Mol. Physiol.* **318**, L421–L428 (2020). 31644312 10.1152/ajplung.00316.2019 PMC7052667

[255] Ashley, S. L. et al. Lung and gut microbiota are altered by hyperoxia and contribute to oxygen-induced lung injury in mice. *Sci. Transl. Med*. **12**, eaau9959 (2020). 10.1126/scitranslmed.aau9959 PMC7732030 32801143

[256] Wostmann, B. S. *Germfree and Gnotobiotic Animal Models: Background and Applications*. (CRC Press, 2020). 10.1201/9780138753320.

[257] Al-Asmakh, M. & Zadjali, F. Use of germ-free animal models in microbiota-related research. *J. Microbiol. Biotechnol.* **25**, 1583–1588 (2015). 26032361 10.4014/jmb.1501.01039

[258] Geissinger, H. D. & Abandowitz, H. M. Scanning electron and light microscopy of the cecum of germ-free and conventional mice. *Trans. Am. Microsc. Soc.* **96**, 254 (1977). 878146

[259] Khoury, K. A., Floch, M. H. & Hersh, T. Small intestinal mucosal cell proliferation and bacterial flora in the conventionalization of the germfree mouse. *J. Exp. Med.* **130**, 659–670 (1969). 4896909 10.1084/jem.130.3.659 PMC2138714

[260] Bolsega, S. et al. The genetic background is shaping cecal enlargement in the absence of intestinal microbiota. *Nutrients* **15**, 636 (2023). 36771343 10.3390/nu15030636 PMC9921660

[261] Loesche, W. J. Accumulation of endogenous carbohydrate-containing compounds in the cecum of the germfree rat. *Exp. Biol. Med.* **131**, 387–392 (1969). 10.3181/00379727-131-33885 5787116

[262] McVey Neufeld, K. A., Perez‐Burgos, A., Mao, Y. K., Bienenstock, J. & Kunze, W. A. The gut microbiome restores intrinsic and extrinsic nerve function in germ‐free mice accompanied by changes in calbindin. *Neurogastroenterol. Motil.* **27**, 627–636 (2015). 25727007 10.1111/nmo.12534

[263] McVey Neufeld, K. A., Mao, Y. K., Bienenstock, J., Foster, J. A. & Kunze, W. A. The microbiome is essential for normal gut intrinsic primary afferent neuron excitability in the mouse. *Neurogastroenterol. Motil.* **25**, 183 (2013). 23181420 10.1111/nmo.12049

[264] Touw, K. et al. Mutual reinforcement of pathophysiological host‐microbe interactions in intestinal stasis models. *Physiol. Rep.* **5**, e13182 (2017). 28320888 10.14814/phy2.13182 PMC5371559

[265] Niimi, K. & Takahashi, E. New system to examine the activity and water and food intake of germ-free mice in a sealed positive-pressure cage. *Heliyon* **5**, e02176 (2019). 31463382 10.1016/j.heliyon.2019.e02176 PMC6706585

[266] Jervis, H. R. & Biggers, D. C. Mucosal enzymes in the cecum of conventional and germfree mice. *Anat. Rec.* **148**, 591–597 (1964). 14138162 10.1002/ar.1091480410

[267] Sumi, Y., Miyakawa, M., Kanzaki, M. & Kotake, Y. Vitamin B-6 deficiency in germfree rats. *J. Nutr.* **107**, 1707–1714 (1977). 894368 10.1093/jn/107.9.1707

[268] Ikeda, M. et al. The differences of the metabolism related to vitamin B6-dependent enzymes among vitamin B6-deficient germ-free and conventional rats. *J. Nutr. Sci. Vitaminol. (Tokyo)* **25**, 131–139 (1979). 501446 10.3177/jnsv.25.131

[269] Hirayama, K., Uetsuka, K., Kuwabara, Y., Tamura, M. & Itoh, K. Vitamin K deficiency of germfree mice caused by feeding standard purified diet sterilized by.GAMMA.-irradiation. *Exp. Anim.* **56**, 273–278 (2007). 17660681 10.1538/expanim.56.273

[270] Mishima, E. et al. Germ-free conditions modulate host purine metabolism, exacerbating adenine-induced kidney damage. *Toxins* **12**, 547 (2020). 32859011 10.3390/toxins12090547 PMC7551802

[271] Koopman, J. P. in *Encyclopedia of Immunology* 990–992 (Elsevier, 1998). 10.1006/rwei.1999.0256.

[272] Hoces, D. et al. Metabolic reconstitution of germ-free mice by a gnotobiotic microbiota varies over the circadian cycle. *PLoS Biol.* **20**, e3001743 (2022). 36126044 10.1371/journal.pbio.3001743 PMC9488797

[273] Weger, B. D. et al. The mouse microbiome is required for sex-specific diurnal rhythms of gene expression and metabolism. *Cell Metab.* **29**, 362–382.e8 (2019). 30344015 10.1016/j.cmet.2018.09.023 PMC6370974

[274] Sharma, S. P. & Suk, K. T. Microbial influence on liver regeneration: understanding gut microbiota and hepatic recovery post partial hepatectomy. *Hepatobiliary Surg. Nutr.* **13**, 314–316 (2024). 38617487 10.21037/hbsn-23-663 PMC11007337

[275] Xu, Z., Jiang, N., Xiao, Y., Yuan, K. & Wang, Z. The role of gut microbiota in liver regeneration. *Front. Immunol.* **13**, 1003376 (2022). 36389782 10.3389/fimmu.2022.1003376 PMC9647006

[276] Kennedy, E. A., King, K. Y. & Baldridge, M. T. Mouse microbiota models: comparing germ-free mice and antibiotics treatment as tools for modifying gut bacteria. *Front. Physiol.* **9**, 1534 (2018). 30429801 10.3389/fphys.2018.01534 PMC6220354

[277] Wostmann, B. S., Pleasants, J. R., Bealmear, P. & Kincade, P. W. Serum proteins and lymphoid tissues in germ-free mice fed a chemically defined, water soluble, low molecular weight diet. *Immunology* **19**, 443–448 (1970). 5471828 PMC1455676

[278] Jung, C., Hugot, J.-P. & Barreau, F. Peyer’s patches: the immune sensors of the intestine. *Int. J. Inflamm.* **2010**, 1–12 (2010). 10.4061/2010/823710 PMC3004000 21188221

[279] Konjar, Š., Ferreira, C., Blankenhaus, B. & Veldhoen, M. Intestinal barrier interactions with specialized CD8 T cells. *Front. Immunol.* **8**, 1281 (2017). 29075263 10.3389/fimmu.2017.01281 PMC5641586

[280] Ivanov, I. I. et al. Induction of Intestinal Th17. *Cells Segmented Filamentous Bact. Cell* **139**, 485–498 (2009). 10.1016/j.cell.2009.09.033 PMC2796826 19836068

[281] Schoenborn, A. A. et al. The enteric microbiota regulates jejunal Paneth cell number and function without impacting intestinal stem cells. *Gut Microbes* **10**, 45–58 (2019). 29883265 10.1080/19490976.2018.1474321 PMC6363071

[282] Macpherson, A. J., McCoy, K. D., Johansen, F.-E. & Brandtzaeg, P. The immune geography of IgA induction and function. *Mucosal Immunol.* **1**, 11–22 (2008). 19079156 10.1038/mi.2007.6

[283] Round, J. L. & Mazmanian, S. K. The gut microbiota shapes intestinal immune responses during health and disease. *Nat. Rev. Immunol.* **9**, 313–323 (2009). 19343057 10.1038/nri2515 PMC4095778

[284] Zaph, C. et al. Commensal-dependent expression of IL-25 regulates the IL-23–IL-17 axis in the intestine. *J. Exp. Med.* **205**, 2191–2198 (2008). 18762568 10.1084/jem.20080720 PMC2556798

[285] Sprinz, H. et al. The response of the germfree guinea pig to oral bacterial challenge with Escherichia coli and Shigella flexneri. *Am. J. Pathol.* **39**, 681–695 (1961). 13915950 PMC1942415

[286] Wang, C. et al. Sjögren-like lacrimal keratoconjunctivitis in germ-free mice. *Int. J. Mol. Sci.* **19**, 565 (2018). 29438346 10.3390/ijms19020565 PMC5855787

[287] Braniste, V. et al. The gut microbiota influences blood-brain barrier permeability in mice. *Sci. Transl. Med*. **6** (2014). 10.1126/scitranslmed.3009759 PMC4396848 25411471

[288] Lu, J. et al. Microbiota influence the development of the brain and behaviors in C57BL/6J mice. *PLoS ONE* **13**, e0201829 (2018). 30075011 10.1371/journal.pone.0201829 PMC6075787

[289] Luczynski, P. et al. Adult microbiota‐deficient mice have distinct dendritic morphological changes: differential effects in the amygdala and hippocampus. *Eur. J. Neurosci.* **44**, 2654–2666 (2016). 27256072 10.1111/ejn.13291 PMC5113767

[290] Luczynski, P. et al. Growing up in a bubble: using germ-free animals to assess the influence of the gut microbiota on brain and behavior. *Int. J. Neuropsychopharmacol.* **19**, pyw020 (2016). 26912607 10.1093/ijnp/pyw020 PMC5006193

[291] Wu, W.-L. et al. Microbiota regulate social behaviour via stress response neurons in the brain. *Nature* **595**, 409–414 (2021). 34194038 10.1038/s41586-021-03669-y PMC8346519

[292] Cescon, M. et al. Gut microbiota depletion delays somatic peripheral nerve development and impairs neuromuscular junction maturation. *Gut Microbes* **16**, 2363015 (2024). 38845453 10.1080/19490976.2024.2363015 PMC11164225

[293] Mai, H. et al. Whole-body cellular mapping in mouse using standard IgG antibodies. *Nat. Biotechnol*. **42**, 617–627. 10.1038/s41587-023-01846-0 (2023). 10.1038/s41587-023-01846-0 PMC11021200 37430076

[294] De Vadder, F. et al. Gut microbiota regulates maturation of the adult enteric nervous system via enteric serotonin networks. *Proc. Natl Acad. Sci.* **115**, 6458–6463 (2018). 29866843 10.1073/pnas.1720017115 PMC6016808

[295] Kabouridis, P. S. et al. Microbiota controls the homeostasis of glial cells in the gut lamina propria. *Neuron* **85**, 289–295 (2015). 25578362 10.1016/j.neuron.2014.12.037 PMC4306542

[296] Shimizu, K. et al. Normalization of reproductive function in germfree mice following bacterial contamination. *Exp. Anim.* **47**, 151–158 (1998). 9816490 10.1538/expanim.47.151

[297] Al-Asmakh, M. et al. The gut microbiota and developmental programming of the testis in mice. *PLoS ONE* **9**, e103809 (2014). 25118984 10.1371/journal.pone.0103809 PMC4132106

[298] Vahidi, G. et al. Germ‐free C57BL /6 mice have increased bone mass and altered matrix properties but not decreased bone fracture resistance. *J. Bone Miner. Res.* **38**, 1154–1174 (2023). 37221143 10.1002/jbmr.4835 PMC10530360

[299] Ohlsson, C. et al. Regulation of bone mass by the gut microbiota is dependent on NOD1 and NOD2 signaling. *Cell. Immunol.* **317**, 55–58 (2017). 28576260 10.1016/j.cellimm.2017.05.003

[300] Czernik, P. J. et al. Reconstitution of the host holobiont in germ-free born male rats acutely increases bone growth and affects marrow cellular content. *Physiol. Genomics* **53**, 518–533 (2021). 34714176 10.1152/physiolgenomics.00017.2021 PMC8714805

[301] Lahiri, S. et al. The gut microbiota influences skeletal muscle mass and function in mice. *Sci. Transl. Med.* **11**, eaan5662 (2019). 31341063 10.1126/scitranslmed.aan5662 PMC7501733

[302] Qiu, Y. et al. Depletion of gut microbiota induces skeletal muscle atrophy by FXR-FGF15/19 signalling. *Ann. Med.* **53**, 508–522 (2021). 33783283 10.1080/07853890.2021.1900593 PMC8018554

[303] Yan, H. et al. Gut microbiota can transfer fiber characteristics and lipid metabolic profiles of skeletal muscle from pigs to germ-free mice. *Sci. Rep.* **6**, 31786 (2016). 27545196 10.1038/srep31786 PMC4992887

[304] Bäckhed, F. et al. The gut microbiota as an environmental factor that regulates fat storage. *Proc. Natl Acad. Sci.* **101**, 15718–15723 (2004). 15505215 10.1073/pnas.0407076101 PMC524219

[305] Mestdagh, R. et al. Gut microbiota modulate the metabolism of brown adipose tissue in mice. *J. Proteome Res.* **11**, 620–630 (2012). 22053906 10.1021/pr200938v

[306] Suárez-Zamorano, N. et al. Microbiota depletion promotes browning of white adipose tissue and reduces obesity. *Nat. Med.* **21**, 1497–1501 (2015). 26569380 10.1038/nm.3994 PMC4675088

[307] Bäckhed, F., Manchester, J. K., Semenkovich, C. F. & Gordon, J. I. Mechanisms underlying the resistance to diet-induced obesity in germ-free mice. *Proc. Natl Acad. Sci.* **104**, 979–984 (2007). 17210919 10.1073/pnas.0605374104 PMC1764762

[308] Kim, H. J. et al. Microbiota influences host exercise capacity via modulation of skeletal muscle glucose metabolism in mice. *Exp. Mol. Med.* **55**, 1820–1830 (2023). 37542180 10.1038/s12276-023-01063-4 PMC10474268

[309] Uberoi, A. et al. Commensal microbiota regulates skin barrier function and repair via signaling through the aryl hydrocarbon receptor. *Cell Host Microbe* **29**, 1235–1248.e8 (2021). 34214492 10.1016/j.chom.2021.05.011 PMC8364505

[310] Wang, G. et al. Bacteria induce skin regeneration via IL-1β signaling. *Cell Host Microbe* **29**, 777–791.e6 (2021). 33798492 10.1016/j.chom.2021.03.003 PMC8122070

[311] Tazume, S. et al. Effects of germfree status and food restriction on longevity and growth of mice. *Exp. Anim.* **40**, 517–522 (1991). 10.1538/expanim1978.40.4_517 1748169

[312] Gordon, H. A., Bruckner-kardoss, E. & Wostmann, B. S. Aging in germ-free mice: life tables and lesions observed at natural death. *J. Gerontol.* **21**, 380–387 (1966). 5944800 10.1093/geronj/21.3.380

[313] Gilbert, J. A. & Neufeld, J. D. Life in a world without microbes. *PLoS Biol.* **12**, e1002020 (2014). 25513890 10.1371/journal.pbio.1002020 PMC4267716

[314] Olm, M. R. et al. Robust variation in infant gut microbiome assembly across a spectrum of lifestyles. *Science* **376**, 1220–1223 (2022). 35679413 10.1126/science.abj2972 PMC9894631

[315] Blaser, M. J. & Falkow, S. What are the consequences of the disappearing human microbiota? *Nat. Rev. Microbiol.* **7**, 887–894 (2009). 19898491 10.1038/nrmicro2245 PMC9354563

[316] Clemente, J. C. et al. The microbiome of uncontacted Amerindians. *Sci. Adv.* **1**, e1500183 (2015). 26229982 10.1126/sciadv.1500183 PMC4517851

[317] Sonnenburg, E. D. et al. Diet-induced extinctions in the gut microbiota compound over generations. *Nature* **529**, 212–215 (2016). 26762459 10.1038/nature16504 PMC4850918

[318] Carter, M. M. et al. Ultra-deep sequencing of Hadza hunter-gatherers recovers vanishing gut microbes. *Cell* **186**, 3111–3124.e13 (2023). 10.1016/j.cell.2023.05.046 PMC10330870 37348505

[319] Wu, J., Wang, Q., Wang, D., Wong, A. C. N. & Wang, G.-H. Axenic and gnotobiotic insect technologies in research on host–microbiota interactions. *Trends Microbiol*. **31**, 858–871 (2023). 10.1016/j.tim.2023.02.007 36906503

[320] Sagan, L. On the origin of mitosing cells. *J. Theor. Biol.* **14**, 225–IN6 (1967). 10.1016/0022-5193(67)90079-3 11541392

[321] Moreira, D., Le Guyader, H. & Philippe, H. The origin of red algae and the evolution of chloroplasts. *Nature* **405**, 69–72 (2000). 10811219 10.1038/35011054

[322] Fan, L. et al. Phylogenetic analyses with systematic taxon sampling show that mitochondria branch within Alphaproteobacteria. *Nat. Ecol. Evol.* **4**, 1213–1219 (2020). 32661403 10.1038/s41559-020-1239-x

[323] Gogoi, J. et al. Switching a conflicted bacterial DTD-tRNA code is essential for the emergence of mitochondria. *Sci. Adv.* **8**, eabj7307 (2022). 35020439 10.1126/sciadv.abj7307 PMC8754408

[324] Lederberg, J. Infectious History. *Science* **288**, 287–293 (2000). 10777411 10.1126/science.288.5464.287

[325] Coale, T. H. et al. Nitrogen-fixing organelle in a marine alga. *Science* **384**, 217–222 (2024). 38603509 10.1126/science.adk1075

[326] Boyd, B. M. et al. Stochasticity, determinism, and contingency shape genome evolution of endosymbiotic bacteria. *Nat. Commun.* **15**, 4571 (2024). 38811551 10.1038/s41467-024-48784-2 PMC11137140

[327] Baedke, J., Fábregas‐Tejeda, A. & Nieves Delgado, A. The holobiont concept before Margulis. *J. Exp. Zool. B: Mol. Dev. Evol.* **334**, 149–155 (2020). 32039567 10.1002/jez.b.22931

[328] Margulis, L. & Fester, R. Bellagio conference and book. Symbiosis as Source of Evolutionary Innovation: Speciation and Morphogenesis. Conference-June 25-30, 1989, Bellagio Conference Center, Italy. *Symbiosis Phila. Pa* **11**, 93–101 (1991). 11538111

[329] Turnbaugh, P. J. et al. The Human Microbiome Project. *Nature* **449**, 804–810 (2007). 17943116 10.1038/nature06244 PMC3709439

[330] King, I. L. & Divangahi, M. Training the metaorganism: the microbial counterpart. *Cell* **184**, 574–576 (2021). 33545033 10.1016/j.cell.2021.01.009

[331] Gill, S. R. et al. Metagenomic analysis of the human distal gut microbiome. *Science* **312**, 1355–1359 (2006). 16741115 10.1126/science.1124234 PMC3027896

[332] Macpherson, A. J. Do the microbiota influence vaccines and protective immunity to pathogens?: issues of sovereignty, federalism, and points-testing in the prokaryotic and eukaryotic spaces of the host-microbial superorganism. *Cold Spring Harb. Perspect. Biol.* **10**, a029363 (2018). 28432128 10.1101/cshperspect.a029363 PMC5793759

[333] Thaiss, C. A., Zeevi, D., Levy, M., Segal, E. & Elinav, E. A day in the life of the meta-organism: diurnal rhythms of the intestinal microbiome and its host. *Gut Microbes* **6**, 137–142 (2015). 25901892 10.1080/19490976.2015.1016690 PMC4615721

[334] Muegge, B. D. et al. Diet drives convergence in gut microbiome functions across mammalian phylogeny and within humans. *Science* **332**, 970–974 (2011). 21596990 10.1126/science.1198719 PMC3303602

[335] Segre, J. A. & Salafsky, N. Hominid superorganisms. *Science* **353**, 350–351 (2016). 27463659 10.1126/science.aag2788

[336] Wenseleers, T. The superorganism revisited. *BioScience* **59**, 702–705 (2009).

[337] Jefferson, R. Agriculture and the Third World. 1089446492 Bytes. 10.6084/M9.FIGSHARE.7945781 (2019).

[338] Rosenberg, E., Koren, O., Reshef, L., Efrony, R. & Zilber-Rosenberg, I. The role of microorganisms in coral health, disease and evolution. *Nat. Rev. Microbiol.* **5**, 355–362 (2007). 17384666 10.1038/nrmicro1635

[339] Zilber-Rosenberg, I. & Rosenberg, E. Role of microorganisms in the evolution of animals and plants: the hologenome theory of evolution. *FEMS Microbiol. Rev.* **32**, 723–735 (2008). 18549407 10.1111/j.1574-6976.2008.00123.x

[340] Rosenberg, E. & Zilber-Rosenberg, I. The hologenome concept of evolution after 10 years. *Microbiome* **6**, 78 (2018). 29695294 10.1186/s40168-018-0457-9 PMC5922317

[341] Alberdi, A., Andersen, S. B., Limborg, M. T., Dunn, R. R. & Gilbert, M. T. P. Disentangling host–microbiota complexity through hologenomics. *Nat. Rev. Genet.* **23**, 281–297 (2022). 34675394 10.1038/s41576-021-00421-0

[342] Liu, H. et al. Symbiodinium genomes reveal adaptive evolution of functions related to coral-dinoflagellate symbiosis. *Commun. Biol.* **1**, 95 (2018). 30271976 10.1038/s42003-018-0098-3 PMC6123633

[343] Rosado, P. M. et al. Marine probiotics: increasing coral resistance to bleaching through microbiome manipulation. *ISME J.* **13**, 921–936 (2019). 30518818 10.1038/s41396-018-0323-6 PMC6461899

[344] Dove, S. G., Brown, K. T., Van Den Heuvel, A., Chai, A. & Hoegh-Guldberg, O. Ocean warming and acidification uncouple calcification from calcifier biomass which accelerates coral reef decline. *Commun. Earth Environ.* **1**, 55 (2020).

[345] Vanwonterghem, I. & Webster, N. S. Coral reef microorganisms in a changing climate. *iScience* **23**, 100972 (2020). 32208346 10.1016/j.isci.2020.100972 PMC7096749

[346] Biget, M. et al. Evaluating the hologenome concept by analyzing the root-endosphere microbiota of chimeric plants. *iScience* **26**, 106031 (2023). 36824281 10.1016/j.isci.2023.106031 PMC9941212

[347] Suárez, J. & Triviño, V. What Is a hologenomic adaptation? emergent individuality and inter-identity in multispecies systems. *Front. Psychol.* **11**, 187 (2020). 32194470 10.3389/fpsyg.2020.00187 PMC7064717

[348] Zepeda Mendoza, M. L. et al. Hologenomic adaptations underlying the evolution of sanguivory in the common vampire bat. *Nat. Ecol. Evol.* **2**, 659–668 (2018). 29459707 10.1038/s41559-018-0476-8 PMC5868727

[349] Shigenobu, S., Watanabe, H., Hattori, M., Sakaki, Y. & Ishikawa, H. Genome sequence of the endocellular bacterial symbiont of aphids Buchnera sp. APS. *Nature* **407**, 81–86 (2000). 10993077 10.1038/35024074

[350] Hansen, A. K. & Moran, N. A. Aphid genome expression reveals host–symbiont cooperation in the production of amino acids. *Proc. Natl Acad. Sci.* **108**, 2849–2854 (2011). 21282658 10.1073/pnas.1013465108 PMC3041126

[351] Bennett, G. M. & Moran, N. A. Heritable symbiosis: the advantages and perils of an evolutionary rabbit hole. *Proc. Natl Acad. Sci.* **112**, 10169–10176 (2015). 25713367 10.1073/pnas.1421388112 PMC4547261

[352] Moran, N. A. Symbiosis as an adaptive process and source of phenotypic complexity. *Proc. Natl Acad. Sci.* **104**, 8627–8633 (2007). 17494762 10.1073/pnas.0611659104 PMC1876439

[353] Lebreton, F. et al. Tracing the enterococci from paleozoic origins to the hospital. *Cell* **169**, 849–861.e13 (2017). 28502769 10.1016/j.cell.2017.04.027 PMC5499534

[354] Kutschera, U. Darwin–Wallace principle of natural selection. *Nature* **453**, 27–27 (2008). 18451834 10.1038/453027b

[355] Wallace, R. Extending the modern synthesis: the evolution of ecosystems. *Nat. Preced*. 10.1038/npre.2010.4496.2 (2010).

[356] Gardner, A. The genetical theory of multilevel selection. *J. Evol. Biol.* **28**, 305–319 (2015). 25475922 10.1111/jeb.12566 PMC4415573

[357] Suzuki, T. A. & Ley, R. E. The role of the microbiota in human genetic adaptation. *Science* **370**, eaaz6827 (2020). 33273073 10.1126/science.aaz6827

[358] Henry, L. P., Bruijning, M., Forsberg, S. K. G. & Ayroles, J. F. The microbiome extends host evolutionary potential. *Nat. Commun.* **12**, 5141 (2021). 34446709 10.1038/s41467-021-25315-x PMC8390463

[359] Ferreiro, A., Crook, N., Gasparrini, A. J. & Dantas, G. Multiscale evolutionary dynamics of host-associated microbiomes. *Cell* **172**, 1216–1227 (2018). 29522743 10.1016/j.cell.2018.02.015 PMC5846202

[360] Grice, E. A. & Segre, J. A. The human microbiome: our second genome. *Annu. Rev. Genomics Hum. Genet.* **13**, 151–170 (2012). 22703178 10.1146/annurev-genom-090711-163814 PMC3518434

[361] Zhang, X. et al. Brain control of humoral immune responses amenable to behavioural modulation. *Nature* **581**, 204–208 (2020). 32405000 10.1038/s41586-020-2235-7

[362] Akkaya, M., Kwak, K. & Pierce, S. K. B cell memory: building two walls of protection against pathogens. *Nat. Rev. Immunol.* **20**, 229–238 (2020). 31836872 10.1038/s41577-019-0244-2 PMC7223087

[363] Jin, H., Li, M., Jeong, E., Castro-Martinez, F. & Zuker, C. S. A body–brain circuit that regulates body inflammatory responses. *Nature* **630**, 695–703 (2024). 10.1038/s41586-024-07469-y PMC11186780 38692285

[364] Nagashima, K. et al. Mapping the T cell repertoire to a complex gut bacterial community. *Nature* **621**, 162–170 (2023). 10.1038/s41586-023-06431-8 PMC10948025 37587342

[365] Omenetti, S. et al. The intestine harbors functionally distinct homeostatic tissue-resident and inflammatory Th17 cells. *Immunity* **51**, 77–89.e6 (2019). 31229354 10.1016/j.immuni.2019.05.004 PMC6642154

[366] Moor, K. et al. High-avidity IgA protects the intestine by enchaining growing bacteria. *Nature* **544**, 498–502 (2017). 28405025 10.1038/nature22058

[367] Rollenske, T. et al. Parallelism of intestinal secretory IgA shapes functional microbial fitness. *Nature* **598**, 657–661 (2021). 34646015 10.1038/s41586-021-03973-7

[368] Huus, K. E., Petersen, C. & Finlay, B. B. Diversity and dynamism of IgA−microbiota interactions. *Nat. Rev. Immunol.* **21**, 514–525 (2021). 33568782 10.1038/s41577-021-00506-1

[369] Moreno-Sabater, A. et al. Intestinal Candida albicans overgrowth in IgA deficiency. *J. Allergy Clin. Immunol*. **152**, 748–759.e3. (2023). 10.1016/j.jaci.2023.03.033 37169153

[370] Conrey, P. E. et al. IgA deficiency destabilizes homeostasis toward intestinal microbes and increases systemic immune dysregulation. *Sci. Immunol.* **8**, eade2335 (2023). 37235682 10.1126/sciimmunol.ade2335 PMC11623094

[371] Zhang, C. et al. An overview of host‐derived molecules that interact with gut microbiota. *iMeta* **2**, e88 (2023). 38868433 10.1002/imt2.88 PMC10989792

[372] Herr, A. B., White, C. L., Milburn, C., Wu, C. & Bjorkman, P. J. Bivalent binding of IgA1 to FcαRI suggests a mechanism for cytokine activation of IgA phagocytosis. *J. Mol. Biol.* **327**, 645–657 (2003). 12634059 10.1016/s0022-2836(03)00149-9

[373] Donaldson, G. P. et al. Gut microbiota utilize immunoglobulin A for mucosal colonization. *Science* **360**, 795–800 (2018). 29724905 10.1126/science.aaq0926 PMC5973787

[374] Li, Y., Wang, J., Wang, R., Chang, Y. & Wang, X. Gut bacteria induce IgA expression in pituitary hormone-secreting cells during aging. *iScience* **26**, 107747 (2023). 37692284 10.1016/j.isci.2023.107747 PMC10492204

[375] Lazzaro, B. P., Zasloff, M. & Rolff, J. Antimicrobial peptides: application informed by evolution. *Science* **368**, eaau5480 (2020). 32355003 10.1126/science.aau5480 PMC8097767

[376] Mookherjee, N., Anderson, M. A., Haagsman, H. P. & Davidson, D. J. Antimicrobial host defence peptides: functions and clinical potential. *Nat. Rev. Drug Discov.* **19**, 311–332 (2020). 32107480 10.1038/s41573-019-0058-8

[377] Jarret, A. et al. Enteric nervous system-derived IL-18 orchestrates mucosal barrier immunity. *Cell* **180**, 50–63.e12 (2020). 31923399 10.1016/j.cell.2019.12.016 PMC7339937

[378] Pierre, J. F. et al. Peptide YY: a Paneth cell antimicrobial peptide that maintains *Candida* gut commensalism. *Science* **381**, 502–508 (2023). 37535745 10.1126/science.abq3178 PMC10876062

[379] Unckless, R. L., Howick, V. M. & Lazzaro, B. P. Convergent balancing selection on an antimicrobial peptide in Drosophila. *Curr. Biol.* **26**, 257–262 (2016). 26776733 10.1016/j.cub.2015.11.063 PMC4729654

[380] Unckless, R. L. & Lazzaro, B. P. The potential for adaptive maintenance of diversity in insect antimicrobial peptides. *Philos. Trans. R. Soc. B: Biol. Sci.* **371**, 20150291 (2016). 10.1098/rstb.2015.0291 PMC4874389 27160594

[381] Zanchi, C., Johnston, P. R. & Rolff, J. Evolution of defence cocktails: antimicrobial peptide combinations reduce mortality and persistent infection. *Mol. Ecol.* **26**, 5334–5343 (2017). 28762573 10.1111/mec.14267

[382] Hanson, M. A., Grollmus, L. & Lemaitre, B. Ecology-relevant bacteria drive the evolution of host antimicrobial peptides in. *Drosoph. Sci.* **381**, eadg5725 (2023). 10.1126/science.adg5725 37471548

[383] Nakatsuji, T. et al. Antimicrobials from human skin commensal bacteria protect against *Staphylococcus aureus* and are deficient in atopic dermatitis. *Sci. Transl. Med.* **9**, eaah4680 (2017). 28228596 10.1126/scitranslmed.aah4680 PMC5600545

[384] Mukherjee, S. & Hooper, L. V. Antimicrobial defense of the Intestine. *Immunity* **42**, 28–39 (2015). 25607457 10.1016/j.immuni.2014.12.028

[385] McDonough, K. A. & Rodriguez, A. The myriad roles of cyclic AMP in microbial pathogens: from signal to sword. *Nat. Rev. Microbiol.* **10**, 27–38 (2012). 10.1038/nrmicro2688 PMC3785115 22080930

[386] Friedman, E. S. et al. Microbes vs. chemistry in the origin of the anaerobic gut lumen. *Proc. Natl Acad. Sci.* **115**, 4170–4175 (2018). 29610310 10.1073/pnas.1718635115 PMC5910840

[387] Litvak, Y., Byndloss, M. X. & Bäumler, A. J. Colonocyte metabolism shapes the gut microbiota. *Science* **362**, eaat9076 (2018). 30498100 10.1126/science.aat9076 PMC6296223

[388] Yang, H. et al. ABO genotype alters the gut microbiota by regulating GalNAc levels in pigs. *Nature* **606**, 358–367 (2022). 35477154 10.1038/s41586-022-04769-z PMC9157047

[389] Zhernakova, D. V. et al. Host genetic regulation of human gut microbial structural variation. *Nature* **625**, 813–821 (2024). 38172637 10.1038/s41586-023-06893-w PMC10808065

[390] Berry, D. et al. Host-compound foraging by intestinal microbiota revealed by single-cell stable isotope probing. *Proc. Natl Acad. Sci.* **110**, 4720–4725 (2013). 23487774 10.1073/pnas.1219247110 PMC3607026

[391] Cani, P. D., Depommier, C., Derrien, M., Everard, A. & de Vos, W. M. Akkermansia muciniphila: paradigm for next-generation beneficial microorganisms. *Nat. Rev. Gastroenterol. Hepatol*. **19**, 625–637 (2022). 10.1038/s41575-022-00631-9 35641786

[392] Schwerd, T. et al. NOX1 loss-of-function genetic variants in patients with inflammatory bowel disease. *Mucosal Immunol.* **11**, 562–574 (2018). 29091079 10.1038/mi.2017.74 PMC5924597

[393] Murdoch, C. C. & Skaar, E. P. Nutritional immunity: the battle for nutrient metals at the host–pathogen interface. *Nat. Rev. Microbiol*. **20**, 657–670. 10.1038/s41579-022-00745-6 (2022). 10.1038/s41579-022-00745-6 PMC9153222 35641670

[394] Bessman, N. J. et al. Dendritic cell–derived hepcidin sequesters iron from the microbiota to promote mucosal healing. *Science* **368**, 186–189 (2020). 32273468 10.1126/science.aau6481 PMC7724573

[395] Van Niel, G., D’Angelo, G. & Raposo, G. Shedding light on the cell biology of extracellular vesicles. *Nat. Rev. Mol. Cell Biol.* **19**, 213–228 (2018). 29339798 10.1038/nrm.2017.125

[396] Malmuthuge, N. & Guan, L. L. Noncoding RNAs: regulatory molecules of host–microbiome crosstalk. *Trends Microbiol.* **29**, 713–724 (2021). 33419590 10.1016/j.tim.2020.12.003

[397] Liu, S. et al. The host shapes the gut microbiota via fecal MicroRNA. *Cell Host Microbe* **19**, 32–43 (2016). 26764595 10.1016/j.chom.2015.12.005 PMC4847146

[398] Zhu, Z. et al. Gut microbiota regulate tumor metastasis via circRNA/miRNA networks. *Gut Microbes* **12**, 1788891 (2020). 32686598 10.1080/19490976.2020.1788891 PMC7524358

[399] Abdullah, S. T. et al. Role of circular RNAs and gut microbiome in gastrointestinal cancers and therapeutic targets. *Non-Coding RNA Res.* **9**, 236–252 (2024). 10.1016/j.ncrna.2023.12.002 PMC10771991 38192436

[400] Lyte, M. & Ernst, S. Catecholamine induced growth of gram negative bacteria. *Life Sci.* **50**, 203–212 (1992). 1731173 10.1016/0024-3205(92)90273-r

[401] Lyte, M. The role of microbial endocrinology in infectious disease. *J. Endocrinol.* **137**, 343–345 (1993). 8371072 10.1677/joe.0.1370343

[402] Gao, A. et al. Sexual dimorphism in glucose metabolism is shaped by androgen-driven gut microbiome. *Nat. Commun.* **12**, 7080 (2021). 34873153 10.1038/s41467-021-27187-7 PMC8648805

[403] Ibrahim, A. et al. Colitis‐induced colorectal cancer and intestinal epithelial estrogen receptor beta impact gut microbiota diversity. *Int. J. Cancer* **144**, 3086–3098 (2019). 30515752 10.1002/ijc.32037 PMC6519213

[404] Guan, Z. et al. Estrogen deficiency induces bone loss through the gut microbiota. *Pharmacol. Res.* **196**, 106930 (2023). 37722518 10.1016/j.phrs.2023.106930

[405] Li, J.-Y. et al. Sex steroid deficiency–associated bone loss is microbiota dependent and prevented by probiotics. *J. Clin. Invest.* **126**, 2049–2063 (2016). 27111232 10.1172/JCI86062 PMC4887186

[406] Hiller, C. C. et al. Influence of catecholamines on biofilm formation by Salmonella Enteritidis. *Microb. Pathog.* **130**, 54–58 (2019). 30831229 10.1016/j.micpath.2019.02.032

[407] Asano, Y. et al. Critical role of gut microbiota in the production of biologically active, free catecholamines in the gut lumen of mice. *Am. J. Physiol.—Gastrointest. Liver Physiol.* **303**, G1288–G1295 (2012). 23064760 10.1152/ajpgi.00341.2012

[408] Yang, D., Kong, Y., Sun, W., Kong, W. & Shi, Y. A dopamine-responsive signal transduction controls transcription of Salmonella enterica serovar typhimurium virulence genes. *mBio* **10**, e02772–18 (2019). 30992361 10.1128/mBio.02772-18 PMC6469979

[409] Scheiman, J. et al. Meta-omics analysis of elite athletes identifies a performance-enhancing microbe that functions via lactate metabolism. *Nat. Med.* **25**, 1104–1109 (2019). 31235964 10.1038/s41591-019-0485-4 PMC7368972

[410] Priest, C. & Tontonoz, P. Inter-organ cross-talk in metabolic syndrome. *Nat. Metab.* **1**, 1177–1188 (2019). 32694672 10.1038/s42255-019-0145-5

[411] Koren, O. et al. Host remodeling of the gut microbiome and metabolic changes during pregnancy. *Cell* **150**, 470–480 (2012). 22863002 10.1016/j.cell.2012.07.008 PMC3505857

[412] Tarracchini, C. et al. Genetic strategies for sex-biased persistence of gut microbes across human life. *Nat. Commun.* **14**, 4220 (2023). 37452041 10.1038/s41467-023-39931-2 PMC10349097

[413] Brown, K. et al. Microbiota alters the metabolome in an age- and sex-dependent manner in mice. *Nat. Commun.* **14**, 1348 (2023). 36906623 10.1038/s41467-023-37055-1 PMC10008592

[414] Mayneris-Perxachs, J. et al. Gut microbiota steroid sexual dimorphism and its impact on gonadal steroids: influences of obesity and menopausal status. *Microbiome* **8**, 136 (2020). 32951609 10.1186/s40168-020-00913-x PMC7504665

[415] Ghosh, T. S., Shanahan, F. & O’Toole, P. W. The gut microbiome as a modulator of healthy ageing. *Nat. Rev. Gastroenterol. Hepatol.* **19**, 565–584 (2022). 35468952 10.1038/s41575-022-00605-x PMC9035980

[416] Goodrich, J. K. et al. Human genetics shape the gut microbiome. *Cell* **159**, 789–799 (2014). 25417156 10.1016/j.cell.2014.09.053 PMC4255478

[417] Deschasaux, M. et al. Depicting the composition of gut microbiota in a population with varied ethnic origins but shared geography. *Nat. Med.* **24**, 1526–1531 (2018). 30150717 10.1038/s41591-018-0160-1

[418] Sen, P. et al. Microbiota and sleep: awakening the gut feeling. *Trends Mol. Med.* **27**, 935–945 (2021). 34364787 10.1016/j.molmed.2021.07.004

[419] Almand, A. T. et al. The influence of perceived stress on the human microbiome. *BMC Res. Notes* **15**, 193 (2022). 35659718 10.1186/s13104-022-06066-4 PMC9164568

[420] Dohnalová, L. et al. A microbiome-dependent gut–brain pathway regulates motivation for exercise. *Nature* **612**, 739–747 (2022). 36517598 10.1038/s41586-022-05525-z PMC11162758

[421] Thirion, F. et al. The gut microbiota in multiple sclerosis varies with disease activity. *Genome Med.* **15**, 1 (2023). 36604748 10.1186/s13073-022-01148-1 PMC9814178

[422] Wang, X. et al. Aberrant gut microbiota alters host metabolome and impacts renal failure in humans and rodents. *Gut* **69**, 2131–2142 (2020). 32241904 10.1136/gutjnl-2019-319766 PMC7677483

[423] Yannakoulia, M. & Scarmeas, N. Diets. *N. Engl. J. Med.* **390**, 2098–2106 (2024). 38865662 10.1056/NEJMra2211889

[424] Singh, R. K. et al. Influence of diet on the gut microbiome and implications for human health. *J. Transl. Med.* **15**, 73 (2017). 28388917 10.1186/s12967-017-1175-y PMC5385025

[425] Wu, G. D. et al. Linking long-term dietary patterns with gut microbial enterotypes. *Science* **334**, 105–108 (2011). 21885731 10.1126/science.1208344 PMC3368382

[426] Zmora, N., Suez, J. & Elinav, E. You are what you eat: diet, health and the gut microbiota. *Nat. Rev. Gastroenterol. Hepatol.* **16**, 35–56 (2019). 30262901 10.1038/s41575-018-0061-2

[427] De Filippo, C. et al. Impact of diet in shaping gut microbiota revealed by a comparative study in children from Europe and rural Africa. *Proc. Natl Acad. Sci.* **107**, 14691–14696 (2010). 20679230 10.1073/pnas.1005963107 PMC2930426

[428] Bourdeau-Julien, I. et al. The diet rapidly and differentially affects the gut microbiota and host lipid mediators in a healthy population. *Microbiome* **11**, 26 (2023). 36774515 10.1186/s40168-023-01469-2 PMC9921707

[429] David, L. A. et al. Diet rapidly and reproducibly alters the human gut microbiome. *Nature* **505**, 559–563 (2014). 24336217 10.1038/nature12820 PMC3957428

[430] Chen, J. et al. A high-fat diet promotes cancer progression by inducing gut microbiota–mediated leucine production and PMN-MDSC differentiation. *Proc. Natl Acad. Sci.* **121**, e2306776121 (2024). 38709933 10.1073/pnas.2306776121 PMC11098111

[431] Dohrn, G. Gut microbes linked to fatty diet drive tumour growth. *Nature*10.1038/d41586-024-01443-4 (2024). 10.1038/d41586-024-01443-4 38755304

[432] Suez, J. et al. Artificial sweeteners induce glucose intolerance by altering the gut microbiota. *Nature* **514**, 181–186 (2014). 25231862 10.1038/nature13793

[433] Makki, K., Deehan, E. C., Walter, J. & Bäckhed, F. The impact of dietary fiber on gut microbiota in host health and disease. *Cell Host Microbe* **23**, 705–715 (2018). 29902436 10.1016/j.chom.2018.05.012

[434] Ze, X., Duncan, S. H., Louis, P. & Flint, H. J. *Ruminococcus bromii* is a keystone species for the degradation of resistant starch in the human colon. *ISME J.* **6**, 1535–1543 (2012). 22343308 10.1038/ismej.2012.4 PMC3400402

[435] Shigehisa, A. et al. Characterization of a bifidobacterial system that utilizes galacto-oligosaccharides. *Microbiology* **161**, 1463–1470 (2015). 25903756 10.1099/mic.0.000100 PMC4635504

[436] Clark, R. L. et al. Design of synthetic human gut microbiome assembly and butyrate production. *Nat. Commun.* **12**, 3254 (2021). 34059668 10.1038/s41467-021-22938-y PMC8166853

[437] Crost, E. H. et al. Mechanistic Insights Into the Cross-Feeding of Ruminococcus gnavus and Ruminococcus bromii on Host and Dietary Carbohydrates. *Front. Microbiol.* **9**, 2558 (2018). 30455672 10.3389/fmicb.2018.02558 PMC6231298

[438] Nie, Q. et al. Targeted modification of gut microbiota and related metabolites via dietary fiber. *Carbohydr. Polym.* **316**, 120986 (2023). 37321707 10.1016/j.carbpol.2023.120986

[439] Rodriguez, C. I. et al. Curated and harmonized gut microbiome 16S rRNA amplicon data from dietary fiber intervention studies in humans. *Sci. Data* **10**, 346 (2023). 37268699 10.1038/s41597-023-02254-4 PMC10238384

[440] Zhang, J. et al. Diet mediate the impact of host habitat on gut microbiome and influence clinical indexes by modulating gut microbes and serum metabolites. *Adv. Sci.* **11**, 2310068 (2024). 10.1002/advs.202310068 PMC11109649 38477427

[441] Zinöcker, M. & Lindseth, I. The western diet–microbiome-host interaction and its role in metabolic disease. *Nutrients* **10**, 365 (2018). 29562591 10.3390/nu10030365 PMC5872783

[442] Latorre-Pérez, A. et al. The Spanish gut microbiome reveals links between microorganisms and Mediterranean diet. *Sci. Rep.* **11**, 21602 (2021). 34759297 10.1038/s41598-021-01002-1 PMC8580991

[443] Meslier, V. et al. Mediterranean diet intervention in overweight and obese subjects lowers plasma cholesterol and causes changes in the gut microbiome and metabolome independently of energy intake. *Gut* **69**, 1258–1268 (2020). 32075887 10.1136/gutjnl-2019-320438 PMC7306983

[444] Ben-Yacov, O. et al. Gut microbiome modulates the effects of a personalised postprandial-targeting (PPT) diet on cardiometabolic markers: a diet intervention in pre-diabetes. *Gut* 72, 1486–1496 (2023). 10.1136/gutjnl-2022-329201 PMC10359530 37137684

[445] Wang, D. D. et al. The gut microbiome modulates the protective association between a Mediterranean diet and cardiometabolic disease risk. *Nat. Med.* **27**, 333–343 (2021). 33574608 10.1038/s41591-020-01223-3 PMC8186452

[446] Hansen, T. H. et al. Impact of a vegan diet on the human salivary microbiota. *Sci. Rep.* **8**, 5847 (2018). 29643500 10.1038/s41598-018-24207-3 PMC5895596

[447] Landberg, R. & Hanhineva, K. Biomarkers of a Healthy Nordic Diet—From Dietary Exposure Biomarkers to Microbiota Signatures in the Metabolome. *Nutrients* **12**, 27 (2019). 31877633 10.3390/nu12010027 PMC7019922

[448] Jama, H. A., Beale, A., Shihata, W. A. & Marques, F. Z. The effect of diet on hypertensive pathology: is there a link via gut microbiota-driven immunometabolism? *Cardiovasc. Res.* **115**, 1435–1447 (2019). 30951169 10.1093/cvr/cvz091

[449] Xue, Z. et al. The bamboo-eating giant panda harbors a carnivore-like gut microbiota, with excessive seasonal variations. *mBio* **6**, e00022–15 (2015). 25991678 10.1128/mBio.00022-15 PMC4442137

[450] Ang, Q. Y. et al. Ketogenic diets alter the gut microbiome resulting in decreased intestinal Th17 cells. *Cell* **181**, 1263–1275.e16 (2020). 32437658 10.1016/j.cell.2020.04.027 PMC7293577

[451] Miao, Z. et al. Gut microbiota signatures of long-term and short-term plant-based dietary pattern and cardiometabolic health: a prospective cohort study. *BMC Med.* **20**, 204 (2022). 35701845 10.1186/s12916-022-02402-4 PMC9199182

[452] Von Schwartzenberg, R. J. et al. Caloric restriction disrupts the microbiota and colonization resistance. *Nature* **595**, 272–277 (2021). 34163067 10.1038/s41586-021-03663-4 PMC8959578

[453] Ismail, I. H. et al. Dietary patterns in childhood and their effect on gut microbiota—an Asian perspective on atopy risk. *J. Allergy Clin. Immunol.* **146**, 1005–1007 (2020). 32860819 10.1016/j.jaci.2020.05.057

[454] Sonnenburg, J. L. & Bäckhed, F. Diet–microbiota interactions as moderators of human metabolism. *Nature* **535**, 56–64 (2016). 27383980 10.1038/nature18846 PMC5991619

[455] Rothschild, D. et al. Environment dominates over host genetics in shaping human gut microbiota. *Nature* **555**, 210–215 (2018). 29489753 10.1038/nature25973

[456] Gacesa, R. et al. Environmental factors shaping the gut microbiome in a Dutch population. *Nature* **604**, 732–739 (2022). 35418674 10.1038/s41586-022-04567-7

[457] Linz, B. et al. An African origin for the intimate association between humans and Helicobacter pylori. *Nature* **445**, 915–918 (2007). 17287725 10.1038/nature05562 PMC1847463

[458] Falush, D. et al. Traces of human migrations in *Helicobacter pylori* populations. *Science* **299**, 1582–1585 (2003). 12624269 10.1126/science.1080857

[459] Thorpe, H. A. et al. Repeated out-of-Africa expansions of Helicobacter pylori driven by replacement of deleterious mutations. *Nat. Commun.* **13**, 6842 (2022). 36369175 10.1038/s41467-022-34475-3 PMC9652371

[460] Coelho, L. P. et al. Towards the biogeography of prokaryotic genes. *Nature* **601**, 252–256 (2022). 34912116 10.1038/s41586-021-04233-4 PMC7613196

[461] Gupta, V. K., Paul, S. & Dutta, C. Geography, ethnicity or subsistence-specific variations in human microbiome composition and diversity. *Front. Microbiol.* **8**, 1162 (2017). 28690602 10.3389/fmicb.2017.01162 PMC5481955

[462] Lin, L. et al. The airway microbiome mediates the interaction between environmental exposure and respiratory health in humans. *Nat. Med*. **29**, 1750–1759. 10.1038/s41591-023-02424-2 (2023). 10.1038/s41591-023-02424-2 37349537

[463] Ying, S. et al. The influence of age and gender on skin-associated microbial communities in urban and rural human populations. *PLoS ONE* **10**, e0141842 (2015). 26510185 10.1371/journal.pone.0141842 PMC4624872

[464] Zhang, Y.-D. et al. Greenspace and human microbiota: a systematic review. *Environ. Int.* **187**, 108662 (2024). 38653130 10.1016/j.envint.2024.108662

[465] He, Y. et al. Regional variation limits applications of healthy gut microbiome reference ranges and disease models. *Nat. Med.* **24**, 1532–1535 (2018). 30150716 10.1038/s41591-018-0164-x

[466] Tierney, B. T. et al. Longitudinal multi-omics analysis of host microbiome architecture and immune responses during short-term spaceflight. *Nat. Microbiol*. **9**, 1661–1675 (2024). 10.1038/s41564-024-01635-8 PMC11222149 38862604

[467] Weersma, R. K., Zhernakova, A. & Fu, J. Interaction between drugs and the gut microbiome. *Gut* **69**, 1510–1519 (2020). 32409589 10.1136/gutjnl-2019-320204 PMC7398478

[468] Lam, K. N., Alexander, M. & Turnbaugh, P. J. Precision medicine goes microscopic: engineering the microbiome to improve drug outcomes. *Cell Host Microbe* **26**, 22–34 (2019). 31295421 10.1016/j.chom.2019.06.011 PMC6709864

[469] Wollein Waldetoft, K., Sundius, S., Kuske, R. & Brown, S. P. Defining the benefits of antibiotic resistance in commensals and the scope for resistance optimization. *mBio* **14**, e01349–22 (2023). 36475750 10.1128/mbio.01349-22 PMC9972992

[470] Bhattarai, S. K. et al. Commensal antimicrobial resistance mediates microbiome resilience to antibiotic disruption. *Sci. Transl. Med.* **16**, eadi9711 (2024). 38232140 10.1126/scitranslmed.adi9711 PMC11017772

[471] Wang, Y. et al. Antidepressants can induce mutation and enhance persistence toward multiple antibiotics. *Proc. Natl Acad. Sci.* **120**, e2208344120 (2023). 36689653 10.1073/pnas.2208344120 PMC9945972

[472] Maier, L. et al. Extensive impact of non-antibiotic drugs on human gut bacteria. *Nature* **555**, 623–628 (2018). 29555994 10.1038/nature25979 PMC6108420

[473] Jackson, M. A. et al. Proton pump inhibitors alter the composition of the gut microbiota. *Gut* **65**, 749–756 (2016). 26719299 10.1136/gutjnl-2015-310861 PMC4853574

[474] Imhann, F. et al. Proton pump inhibitors affect the gut microbiome. *Gut* **65**, 740–748 (2016). 26657899 10.1136/gutjnl-2015-310376 PMC4853569

[475] De La Cuesta-Zuluaga, J. et al. Metformin is associated with higher relative abundance of mucin-degrading *Akkermansia muciniphila* and several short-chain fatty acid–producing microbiota in the gut. *Diabetes Care* **40**, 54–62 (2017). 27999002 10.2337/dc16-1324

[476] Forslund, S. K. et al. Combinatorial, additive and dose-dependent drug–microbiome associations. *Nature* **600**, 500–505 (2021). 34880489 10.1038/s41586-021-04177-9

[477] Sousa, T. et al. On the colonic bacterial metabolism of azo-bonded prodrugsof 5-aminosalicylic acid. *J. Pharm. Sci.* **103**, 3171–3175 (2014). 25091594 10.1002/jps.24103

[478] Haiser, H. J. et al. Predicting and manipulating cardiac drug inactivation by the human gut bacterium *Eggerthella lenta*. *Science* **341**, 295–298 (2013). 23869020 10.1126/science.1235872 PMC3736355

[479] Klatt, N. R. et al. Vaginal bacteria modify HIV tenofovir microbicide efficacy in African women. *Science* **356**, 938–945 (2017). 28572388 10.1126/science.aai9383

[480] Wallace, B. D. et al. Alleviating cancer drug toxicity by inhibiting a bacterial enzyme. *Science* **330**, 831–835 (2010). 21051639 10.1126/science.1191175 PMC3110694

[481] Zimmermann, M., Zimmermann-Kogadeeva, M., Wegmann, R. & Goodman, A. L. Mapping human microbiome drug metabolism by gut bacteria and their genes. *Nature* **570**, 462–467 (2019). 31158845 10.1038/s41586-019-1291-3 PMC6597290

[482] Heinken, A. et al. Genome-scale metabolic reconstruction of 7,302 human microorganisms for personalized medicine. *Nat. Biotechnol*. **41**, 1320–1331 (2023). 10.1038/s41587-022-01628-0 PMC10497413 36658342

[483] Wang, D.-R., Wu, X.-L. & Sun, Y.-L. Therapeutic targets and biomarkers of tumor immunotherapy: response versus non-response. *Signal Transduct. Target. Ther.* **7**, 331 (2022). 36123348 10.1038/s41392-022-01136-2 PMC9485144

[484] Huang, J. et al. Effects of microbiota on anticancer drugs: current knowledge and potential applications. *eBioMedicine* **83**, 104197 (2022). 35933808 10.1016/j.ebiom.2022.104197 PMC9358415

[485] Stein-Thoeringer, C. K. et al. A non-antibiotic-disrupted gut microbiome is associated with clinical responses to CD19-CAR-T cell cancer immunotherapy. *Nat. Med*. **29**, 906–916 (2023). 10.1038/s41591-023-02234-6 PMC10121864 36914893

[486] Faust, K. & Raes, J. Microbial interactions: from networks to models. *Nat. Rev. Microbiol.* **10**, 538–550 (2012). 22796884 10.1038/nrmicro2832

[487] Jiang, M.-Z. et al. Gut microbial interactions based on network construction and bacterial pairwise cultivation. *Sci. China Life Sci*. **67**,1751–1762 (2024). 10.1007/s11427-023-2537-0 38600293

[488] Krishnan, N., Csiszár, V., Móri, T. F. & Garay, J. Genesis of ectosymbiotic features based on commensalistic syntrophy. *Sci. Rep.* **14**, 1366 (2024). 38228651 10.1038/s41598-023-47211-8 PMC10791676

[489] Culp, E. J. & Goodman, A. L. Cross-feeding in the gut microbiome: ecology and mechanisms. *Cell Host Microbe* **31**, 485–499 (2023). 37054671 10.1016/j.chom.2023.03.016 PMC10125260

[490] Maree, M. et al. Natural transformation allows transfer of SCCmec-mediated methicillin resistance in Staphylococcus aureus biofilms. *Nat. Commun.* **13**, 2477 (2022). 35513365 10.1038/s41467-022-29877-2 PMC9072672

[491] Zhong, Q. et al. Episymbiotic Saccharibacteria TM7x modulates the susceptibility of its host bacteria to phage infection and promotes their coexistence. *Proc. Natl Acad. Sci.* **121**, e2319790121 (2024). 38593079 10.1073/pnas.2319790121 PMC11032452

[492] Lamkin, J. & Lavner, J. A. Antagonism and romantic relationships. in *The Handbook of Antagonism* 269–280 (Elsevier, 2019). 10.1016/B978-0-12-814627-9.00018-9.

[493] Clardy, J., Fischbach, M. A. & Currie, C. R. The natural history of antibiotics. *Curr. Biol.* **19**, R437–R441 (2009). 19515346 10.1016/j.cub.2009.04.001 PMC2731226

[494] Johnston, L. A. Competitive interactions between cells: death, growth, and geography. *Science* **324**, 1679–1682 (2009). 19556501 10.1126/science.1163862 PMC2736143

[495] Sørensen, M. E. S. et al. The role of exploitation in the establishment of mutualistic microbial symbioses. *FEMS Microbiol. Lett.* **366**, fnz148 (2019). 31271421 10.1093/femsle/fnz148 PMC6638607

[496] Shkoporov, A. N., Turkington, C. J. & Hill, C. Mutualistic interplay between bacteriophages and bacteria in the human gut. *Nat. Rev. Microbiol.* **20**, 737–749 (2022). 35773472 10.1038/s41579-022-00755-4

[497] Morin, M. A., Morrison, A. J., Harms, M. J. & Dutton, R. J. Higher-order interactions shape microbial interactions as microbial community complexity increases. *Sci. Rep.* **12**, 22640 (2022). 36587027 10.1038/s41598-022-25303-1 PMC9805437

[498] Amit, G. & Bashan, A. Top-down identification of keystone taxa in the microbiome. *Nat. Commun.* **14**, 3951 (2023). 37402745 10.1038/s41467-023-39459-5 PMC10319726

[499] Paine, R. T. Food web complexity and species diversity. *Am. Nat.* **100**, 65–75 (1966).

[500] Libertucci, J. & Young, V. B. The role of the microbiota in infectious diseases. *Nat. Microbiol.* **4**, 35–45 (2018). 30546094 10.1038/s41564-018-0278-4

[501] Newton, W. L., Weinstein, P. P. & Jones, M. F. A comparison of the development of some rat and mouse helminths in germfree and conventional guinea pigs. *Ann. N. Y. Acad. Sci.* **78**, 290–307 (2006). 10.1111/j.1749-6632.1959.tb53114.x 14426855

[502] Phillips, B. P. et al. Studies on the ameba-bacteria relationship in amebiasis; comparative results of the intracecal inoculation of germfree, monocontaminated, and conventional guinea pigs with Entamoeba histolytica. *Am. J. Trop. Med. Hyg.* **4**, 675–692 (1955). 13238723

[503] Phillips, B. P. & Wolfe, P. A. The use of germfree guinea pigs in studies on the microbial interrelationships in amoebiasis*. *Ann. N. Y. Acad. Sci.* **78**, 308–314 (2006). 10.1111/j.1749-6632.1959.tb53115.x 14432606

[504] Lin, T.-L. et al. Gut microbiota dysbiosis-related susceptibility to nontuberculous mycobacterial lung disease. *Gut Microbes* **16**, 2361490 (2024). 38860456 10.1080/19490976.2024.2361490 PMC11174134

[505] Wahl, A. et al. A germ-free humanized mouse model shows the contribution of resident microbiota to human-specific pathogen infection. *Nat. Biotechnol*. **42**, 905–915 (2023). 10.1038/s41587-023-01906-5 PMC11073568 37563299

[506] Gosmann, C. et al. Lactobacillus-deficient cervicovaginal bacterial communities are associated with increased HIV acquisition in young South African women. *Immunity* **46**, 29–37 (2017). 28087240 10.1016/j.immuni.2016.12.013 PMC5270628

[507] Sulaiman, I. et al. Microbial signatures in the lower airways of mechanically ventilated COVID-19 patients associated with poor clinical outcome. *Nat. Microbiol.* **6**, 1245–1258 (2021). 34465900 10.1038/s41564-021-00961-5 PMC8484067

[508] Gao, M. et al. Characterization of the human oropharyngeal microbiomes in SARS‐CoV‐2 infection and recovery patients. *Adv. Sci.* **8**, 2102785 (2021). 10.1002/advs.202102785 PMC8529429 34423593

[509] Zuo, T., Wu, X., Wen, W. & Lan, P. Gut microbiome alterations in COVID-19. *Genomics Proteom. Bioinform.* **19**, 679–688 (2021). 10.1016/j.gpb.2021.09.004 PMC8478109 34560321

[510] Xiao, M. et al. Metatranscriptomic analysis of host response and vaginal microbiome of patients with severe COVID-19. *Sci. China Life Sci.* **65**, 1473–1476 (2022). 35441283 10.1007/s11427-021-2091-0 PMC9017960

[511] Chen, Y. et al. Six-month follow-up of gut microbiota richness in patients with COVID-19. *Gut* **71**, 222–225 (2022). 33833065 10.1136/gutjnl-2021-324090 PMC8666823

[512] Yeoh, Y. K. et al. Gut microbiota composition reflects disease severity and dysfunctional immune responses in patients with COVID-19. *Gut* **70**, 698–706 (2021). 33431578 10.1136/gutjnl-2020-323020 PMC7804842

[513] Patrier, J. et al. Oropharyngeal and intestinal concentrations of opportunistic pathogens are independently associated with death of SARS-CoV-2 critically ill adults. *Crit. Care* **26**, 300 (2022). 36192756 10.1186/s13054-022-04164-0 PMC9527714

[514] McDonald, B. et al. Programing of an intravascular immune firewall by the gut microbiota protects against pathogen dissemination during infection. *Cell Host Microbe* **28**, 660–668.e4 (2020). 32810440 10.1016/j.chom.2020.07.014

[515] Zhang, F. et al. Gut microbiota in COVID-19: key microbial changes, potential mechanisms and clinical applications. *Nat. Rev. Gastroenterol. Hepatol.* **20**, 323–337 (2023). 36271144 10.1038/s41575-022-00698-4 PMC9589856

[516] Li, J., Richards, E. M., Handberg, E. M., Pepine, C. J. & Raizada, M. K. Butyrate regulates COVID-19–relevant genes in gut epithelial organoids from normotensive rats. *Hypertension* **77** (2021). 10.1161/HYPERTENSIONAHA.120.16647 PMC7810239 33439735

[517] Zhang, F. et al. Prolonged impairment of short-chain fatty acid and l-isoleucine biosynthesis in gut microbiome in patients with COVID-19. *Gastroenterology* **162**, 548–561.e4 (2022). 34687739 10.1053/j.gastro.2021.10.013 PMC8529231

[518] De Nies, L. et al. Altered infective competence of the human gut microbiome in COVID-19. *Microbiome* **11**, 46 (2023). 36894986 10.1186/s40168-023-01472-7 PMC9995755

[519] Mizrahi, B. et al. Long covid outcomes at one year after mild SARS-CoV-2 infection: nationwide cohort study. *BMJ* **380**, e072529 (2023). 10.1136/bmj-2022-072529 PMC9832503 36631153

[520] McIntosh, C. M., Chen, L., Shaiber, A., Eren, A. M. & Alegre, M.-L. Gut microbes contribute to variation in solid organ transplant outcomes in mice. *Microbiome* **6**, 96 (2018). 29793539 10.1186/s40168-018-0474-8 PMC5968713

[521] Swarte, J. C. et al. Gut microbiome dysbiosis is associated with increased mortality after solid organ transplantation. *Sci. Transl. Med.* **14**, eabn7566 (2022). 36044594 10.1126/scitranslmed.abn7566

[522] Bocci, V. The neglected organ: bacterial flora has a crucial immunostimulatory role. *Perspect. Biol. Med.* **35**, 251–260 (1992). 1557302 10.1353/pbm.1992.0004

[523] O’Hara, A. M. & Shanahan, F. The gut flora as a forgotten organ. *EMBO Rep.* **7**, 688–693 (2006). 16819463 10.1038/sj.embor.7400731 PMC1500832

[524] Burcelin, R. et al. Metagenome and metabolism: the tissue microbiota hypothesis: Burcelin et al. *Diabetes Obes. Metab.* **15**, 61–70 (2013). 24003922 10.1111/dom.12157

[525] Byndloss, M. X. & Bäumler, A. J. The germ-organ theory of non-communicable diseases. *Nat. Rev. Microbiol.* **16**, 103–110 (2018). 29307890 10.1038/nrmicro.2017.158

[526] Fucarino, A. et al. The microbiota is not an organ: introducing the muco-microbiotic layer as a novel morphofunctional. *Struct. Anat.* **1**, 186–203 (2022).

[527] Chakaroun, R. M., Olsson, L. M. & Bäckhed, F. The potential of tailoring the gut microbiome to prevent and treat cardiometabolic disease. *Nat. Rev. Cardiol*. **20**, 217–235 (2022). 10.1038/s41569-022-00771-0 36241728

[528] Valles-Colomer, M. et al. Cardiometabolic health, diet and the gut microbiome: a meta-omics perspective. *Nat. Med.* **29**, 551–561 (2023). 36932240 10.1038/s41591-023-02260-4 PMC11258867

[529] Tonelli, A., Lumngwena, E. N. & Ntusi, N. A. B. The oral microbiome in the pathophysiology of cardiovascular disease. *Nat. Rev. Cardiol.* **20**, 386–403 (2023). 36624275 10.1038/s41569-022-00825-3

[530] Violi, F. et al. Gut-derived low-grade endotoxaemia, atherothrombosis and cardiovascular disease. *Nat. Rev. Cardiol.* **20**, 24–37 (2023). 35840742 10.1038/s41569-022-00737-2 PMC9284488

[531] Brown, J. M. & Hazen, S. L. Microbial modulation of cardiovascular disease. *Nat. Rev. Microbiol.* **16**, 171–181 (2018). 29307889 10.1038/nrmicro.2017.149 PMC5885760

[532] Lyon, J. The lung microbiome: key to respiratory ills? *JAMA* **317**, 1713 (2017). 28403451 10.1001/jama.2017.3023

[533] Budden, K. F. et al. Functional effects of the microbiota in chronic respiratory disease. *Lancet Respir. Med.* **7**, 907–920 (2019). 30975495 10.1016/S2213-2600(18)30510-1

[534] Barcik, W., Boutin, R. C. T., Sokolowska, M. & Finlay, B. B. The role of lung and gut microbiota in the pathology of asthma. *Immunity* **52**, 241–255 (2020). 32075727 10.1016/j.immuni.2020.01.007 PMC7128389

[535] Neish, A. S. Microbes in gastrointestinal health and disease. *Gastroenterology* **136**, 65–80 (2009). 19026645 10.1053/j.gastro.2008.10.080 PMC2892787

[536] Sorboni, S. G., Moghaddam, H. S., Jafarzadeh-Esfehani, R. & Soleimanpour, S. A comprehensive review on the role of the gut microbiome in human neurological disorders. *Clin. Microbiol. Rev.* **35**, e00338–20 (2022). 34985325 10.1128/CMR.00338-20 PMC8729913

[537] Fang, P., Kazmi, S. A., Jameson, K. G. & Hsiao, E. Y. The microbiome as a modifier of neurodegenerative disease risk. *Cell Host Microbe* **28**, 201–222 (2020). 32791113 10.1016/j.chom.2020.06.008 PMC7430034

[538] Willyard, C. How gut microbes could drive brain disorders. *Nature* **590**, 22–25 (2021). 33536656 10.1038/d41586-021-00260-3

[539] Agirman, G. & Hsiao, E. Y. SnapShot: the microbiota-gut-brain axis. *Cell* **184**, 2524–2524.e1 (2021). 33930299 10.1016/j.cell.2021.03.022

[540] Cryan, J. F. et al. The microbiota-gut-brain axis. *Physiol. Rev.* **99**, 1877–2013 (2019). 31460832 10.1152/physrev.00018.2018

[541] Qi, X., Yun, C., Pang, Y. & Qiao, J. The impact of the gut microbiota on the reproductive and metabolic endocrine system. *Gut Microbes* **13**, 1894070 (2021). 33722164 10.1080/19490976.2021.1894070 PMC7971312

[542] Van Hul, M. & Cani, P. D. The gut microbiota in obesity and weight management: microbes as friends or foe? *Nat. Rev. Endocrinol.* **19**, 258–271 (2023). 36650295 10.1038/s41574-022-00794-0

[543] Fan, Y. & Pedersen, O. Gut microbiota in human metabolic health and disease. *Nat. Rev. Microbiol.* **19**, 55–71 (2021). 32887946 10.1038/s41579-020-0433-9

[544] Hajishengallis, G., Lamont, R. J. & Koo, H. Oral polymicrobial communities: assembly, function, and impact on diseases. *Cell Host Microbe* **31**, 528–538 (2023). 36933557 10.1016/j.chom.2023.02.009 PMC10101935

[545] Tuganbaev, T., Yoshida, K. & Honda, K. The effects of oral microbiota on health. *Science* **376**, 934–936 (2022). 35617380 10.1126/science.abn1890

[546] Stacy, A. & Belkaid, Y. Microbial guardians of skin health. *Science* **363**, 227–228 (2019). 30655428 10.1126/science.aat4326

[547] Miyauchi, E., Shimokawa, C., Steimle, A., Desai, M. S. & Ohno, H. The impact of the gut microbiome on extra-intestinal autoimmune diseases. *Nat. Rev. Immunol*. **23**, 9–23 (2022). 10.1038/s41577-022-00727-y 35534624

[548] Alcazar, C. G.-M. et al. The association between early-life gut microbiota and childhood respiratory diseases: a systematic review. *Lancet Microbe* **3**, e867–e880 (2022). 35988549 10.1016/S2666-5247(22)00184-7 PMC10499762

[549] Ruff, W. E., Greiling, T. M. & Kriegel, M. A. Host–microbiota interactions in immune-mediated diseases. *Nat. Rev. Microbiol.* **18**, 521–538 (2020). 32457482 10.1038/s41579-020-0367-2

[550] Knauf, F., Brewer, J. R. & Flavell, R. A. Immunity, microbiota and kidney disease. *Nat. Rev. Nephrol.* **15**, 263–274 (2019). 30796361 10.1038/s41581-019-0118-7

[551] Shoubridge, A. P. et al. The gut microbiome and mental health: advances in research and emerging priorities. *Mol. Psychiatry* **27**, 1908–1919 (2022). 35236957 10.1038/s41380-022-01479-w

[552] Liu, L. & Shah, K. The potential of the gut microbiome to reshape the cancer therapy paradigm: a review. *JAMA Oncol.* **8**, 1059 (2022). 35482355 10.1001/jamaoncol.2022.0494

[553] Park, E. M. et al. Targeting the gut and tumor microbiota in cancer. *Nat. Med.* **28**, 690–703 (2022). 35440726 10.1038/s41591-022-01779-2

[554] Sepich-Poore, G. D. et al. The microbiome and human cancer. *Science* **371**, eabc4552 (2021). 33766858 10.1126/science.abc4552 PMC8767999

[555] Gowen, R., Gamal, A., Di Martino, L., McCormick, T. S. & Ghannoum, M. A. Modulating the microbiome for Crohn’s disease treatment. *Gastroenterology* **164**, 828–840 (2023). 36702360 10.1053/j.gastro.2023.01.017 PMC10152883

[556] Motta, J.-P., Wallace, J. L., Buret, A. G., Deraison, C. & Vergnolle, N. Gastrointestinal biofilms in health and disease. *Nat. Rev. Gastroenterol. Hepatol.* **18**, 314–334 (2021). 33510461 10.1038/s41575-020-00397-y

[557] Arumugam, M. et al. Enterotypes of the human gut microbiome. *Nature* **473**, 174–180 (2011). 21508958 10.1038/nature09944 PMC3728647

[558] Costea, P. I. et al. Enterotypes in the landscape of gut microbial community composition. *Nat. Microbiol.* **3**, 8–16 (2018). 29255284 10.1038/s41564-017-0072-8 PMC5832044

[559] Zhong, H. et al. Impact of early events and lifestyle on the gut microbiota and metabolic phenotypes in young school-age children. *Microbiome* **7**, 2 (2019). 30609941 10.1186/s40168-018-0608-z PMC6320620

[560] Knights, D. et al. Rethinking “Enterotypes”. *Cell Host Microbe* **16**, 433–437 (2014). 25299329 10.1016/j.chom.2014.09.013 PMC5558460

[561] Lai, S. et al. Enterotypes of the human gut mycobiome. *Microbiome* **11**, 179 (2023). 37563687 10.1186/s40168-023-01586-y PMC10416509

[562] Bourdeau, R. W. et al. Acoustic reporter genes for noninvasive imaging of microorganisms in mammalian hosts. *Nature* **553**, 86–90 (2018). 29300010 10.1038/nature25021 PMC5920530

[563] Willis, J. R. et al. Citizen science charts two major “stomatotypes” in the oral microbiome of adolescents and reveals links with habits and drinking water composition. *Microbiome* **6**, 218 (2018). 30522523 10.1186/s40168-018-0592-3 PMC6284318

[564] Gajer, P. et al. Temporal dynamics of the human vaginal microbiota. *Sci. Transl. Med*. **4** (2012). 10.1126/scitranslmed.3003605 PMC3722878 22553250

[565] Adolph, E. F. Early concepts of physiological regulations. *Physiol. Rev.* **41**, 737–770 (1961). 13859463 10.1152/physrev.1961.41.4.737

[566] Cannon, W. B. Organization for physiological homeostasis. *Physiol. Rev.* **9**, 399–431 (1929).

[567] Baptista, V. Starting physiology: understanding homeostasis. *Adv. Physiol. Educ.* **30**, 263–264 (2006). 17108259 10.1152/advan.00075.2006

[568] Kotas, M. E. & Medzhitov, R. Homeostasis, inflammation, and disease susceptibility. *Cell* **160**, 816–827 (2015). 25723161 10.1016/j.cell.2015.02.010 PMC4369762

[569] Davies, K. J. A. Adaptive homeostasis. *Mol. Asp. Med.* **49**, 1–7 (2016). 10.1016/j.mam.2016.04.007 PMC4868097 27112802

[570] Khakisahneh, S., Zhang, X.-Y., Nouri, Z. & Wang, D.-H. Gut microbiota and host thermoregulation in response to ambient temperature fluctuations. *mSystems* **5**, e00514–e00520 (2020). 33082280 10.1128/mSystems.00514-20 PMC7577294

[571] Bongers, K. S. et al. The gut microbiome modulates body temperature both in sepsis and health. *Am. J. Respir. Crit. Care Med*. **207**,1030–1041 (2022). 10.1164/rccm.202201-0161OC PMC10112447 36378114

[572] Li, B. et al. Microbiota depletion impairs thermogenesis of brown adipose tissue and browning of white adipose tissue. *Cell Rep.* **26**, 2720–2737.e5 (2019). 30840893 10.1016/j.celrep.2019.02.015

[573] Gurven, M. et al. Rapidly declining body temperature in a tropical human population. *Sci. Adv.* **6**, eabc6599 (2020). 33115745 10.1126/sciadv.abc6599 PMC7608783

[574] Protsiv, M., Ley, C., Lankester, J., Hastie, T. & Parsonnet, J. Decreasing human body temperature in the United States since the Industrial Revolution. *eLife* **9**, e49555 (2020). 31908267 10.7554/eLife.49555 PMC6946399

[575] Liu, Y. et al. A widely distributed gene cluster compensates for uricase loss in hominids. *Cell* **186**, 3400–3413.e20 (2023). 37541197 10.1016/j.cell.2023.06.010 PMC10421625

[576] Kasahara, K. et al. Gut bacterial metabolism contributes to host global purine homeostasis. *Cell Host Microbe* **31**, 1038–1053.e10 (2023). 37279756 10.1016/j.chom.2023.05.011 PMC10311284

[577] Johnson, R. J. et al. Do thrifty genes exist? Revisiting uricase. *Obesity* **30**, 1917–1926 (2022). 36150210 10.1002/oby.23540 PMC9512363

[578] Chang, B. S. W. Ancient insights into uric acid metabolism in primates. *Proc. Natl Acad. Sci.* **111**, 3657–3658 (2014). 24556992 10.1073/pnas.1401037111 PMC3956182

[579] Muller, P. A. et al. Microbiota-modulated CART ^+^ enteric neurons autonomously regulate blood glucose. *Science* **370**, 314–321 (2020). 32855216 10.1126/science.abd6176 PMC7886298

[580] Suez, J. et al. Personalized microbiome-driven effects of non-nutritive sweeteners on human glucose tolerance. *Cell* **185**, 3307–3328.e19 (2022). 35987213 10.1016/j.cell.2022.07.016

[581] Chen, B.-Y. et al. Characteristics and correlations of the oral and gut fungal microbiome with hypertension. *Microbiol. Spectr.* **11**, e01956–22 (2023). 36475759 10.1128/spectrum.01956-22 PMC9927468

[582] O’Donnell, J. A., Zheng, T., Meric, G. & Marques, F. Z. The gut microbiome and hypertension. *Nat. Rev. Nephrol.* **19**, 153–167 (2023). 36631562 10.1038/s41581-022-00654-0

[583] Bushyhead, D. & Quigley, E. M. M. Small intestinal bacterial overgrowth—pathophysiology and its implications for definition and management. *Gastroenterology* **163**, 593–607 (2022). 35398346 10.1053/j.gastro.2022.04.002

[584] Hayase, E. et al. Mucus-degrading Bacteroides link carbapenems to aggravated graft-versus-host disease. *Cell* **185**, 3705–3719.e14 (2022). 36179667 10.1016/j.cell.2022.09.007 PMC9542352

[585] Lu, Q. & Stappenbeck, T. S. Local barriers configure systemic communications between the host and microbiota. *Science* **376**, 950–955 (2022). 35617395 10.1126/science.abo2366

[586] Anhê, F. F., Barra, N. G., Cavallari, J. F., Henriksbo, B. D. & Schertzer, J. D. Metabolic endotoxemia is dictated by the type of lipopolysaccharide. *Cell Rep.* **36**, 109691 (2021). 34525353 10.1016/j.celrep.2021.109691

[587] Marshall, J. C. The microbiology of multiple organ failure: the proximal gastrointestinal tract as an occult reservoir of pathogens. *Arch. Surg.* **123**, 309 (1988). 3341911 10.1001/archsurg.1988.01400270043006

[588] Berg, R. Bacterial translocation from the gastrointestinal tract. *Trends Microbiol.* **3**, 149–154 (1995). 7613757 10.1016/s0966-842x(00)88906-4

[589] Meisel, M. et al. Microbial signals drive pre-leukaemic myeloproliferation in a Tet2-deficient host. *Nature* **557**, 580–584 (2018). 29769727 10.1038/s41586-018-0125-z PMC6238954

[590] Peng, S. et al. CRB1-associated retinal degeneration is dependent on bacterial translocation from the gut. *Cell* **187**, 1387–1401.e13 (2024). 38412859 10.1016/j.cell.2024.01.040

[591] Massier, L. et al. Adipose tissue derived bacteria are associated with inflammation in obesity and type 2 diabetes. *Gut* **69**, 1796–1806 (2020). 32317332 10.1136/gutjnl-2019-320118

[592] Anhê, F. F. et al. Type 2 diabetes influences bacterial tissue compartmentalisation in human obesity. *Nat. Metab.* **2**, 233–242 (2020). 32694777 10.1038/s42255-020-0178-9

[593] Simpson, B. W. & Trent, M. S. Pushing the envelope: LPS modifications and their consequences. *Nat. Rev. Microbiol.* **17**, 403–416 (2019). 31142822 10.1038/s41579-019-0201-x PMC6913091

[594] Lasselin, J. et al. Comparison of bacterial lipopolysaccharide-induced sickness behavior in rodents and humans: Relevance for symptoms of anxiety and depression. *Neurosci. Biobehav. Rev.* **115**, 15–24 (2020). 32433924 10.1016/j.neubiorev.2020.05.001

[595] Page, M. J., Kell, D. B. & Pretorius, E. The role of lipopolysaccharide-induced cell signalling in chronic inflammation. *Chronic Stress* **6**, 247054702210763 (2022). 10.1177/24705470221076390 PMC8829728 35155966

[596] Cani, P. D. et al. Metabolic endotoxemia initiates obesity and insulin resistance. *Diabetes* **56**, 1761–1772 (2007). 17456850 10.2337/db06-1491

[597] Yang, Y. et al. Within-host evolution of a gut pathobiont facilitates liver translocation. *Nature* **607**, 563–570 (2022). 35831502 10.1038/s41586-022-04949-x PMC9308686

[598] Hoyles, L. et al. Regulation of blood–brain barrier integrity by microbiome-associated methylamines and cognition by trimethylamine N-oxide. *Microbiome* **9**, 235 (2021). 34836554 10.1186/s40168-021-01181-z PMC8626999

[599] Zhu, W. et al. Gut microbial metabolite TMAO enhances platelet hyperreactivity and thrombosis risk. *Cell* **165**, 111–124 (2016). 26972052 10.1016/j.cell.2016.02.011 PMC4862743

[600] Benson, T. W. et al. Gut microbiota–derived trimethylamine n-oxide contributes to abdominal aortic aneurysm through inflammatory and apoptotic mechanisms. *Circulation* **147**, 1079–1096 (2023). 37011073 10.1161/CIRCULATIONAHA.122.060573 PMC10071415

[601] Wang, Z. et al. Gut flora metabolism of phosphatidylcholine promotes cardiovascular disease. *Nature* **472**, 57–63 (2011). 21475195 10.1038/nature09922 PMC3086762

[602] Wang, H. et al. The microbial metabolite trimethylamine N-oxide promotes antitumor immunity in triple-negative breast cancer. *Cell Metab.* **34**, 581–594.e8 (2022). 35278352 10.1016/j.cmet.2022.02.010

[603] Mirji, G. et al. The microbiome-derived metabolite TMAO drives immune activation and boosts responses to immune checkpoint blockade in pancreatic cancer. *Sci. Immunol.* **7**, eabn0704 (2022). 36083892 10.1126/sciimmunol.abn0704 PMC9925043

[604] Needham, B. D. et al. A gut-derived metabolite alters brain activity and anxiety behaviour in mice. *Nature* **602**, 647–653 (2022). 35165440 10.1038/s41586-022-04396-8 PMC9170029

[605] Qi, X. et al. Gut microbiota–bile acid–interleukin-22 axis orchestrates polycystic ovary syndrome. *Nat. Med.* **25**, 1225–1233 (2019). 31332392 10.1038/s41591-019-0509-0 PMC7376369

[606] Yun, C. et al. The microbial metabolite agmatine acts as an FXR agonist to promote polycystic ovary syndrome in female mice. *Nat. Metab.* **6**, 947–962 (2024). 38769396 10.1038/s42255-024-01041-8

[607] Trabelsi, M.-S. et al. Farnesoid X receptor inhibits glucagon-like peptide-1 production by enteroendocrine L cells. *Nat. Commun.* **6**, 7629 (2015). 26134028 10.1038/ncomms8629 PMC4579574

[608] Koh, A. et al. Microbially produced imidazole propionate impairs insulin signaling through mTORC1. *Cell* **175**, 947–961.e17 (2018). 30401435 10.1016/j.cell.2018.09.055

[609] Molinaro, A. et al. Imidazole propionate is increased in diabetes and associated with dietary patterns and altered microbial ecology. *Nat. Commun.* **11**, 5881 (2020). 33208748 10.1038/s41467-020-19589-w PMC7676231

[610] White, P. J. & Newgard, C. B. Branched-chain amino acids in disease. *Science* **363**, 582–583 (2019). 30733403 10.1126/science.aav0558 PMC9940269

[611] Nemet, I. et al. A cardiovascular disease-linked gut microbial metabolite acts via adrenergic receptors. *Cell* **180**, 862–877.e22 (2020). 32142679 10.1016/j.cell.2020.02.016 PMC7402401

[612] Tennoune, N. et al. Bacterial ClpB heat-shock protein, an antigen-mimetic of the anorexigenic peptide α-MSH, at the origin of eating disorders. *Transl. Psychiatry* **4**, e458–e458 (2014). 25290265 10.1038/tp.2014.98 PMC4350527

[613] Gil-Cruz, C. et al. Microbiota-derived peptide mimics drive lethal inflammatory cardiomyopathy. *Science* **366**, 881–886 (2019). 31727837 10.1126/science.aav3487

[614] Wang, K. et al. Microbial-host-isozyme analyses reveal microbial DPP4 as a potential antidiabetic target. *Science* **381**, eadd5787 (2023). 37535747 10.1126/science.add5787

[615] Granton, E. et al. Biofilm exopolysaccharides alter sensory-neuron-mediated sickness during lung infection. *Cell* **187**, 1874–1888.e14 (2024). 38518773 10.1016/j.cell.2024.03.001

[616] Valles-Colomer, M. et al. The person-to-person transmission landscape of the gut and oral microbiomes. *Nature* **614**, 125–135 (2023). 36653448 10.1038/s41586-022-05620-1 PMC9892008

[617] Finlay, B. B. CIFAR Humans, & the Microbiome. Are noncommunicable diseases communicable? *Science* **367**, 250–251 (2020). 31949069 10.1126/science.aaz3834

[618] Sarkar, A. et al. Microbial transmission in the social microbiome and host health and disease. *Cell* **187**, 17–43 (2024). 38181740 10.1016/j.cell.2023.12.014 PMC10958648

[619] Chong, B. et al. Trends and predictions of malnutrition and obesity in 204 countries and territories: an analysis of the Global Burden of Disease Study 2019. *eClinicalMedicine* **57**, 101850 (2023). 36864983 10.1016/j.eclinm.2023.101850 PMC9971264

[620] Lindstrom, M. et al. Global burden of cardiovascular diseases and risks collaboration, 1990–2021. *J. Am. Coll. Cardiol.* **80**, 2372–2425 (2022). 36517116 10.1016/j.jacc.2022.11.001

[621] Wang, Y. et al. Global burden of digestive diseases: a systematic analysis of the global burden of diseases study, 1990–2019. *Gastroenterology* **165**, 773–783.e15 (2023). 10.1053/j.gastro.2023.05.050 37302558

[622] Ong, K. L. et al. Global, regional, and national burden of diabetes from 1990 to 2021, with projections of prevalence to 2050: a systematic analysis for the Global Burden of Disease Study 2021. *Lancet* **402**, 203–234 (2023). 10.1016/S0140-6736(23)01301-6 PMC10364581 37356446

[623] Tran, K. B. et al. The global burden of cancer attributable to risk factors, 2010–19: a systematic analysis for the Global Burden of Disease Study 2019. *Lancet* **400**, 563–591 (2022). 35988567 10.1016/S0140-6736(22)01438-6 PMC9395583

[624] Fink, G., Tediosi, F. & Felder, S. Burden of Covid-19 restrictions: National, regional and global estimates. *eClinicalMedicine* **45**, 101305 (2022). 35224471 10.1016/j.eclinm.2022.101305 PMC8856030

[625] Bello, M. G. D., Knight, R., Gilbert, J. A. & Blaser, M. J. Preserving microbial diversity. *Science* **362**, 33–34 (2018). 30287652 10.1126/science.aau8816

[626] Allsup, C. M., George, I. & Lankau, R. A. Shifting microbial communities can enhance tree tolerance to changing climates. *Science* **380**, 835–840 (2023). 37228219 10.1126/science.adf2027

[627] Raaijmakers, J. M. & Kiers, E. T. Rewilding plant microbiomes. *Science* **378**, 599–600 (2022). 36356130 10.1126/science.abn6350

[628] Reardon, S. Faecal transplants could help preserve vulnerable species. *Nature* **558**, 173–174 (2018). 29895922 10.1038/d41586-018-05352-1

[629] Cahan, E. As superbugs flourish, bacteriophage therapy recaptures researchers’ interest. *JAMA* **329**, 781 (2023). 36811929 10.1001/jama.2022.17756

[630] Strathdee, S. A., Hatfull, G. F., Mutalik, V. K. & Schooley, R. T. Phage therapy: from biological mechanisms to future directions. *Cell* **186**, 17–31 (2023). 36608652 10.1016/j.cell.2022.11.017 PMC9827498

[631] Bayfield, O. W. et al. Structural atlas of a human gut crassvirus. *Nature* **617**, 409–416 (2023). 37138077 10.1038/s41586-023-06019-2 PMC10172136

[632] Benler, S. et al. Thousands of previously unknown phages discovered in whole-community human gut metagenomes. *Microbiome* **9**, 78 (2021). 33781338 10.1186/s40168-021-01017-w PMC8008677

[633] Nayfach, S. et al. Metagenomic compendium of 189,680 DNA viruses from the human gut microbiome. *Nat. Microbiol.* **6**, 960–970 (2021). 34168315 10.1038/s41564-021-00928-6 PMC8241571

[634] Camarillo-Guerrero, L. F., Almeida, A., Rangel-Pineros, G., Finn, R. D. & Lawley, T. D. Massive expansion of human gut bacteriophage diversity. *Cell* **184**, 1098–1109.e9 (2021). 33606979 10.1016/j.cell.2021.01.029 PMC7895897

[635] Shkoporov, A. N. & Hill, C. Bacteriophages of the human gut: the “known unknown” of the microbiome. *Cell Host Microbe* **25**, 195–209 (2019). 30763534 10.1016/j.chom.2019.01.017

[636] Yutin, N. et al. Discovery of an expansive bacteriophage family that includes the most abundant viruses from the human gut. *Nat. Microbiol.* **3**, 38–46 (2017). 29133882 10.1038/s41564-017-0053-y PMC5736458

[637] Zhang, F., Aschenbrenner, D., Yoo, J. Y. & Zuo, T. The gut mycobiome in health, disease, and clinical applications in association with the gut bacterial microbiome assembly. *Lancet Microbe* **3**, e969–e983 (2022). 36182668 10.1016/S2666-5247(22)00203-8

[638] Kong, H. H. & Segre, J. A. Cultivating fungal research. *Science* **368**, 365–366 (2020). 32327584 10.1126/science.aaz8086 PMC10506662

[639] Thomas, C. M., Desmond-Le Quéméner, E., Gribaldo, S. & Borrel, G. Factors shaping the abundance and diversity of the gut archaeome across the animal kingdom. *Nat. Commun.* **13**, 3358 (2022). 35688919 10.1038/s41467-022-31038-4 PMC9187648

[640] Hoegenauer, C., Hammer, H. F., Mahnert, A. & Moissl-Eichinger, C. Methanogenic archaea in the human gastrointestinal tract. *Nat. Rev. Gastroenterol. Hepatol.* **19**, 805–813 (2022). 36050385 10.1038/s41575-022-00673-z

[641] Pennisi, E. Survey of archaea in the body reveals other microbial guests. *Science* **358**, 983–983 (2017). 29170214 10.1126/science.358.6366.983

[642] Borrel, G., Brugère, J.-F., Gribaldo, S., Schmitz, R. A. & Moissl-Eichinger, C. The host-associated archaeome. *Nat. Rev. Microbiol.* **18**, 622–636 (2020). 32690877 10.1038/s41579-020-0407-y

[643] Yang, L., Li, A., Wang, Y. & Zhang, Y. Intratumoral microbiota: roles in cancer initiation, development and therapeutic efficacy. *Signal Transduct. Target. Ther.* **8**, 35 (2023). 36646684 10.1038/s41392-022-01304-4 PMC9842669

[644] Bullman, S. The intratumoral microbiota: from microniches to single cells. *Cell* **186**, 1532–1534 (2023). 37059062 10.1016/j.cell.2023.03.012

[645] Swanton, C. et al. Embracing cancer complexity: Hallmarks of systemic disease. *Cell* **187**, 1589–1616 (2024). 38552609 10.1016/j.cell.2024.02.009 PMC12077170

[646] Cao, Y. et al. Intratumoural microbiota: a new frontier in cancer development and therapy. *Signal Transduct. Target. Ther.* **9**, 15 (2024). 38195689 10.1038/s41392-023-01693-0 PMC10776793

[647] Blake, S. J., Wolf, Y., Boursi, B. & Lynn, D. J. Role of the microbiota in response to and recovery from cancer therapy. *Nat. Rev. Immunol.* **24**, 308–325 (2024). 37932511 10.1038/s41577-023-00951-0

[648] Bai, X. et al. Engineering the gut microbiome. *Nat. Rev. Bioeng.* **1**, 665–679 (2023).

[649] Kurtz, C. B. et al. An engineered *E. coli* Nissle improves hyperammonemia and survival in mice and shows dose-dependent exposure in healthy humans. *Sci. Transl. Med.* **11**, eaau7975 (2019). 30651324 10.1126/scitranslmed.aau7975

[650] Durmusoglu, D. et al. In situ biomanufacturing of small molecules in the mammalian gut by probiotic *Saccharomyces boulardii*. *ACS Synth. Biol.* **10**, 1039–1052 (2021). 33843197 10.1021/acssynbio.0c00562 PMC12977008

[651] Selle, K. et al. In vivo targeting of clostridioides difficile using phage-delivered CRISPR-Cas3 antimicrobials. *mBio* **11**, e00019–e00020 (2020). 32156803 10.1128/mBio.00019-20 PMC7064742

[652] Lam, K. N. et al. Phage-delivered CRISPR-Cas9 for strain-specific depletion and genomic deletions in the gut microbiome. *Cell Rep.* **37**, 109930 (2021). 34731631 10.1016/j.celrep.2021.109930 PMC8591988

[653] Patel, J. R., Oh, J., Wang, S., Crawford, J. M. & Isaacs, F. J. Cross-kingdom expression of synthetic genetic elements promotes discovery of metabolites in the human microbiome. *Cell* **185**, 1487–1505.e14 (2022). 35366417 10.1016/j.cell.2022.03.008 PMC10619838

[654] Wang, L. et al. Engineering consortia by polymeric microbial swarmbots. *Nat. Commun.* **13**, 3879 (2022). 35790722 10.1038/s41467-022-31467-1 PMC9256712

[655] Robinson, J. M. et al. Twenty important research questions in microbial exposure and social equity. *mSystems* **7**, e01240–21 (2022). 35089060 10.1128/msystems.01240-21 PMC8725600

[656] Oduaran, O. H. et al. Microbiome research in Africa must be based on equitable partnerships. *Nat. Med*. 10.1038/s41591-024-03026-2 (2024). 10.1038/s41591-024-03026-2 38783138

[657] Hou, K. et al. Microbiota in health and diseases. *Signal Transduct. Target. Ther.* **7**, 135 (2022). 35461318 10.1038/s41392-022-00974-4 PMC9034083

[658] Sender, R., Fuchs, S. & Milo, R. Are We really vastly outnumbered? revisiting the ratio of bacterial to host cells in humans. *Cell* **164**, 337–340 (2016). 26824647 10.1016/j.cell.2016.01.013

[659] Sender, R., Fuchs, S. & Milo, R. Revised estimates for the number of human and bacteria cells in the body. *PLoS Biol.* **14**, e1002533 (2016). 27541692 10.1371/journal.pbio.1002533 PMC4991899

[660] Porcari, S. et al. Key determinants of success in fecal microbiota transplantation: from microbiome to clinic. *Cell Host Microbe* **31**, 712–733 (2023). 37167953 10.1016/j.chom.2023.03.020

[661] Ng, R. W. et al. Revisiting the donor screening protocol of faecal microbiota transplantation (FMT): a systematic review. *Gut* **73**,1029–1031 (2023). 10.1136/gutjnl-2023-329515 PMC11103291 37142392

[662] Gulati, A. S., Nicholson, M. R., Khoruts, A. & Kahn, S. A. Fecal microbiota transplantation across the lifespan: balancing efficacy, safety, and innovation. *Am. J. Gastroenterol.* **118**, 435–439 (2023). 36580630 10.14309/ajg.0000000000002167 PMC9992015

[663] Allegretti, J. R., Mullish, B. H., Kelly, C. & Fischer, M. The evolution of the use of faecal microbiota transplantation and emerging therapeutic indications. *Lancet* **394**, 420–431 (2019). 31379333 10.1016/S0140-6736(19)31266-8

[664] Schmidt, T. S. B. et al. Drivers and determinants of strain dynamics following fecal microbiota transplantation. *Nat. Med.* **28**, 1902–1912 (2022). 36109636 10.1038/s41591-022-01913-0 PMC9499871

[665] Shen, N. T. et al. Timely use of probiotics in hospitalized adults prevents clostridium difficile infection: a systematic review with meta-regression analysis. *Gastroenterology* **152**, 1889–1900.e9 (2017). 28192108 10.1053/j.gastro.2017.02.003

[666] Cohen, C. R. et al. Randomized trial of lactin-V to prevent recurrence of bacterial vaginosis. *N. Engl. J. Med.* **382**, 1906–1915 (2020). 32402161 10.1056/NEJMoa1915254 PMC7362958

[667] Goldenberg, J. Z., Mertz, D. & Johnston, B. C. Probiotics to prevent *Clostridium difficile* infection in patients receiving antibiotics. *JAMA* **320**, 499 (2018). 30027207 10.1001/jama.2018.9064

[668] Suez, J., Zmora, N., Segal, E. & Elinav, E. The pros, cons, and many unknowns of probiotics. *Nat. Med.* **25**, 716–729 (2019). 31061539 10.1038/s41591-019-0439-x

[669] Piewngam, P. et al. Probiotic for pathogen-specific Staphylococcus aureus decolonisation in Thailand: a phase 2, double-blind, randomised, placebo-controlled trial. *Lancet Microbe* **4**, e75–e83 (2023). 36646104 10.1016/S2666-5247(22)00322-6 PMC9932624

[670] Sanders, M. E., Merenstein, D. J., Reid, G., Gibson, G. R. & Rastall, R. A. Probiotics and prebiotics in intestinal health and disease: from biology to the clinic. *Nat. Rev. Gastroenterol. Hepatol.* **16**, 605–616 (2019). 31296969 10.1038/s41575-019-0173-3

[671] Fagnant, H. S. et al. Orally ingested probiotic, prebiotic, and synbiotic interventions as countermeasures for gastrointestinal tract infections in nonelderly adults: a systematic review and meta-analysis. *Adv. Nutr.* **14**, 539–554 (2023). 36822240 10.1016/j.advnut.2023.02.002 PMC10201658

[672] Swanson, K. S. et al. The International Scientific Association for Probiotics and Prebiotics (ISAPP) consensus statement on the definition and scope of synbiotics. *Nat. Rev. Gastroenterol. Hepatol.* **17**, 687–701 (2020). 32826966 10.1038/s41575-020-0344-2 PMC7581511

[673] Salminen, S. et al. Author Correction: The International Scientific Association of Probiotics and Prebiotics (ISAPP) consensus statement on the definition and scope of postbiotics. *Nat. Rev. Gastroenterol. Hepatol.* **19**, 551–551 (2022). 35538163 10.1038/s41575-022-00628-4 PMC9329092

[674] Wargo, J. A. Modulating gut microbes. *Science* **369**, 1302–1303 (2020). 32913089 10.1126/science.abc3965

[675] Raffatellu, M. Learning from bacterial competition in the host to develop antimicrobials. *Nat. Med.* **24**, 1097–1103 (2018). 30082869 10.1038/s41591-018-0145-0

[676] Sorbara, M. T. & Pamer, E. G. Microbiome-based therapeutics. *Nat. Rev. Microbiol.* **20**, 365–380 (2022). 34992261 10.1038/s41579-021-00667-9

[677] Renz, H. & Skevaki, C. Early life microbial exposures and allergy risks: opportunities for prevention. *Nat. Rev. Immunol.* **21**, 177–191 (2021). 32918062 10.1038/s41577-020-00420-y

[678] Vatanen, T. et al. The human gut microbiome in early-onset type 1 diabetes from the TEDDY study. *Nature* **562**, 589–594 (2018). 30356183 10.1038/s41586-018-0620-2 PMC6296767

[679] Dong, D. et al. *Clostridioides difficile* aggravates dextran sulfate solution (DSS)-induced colitis by shaping the gut microbiota and promoting neutrophil recruitment. *Gut Microbes* **15**, 2192478 (2023). 36951545 10.1080/19490976.2023.2192478 PMC10038061

[680] Yadegar, A. et al. Beneficial effects of fecal microbiota transplantation in recurrent Clostridioides difficile infection. *Cell Host Microbe* **31**, 695–711 (2023). 37167952 10.1016/j.chom.2023.03.019 PMC10966711

[681] Cohen, S. H. et al. Extended follow-up of microbiome therapeutic SER-109 through 24 weeks for recurrent *Clostridioides difficile* infection in a randomized clinical trial. *JAMA* **328**, 2062 (2022). 36260754 10.1001/jama.2022.16476 PMC9582966

[682] Walter, J. & Shanahan, F. Fecal microbiota-based treatment for recurrent Clostridioides difficile infection. *Cell* **186**, 1087 (2023). 36931236 10.1016/j.cell.2023.02.034

[683] Sims, M. D. et al. Safety and tolerability of SER-109 as an investigational microbiome therapeutic in adults with recurrent *Clostridioides difficile* infection: a phase 3, open-label, single-arm trial. *JAMA Netw. Open* **6**, e2255758 (2023). 36780159 10.1001/jamanetworkopen.2022.55758 PMC9926325

[684] Sehgal, K., Cifu, A. S. & Khanna, S. Treatment of *Clostridioides difficile* Infection. *JAMA* **328**, 881 (2022). 35939317 10.1001/jama.2022.12251

[685] Louie, T. et al. VE303, a defined bacterial consortium, for prevention of recurrent *Clostridioides difficile* infection: a randomized clinical trial. *JAMA* **329**, 1356 (2023). 37060545 10.1001/jama.2023.4314 PMC10105904

[686] Garey, K. W. et al. Assessment of quality of life among patients with recurrent clostridioides difficile infection treated with investigational oral microbiome therapeutic SER-109: secondary analysis of a randomized clinical trial. *JAMA Netw. Open* **6**, e2253570 (2023). 36716031 10.1001/jamanetworkopen.2022.53570 PMC9887497

[687] Khoruts, A., Staley, C. & Sadowsky, M. J. Faecal microbiota transplantation for Clostridioides difficile: mechanisms and pharmacology. *Nat. Rev. Gastroenterol. Hepatol.* **18**, 67–80 (2021). 32843743 10.1038/s41575-020-0350-4

[688] Wu, R., Xiong, R., Li, Y., Chen, J. & Yan, R. Gut microbiome, metabolome, host immunity associated with inflammatory bowel disease and intervention of fecal microbiota transplantation. *J. Autoimmun*. **141**, 103062 (2023). 10.1016/j.jaut.2023.103062 37246133

[689] Paramsothy, S. et al. Multidonor intensive faecal microbiota transplantation for active ulcerative colitis: a randomised placebo-controlled trial. *Lancet* **389**, 1218–1228 (2017). 28214091 10.1016/S0140-6736(17)30182-4

[690] Baumgart, D. C. & Le Berre, C. Newer Biologic and small-molecule therapies for inflammatory bowel disease. *N. Engl. J. Med.* **385**, 1302–1315 (2021). 34587387 10.1056/NEJMra1907607

[691] Chang, J. T. Pathophysiology of inflammatory bowel diseases. *N. Engl. J. Med.* **383**, 2652–2664 (2020). 33382932 10.1056/NEJMra2002697

[692] Caruso, R., Lo, B. C. & Núñez, G. Host–microbiota interactions in inflammatory bowel disease. *Nat. Rev. Immunol.* **20**, 411–426 (2020). 32005980 10.1038/s41577-019-0268-7

[693] Schirmer, M., Garner, A., Vlamakis, H. & Xavier, R. J. Microbial genes and pathways in inflammatory bowel disease. *Nat. Rev. Microbiol.* **17**, 497–511 (2019). 31249397 10.1038/s41579-019-0213-6 PMC6759048

[694] Lavelle, A. & Sokol, H. Gut microbiota-derived metabolites as key actors in inflammatory bowel disease. *Nat. Rev. Gastroenterol. Hepatol.* **17**, 223–237 (2020). 32076145 10.1038/s41575-019-0258-z

[695] Lopetuso, L. R. et al. The first international Rome consensus conference on gut microbiota and faecal microbiota transplantation in inflammatory bowel disease. *Gut* **72**, 1642–1650 (2023). 10.1136/gutjnl-2023-329948 PMC10423477 37339849

[696] Xin, Y. et al. Fecal microbiota transplantation in the treatment of systemic lupus erythematosus: What we learnt from the explorative clinical trial. *J. Autoimmun*. **141**, 103058 (2023). 10.1016/j.jaut.2023.103058 37179170

[697] Yang, R., Chen, Z. & Cai, J. Fecal microbiota transplantation: Emerging applications in autoimmune diseases. *J. Autoimmun*. **141**, 103038 (2023). 10.1016/j.jaut.2023.103038 37117118

[698] Palmer, J. D. & Foster, K. R. Bacterial species rarely work together. *Science* **376**, 581–582 (2022). 35511986 10.1126/science.abn5093

[699] Smith, A. B. et al. Enterococci enhance Clostridioides difficile pathogenesis. *Nature* **611**, 780–786 (2022). 36385534 10.1038/s41586-022-05438-x PMC9691601

[700] Santus, W. et al. Mycobiota and diet-derived fungal xenosiderophores promote Salmonella gastrointestinal colonization. *Nat. Microbiol.* **7**, 2025–2038 (2022). 36411353 10.1038/s41564-022-01267-w PMC11981548

[701] Neumann, P. E. & Neumann, E. E. General histological woes: definition and classification of tissues. *Clin. Anat.* **34**, 794–801 (2021). 33909319 10.1002/ca.23741

[702] Terminology, F. I. C. on A. *Terminologia Histologica: International Terms for Human Cytology and Histology* (Lippincott Raven, 2008). 10.1111/j.1469-7580.2009.01093_1.x 19486203

[703] Aggarwal, N. et al. Microbiome and Human Health: Current Understanding, Engineering, and Enabling Technologies. *Chem. Rev*. **123**, 31–72 (2022). 10.1021/acs.chemrev.2c00431 PMC9837825 36317983

[704] Javan, G. T. et al. Human thanatomicrobiome succession and time since death. *Sci. Rep.* **6**, 29598 (2016). 27412051 10.1038/srep29598 PMC4944132

[705] Hasan, N. & Yang, H. Factors affecting the composition of the gut microbiota, and its modulation. *PeerJ* **7**, e7502 (2019). 31440436 10.7717/peerj.7502 PMC6699480

[706] Dimitriu, P. A. et al. New insights into the intrinsic and extrinsic factors that shape the human skin microbiome. *mBio* **10**, e00839–19 (2019). 31266865 10.1128/mBio.00839-19 PMC6606800

[707] Flandroy, L. et al. The impact of human activities and lifestyles on the interlinked microbiota and health of humans and of ecosystems. *Sci. Total Environ.* **627**, 1018–1038 (2018). 29426121 10.1016/j.scitotenv.2018.01.288

